# A Study to Decipher the Potential Effects of Butylphthalide against Central Nervous System Diseases Based on Network Pharmacology and Molecular Docking Integration Strategy

**DOI:** 10.1155/2021/6694698

**Published:** 2021-05-04

**Authors:** Qinqin Zhao, Bei Zheng, Pinpin Feng, Xiang Li

**Affiliations:** ^1^Department of Pharmacy, Tongde Hospital of Zhejiang Province, Hangzhou, Zhejiang Province, China; ^2^Zhejiang Academy of Traditional Chinese Medicine, Hangzhou, Zhejiang Province, China; ^3^School of Basic Medical Sciences and Forensic Medicine, Hangzhou Medical College, Hangzhou, Zhejiang, China

## Abstract

**Background:**

Butylphthalide (NBP), approved by the China National Medical Products Administration (NMPA) for the treatment of ischemic stroke (IS), showed pleiotropic potentials against central nervous system (CNS) diseases, including neuroprotection and cognitive deficits improvement. However, the effects and corresponding modes of action were not fully explored. This study was designed to investigate the potential of NBP against IS-associated CNS diseases based on network pharmacology (NP) and molecular docking (MD).

**Methods:**

IS was inputted as the index disease to retrieve the “associated diseases” in DisGeNET. Three-database-based IS genes were obtained and integrated (DisGeNET, Malacards, and OMIM). Then, IS-associated genes were identified by combining these genes. Meanwhile, PubMed references and online databases were applied to identify NBP target genes. The IS-related disease-disease association (DDA) network and NBP-disease regulation network were constructed and analyzed in Cytoscape. *In silico* MD and references were used to validate the binding affinity of NBP with critical targets and the potential of NBP against certain IS-related CNS disease regulation.

**Results:**

175 NBP target genes were obtained, while 312 IS-related disease genes were identified. 36 NBP target genes were predicted to be associated with IS-related CNS diseases, including Alzheimer's disease (AD), epilepsy, major depressive disorder (MDD), amyotrophic lateral sclerosis (ALS), and dementia. Six target genes (i.e., GRIN1, PTGIS, PTGES, ADRA1A, CDK5, and SULT1E1) indicating disease specificity index (DSI) >0.5 showed certain to good degree binding affinity with NBP, ranging from −9.2 to −6.7 kcal/mol. And the binding modes may be mainly related to hydrogen bonds and hydrophobic “bonds.” Further literature validations inferred that these critical NBP targets had a tight association with AD, epilepsy, ALS, and depression.

**Conclusions:**

Our study proposed a drug-target-disease integrated method to predict the drug repurposing potentials to associated diseases by application of NP and MD, which could be an attractive alternative to facilitate the development of CNS disease therapies. NBP may be promising and showed potentials to be repurposed for treatments for AD, epilepsy, ALS, and depression, and further investigations are warranted to be carefully designed and conducted.

## 1. Introduction

Brain/central nervous system (CNS) diseases are multifactorial and polygenic diseases, including stroke, Alzheimer's disease (AD), and epilepsy, which remain an urgent and unmet medical need nowadays. The WHO statistics titled “Global Health Estimates 2016: Estimated deaths by age, sex, and cause” showed that there were 56873805 deaths globally in which noncommunicable diseases (NCDs) contributed 71.29% (40545176 deaths). Currently, NCDs are the main reasons to cause deaths, and ischemic heart disease (IHD) and stroke are ranked 1^st^ and 2^nd^, respectively, among the top 10 causes of death. The data indicated that brain/CNS-related NCDs of stroke, neurological conditions, and brain/nervous system cancers contributed to 15% of deaths. Meanwhile, the proportions of IHD and stroke are 16.59% and 10.16%, respectively.

It has a high failure rate to develop therapies for brain/CNS-related NCDs, as AD, epilepsy, Parkinson's disease, and dementia, which will be costly, lengthy, and characterized by poor/no understanding of the underlying pathophysiology [1]. New strategies to develop drugs for CNS diseases treatments are urgently needed. Fortunately, multiple CNS-disease-related pathways have been identified, and CNS diseases were found to share common pathways, which will provide possible opportunities for applying existing drugs for other indications based on disease-disease associations (DDAs) [2]. Diseases with overlapping disease modules display similar underlying molecular mechanisms and clinical symptoms [3], which will favor us to find approved drugs of one indication to other indications based on DDAs.

As de novo discovering and developing new drugs are extremely tough works with time-consuming, high-cost, and low-success rate. For CNS drug development, it is particularly tough as researchers need to conquer the hurdles encountered, like the blood-brain barrier (BBB) and the translations from animal to human, etc. [4]. Drug repurposing or drug repositioning would be an attractive way to develop therapies for CNS diseases. While de novo drug development and approval require at least 12–16 years and 1-2 billion dollars, drug repositioning only needs 6 years and 300 million dollars. As most repurposed drugs have already passed the preclinical development and clinical testing, drug repositioning may facilitate the efficiency of drug development and it would be an important alternative to develop drugs for new indications [5]. Several examples have applied a drug repurposing strategy to accelerate drug development processes. Minocycline was repurposed for acute ischemic stroke (IS), as it showed effects of anti-inflammation and MMP-9 inhibition, which would be attractive to use in combination with tissue plasminogen activator (tPA) [6]. AD is a chronic and progressive neurodegenerative disease and one study reviewed priority candidate drugs for repositioning in AD [7], such as benperidol [8] and cinitapride [9]. Another study combining RNA sequencing and zebrafish model of seizures repurposed FDA-approved metformin, nifedipine, and pyrantel tartrate as candidate antiepilepsy drugs [10]. As dementia and type 2 diabetes (T2D) share some underlying pathophysiology, studies tried to repurpose T2D drugs for dementia treatment. Intranasal insulin, metformin, and GLP-1 receptor agonists are attractive candidate drugs; however, more studies are required before clinicians recommend candidate diabetes therapies for dementia [11]. Amyotrophic lateral sclerosis (ALS), as CNS rare disease, is a progressive fatal disorder. The free radical scavenger edaravone has been developed for the treatment of ALS [12]. In our previous study, we tried to find approved cardiovascular drugs for ischemic cerebrovascular disease based on disease-disease-associated prediction, which may provide a promising alternative to infer novel disease indications for known drugs [13]. DDAs link different diseases sharing common underlying molecular mechanisms together and provide beneficial information for drug repurposing investigation. What's more, the drug discovery paradigm has shifted from “magic bullet” to “multitarget drugs,” as polypharmacology is the nature of most ligand/drugs, and the proportion of drugs that in theory could be repositioned is about 75 [5, 14, 15]. In 2002, dl-3n-butylphthalide (NBP) proved its safety and therapeutic effect on cerebral ischemia and received approval from the National Medical Products Administration (NMPA) of China for the treatment of acute ischemic stroke. NBP has shown potentials for treatment in the field of CNS diseases; several investigations have reported the effects of NBP including neuroprotection and cognitive deficits improvement [16–18]. In 2018, NBP was granted orphan drug designation by the US FDA for the treatment of ALS.

In order to repurpose NBP for the treatment of IS-associated CNS diseases and to decipher the potential underlying mechanisms, our study identified NBP potential targets by integration of targets from public databases like PubMed, PharmMapper [19], DRAR-CPI [20], and SymMap [21], while IS and other IS-associated CNS disease-related targets were obtained from DisGeNET [21], Malacards [22], and OMIM [23]. As mentioned above, CNS diseases may share common pathways, and disease-associated genes can be collected from disease databases (DisGeNET, Malacards, and OMIM). Network pharmacology and *in silico* molecular docking were integrated to uncover the underlying modes of action.

**Figure 1:**
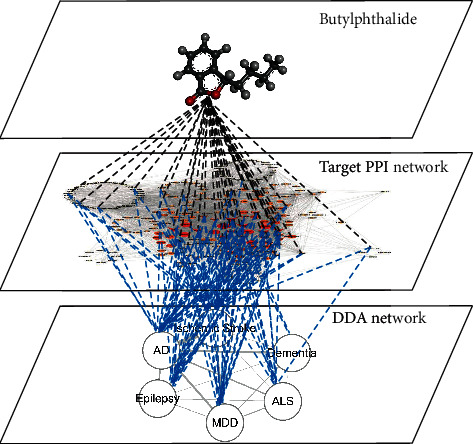
The three-layer drug-target-disease network of butylphthalide. PPI: protein-protein interaction; DDAs: disease-disease associations; AD: Alzheimer's disease; MDD: major depressive disorder; ALS: amyotrophic lateral sclerosis. The thickness of edges in the DDA network is in proportion to the number of shared target genes in the target PPI network.

The schematic diagram can be attained in Figure 1 which depicted the three-layer drug-target-diseases network of NBP. In summary, our study has proposed a drug-target-disease integrating method to predict the drug repurposing potentials to other associated diseases by application of network pharmacology and molecular docking, which could be an attractive alternative to facilitate CNS disease therapy development.

## 2. Materials and Methods

The network pharmacology and molecular modeling software used included AutoDock Tools (Version 1.5.6), AutoDock Vina software [24], LigPlot+ (Version 2.2), and Cytoscape 3.8.0 software [25].

### 2.1. NBP Potential Target Identification and Integration

NBP targets were collected and integrated from three resources, i.e., PubMed references, online database prediction (PharmMapper and DRAR-CPI), and SymMap. Firstly, butylphthalide was selected as a keyword to retrieve targets from PubMed by applying PALM-IST (https://www.hpppi.iicb.res.in/ctm/index.html) ranging from 2000 to 2014, while other targets from 2015 to 30^th^ May 2019 of NBP were obtained from PubMed with further reading and manually confirmation. Secondly, PharmMapper Server was applied to identify potential target candidates of NBP, and the .mol2 file was generated with ChemDraw and Chem3D software (version 17.1) according to the structure in PubChem (PubChem CID: 61361). In PharmMapper, the inclusion standard was Fit score ≥2 with Human species. DRAR-CPI was another database applied to predict potential targets of NBP through inputting a .mol file from Drugbank (ID DB12749). In DRAR-CPI, the putative target selection standard was docking score >−50 and *Z*′-score >−0.5. Targets from PharmMapper and DRAR-CPI were normalized, and their overlapped target genes were collected. Thirdly, SymMap database was used to retrieve potential targets of NBP with the keyword “Dl-3N-Butylphthalide.” Duplicated NBP target candidates from different sources were removed, and the left target genes were used for further analysis.

### 2.2. IS-Related Disease-Disease Association (DDA) Network

DisGeNET database was applied to retrieve IS-related diseases and their corresponding genes. “Ischemic stroke” was used as index-disease (Index-disease-id: C0948008), and the “associated diseases” were obtained. According to the number of shared genes with IS, five CNS diseases were selected as examples for further investigation, including Alzheimer's disease (AD, associated_disease_id: C0002395), epilepsy (associated_disease_id: C0014544), major depressive disorder (MDD, associated_disease_id: C1269683), amyotrophic lateral sclerosis (ALS, associated_disease_id: C0002736), and dementia (associated_disease_id: C0497327).

These genes were combined and sorted into different categories. Genes of IS and IS shared genes of AD, epilepsy, MDD, ALS, and dementia were designated as 1, 2, 3, 4, 5, and 6, respectively.

PPI relationships among disease genes of the abovementioned IS and its associated five CNS diseases were attained from STRING [26]. IS-related DDA network was created with the application of Cytoscape (Version 3.8.0).

### 2.3. IS-Related Gene Collection and Integration

We manually searched and collected IS-associated genes from public databases, i.e., DisGeNET, Malacards, and OMIM, the different gene symbols were normalized, and duplicated genes were further removed to get three-database-based IS-related genes. Then, these genes were integrated with genes of index disease (id: C0948008) to get the final genes of IS.

### 2.4. NBP Regulation on IS-Related DDA Network

NBP potential target genes were integrated into IS DDA network to get the NBP regulation network. The common genes of NBP and diseases may be the potential targets of NBP to show its modulation effects on diseases, i.e., IS, AD, epilepsy, MDD, ALS, and dementia. In order to indicate and visualize the gene-disease association (GDA) among genes and diseases, the DisGeNET Disease Specificity Index (DSI, range 0.25 to 1) and the Disease Pleiotropy Index (DPI, range 0 to 1) were cited to evaluate each gene [27]. The common target genes were listed in the bar figure ranked by DSI and DPI. Finally, the NBP-regulated network was constructed with Cytoscape (Version 3.8.0). Target gene nodes were tagged with different colors according to certain gene-disease associations.

### 2.5. Validation of Potential NBP Targets with Molecular Docking

The potential target genes of NBP with DSI >0.5 were selected, which had much bigger specificity to a certain disease. 3D target protein structures were obtained from the RCSB PDB database, and the target protein structures were pretreated by the application of AutoDock Tools (Version 1.5.6) to generate PDBQT files for molecular docking. NBP .mol2 file was generated with ChemDraw and Chem3D software according to the structure in PubChem (PubChem CID: 61361). The file was further processed and prepared for molecular docking in AutoDock Vina software.

## 3. Results

### 3.1. NBP Potential Target Identification and Integration

There were a total of 146 potential NBP target genes retrieved and identified from PubMed references. In PharmMapper and DRAR-CPI, 15 potential or putative target genes were manually identified and selected. Another 17 potential target genes were retrieved from the SymMap database. After removing duplicated genes, 175 NBP potential target genes were identified for investigation in deep. The 175 genes were listed in Supplementary Table (Additional file 1).

### 3.2. IS-Related Gene Collection and Integration

DisGeNET, Malacards, and OMIM were the three sources to obtain IS-associated disease genes. There were 167 IS-related genes attained from DisGeNET, while 168 and 20 genes were obtained from Malacards and OMIM, respectively. These gene names were normalized into official gene symbols, and duplicated symbols were removed. Finally, 312 IS-related genes were obtained for subsequent study (Additional file 2).

### 3.3. IS-Related DDA Network

“Ischemic stroke” was used as index disease (Index-disease-id: C0948008), and a list of 393 genes was retrieved from DisGeNET (5^th^ Dec. 2019) (Additional file 3). These 393 genes were integrated with the abovementioned 312 genes obtained from three sources to get the final 94 IS-associated disease genes (Additional file 4).

**Figure 2:**
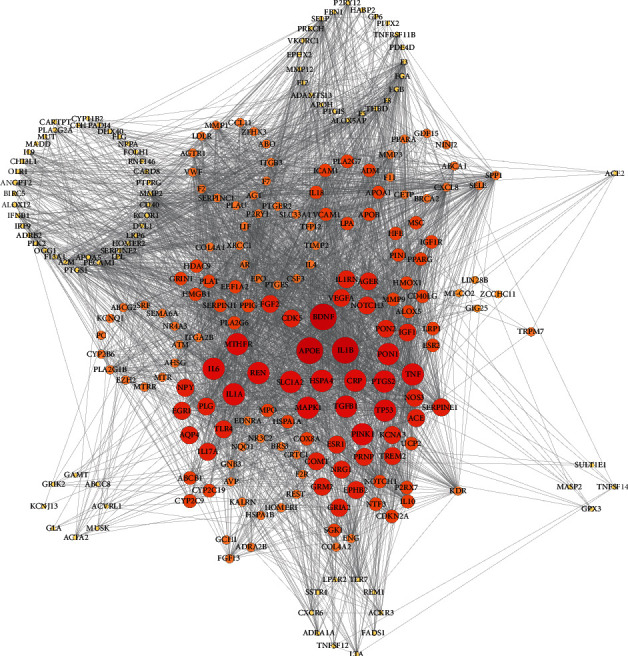
Ischemic stroke- (IS-) related disease-disease association (DDA) network. The network contained 241 disease gene nodes and 3433 edges. The node size and color were in proportion to the number of interacted nodes; the more nodes one node linked, the bigger size and deeper color it showed.

Shared gene lists of AD with IS were retrieved through DDAs (Index-disease-id: C0948008) in DisGeNET, and the same procession was conducted with epilepsy, MDD, ALS, and dementia to obtain their respective shared genes with IS. There were 189 shared genes of AD and IS, while the numbers for epilepsy, MDD, ALS, and dementia were 77, 75, 67, and 64, respectively. These shared genes were listed in Supplementary Table (Additional file 5). IS DDA network was constructed according to PPI relationships obtained from STRING. The network contained 241 disease gene nodes and 3433 edges. The node size and color were in proportion to the number of interacted nodes; the more nodes one node linked, the bigger size and deeper color it showed (Figure 2).

### 3.4. NBP Regulation on IS-Related DDA Network

**Figure 3:**
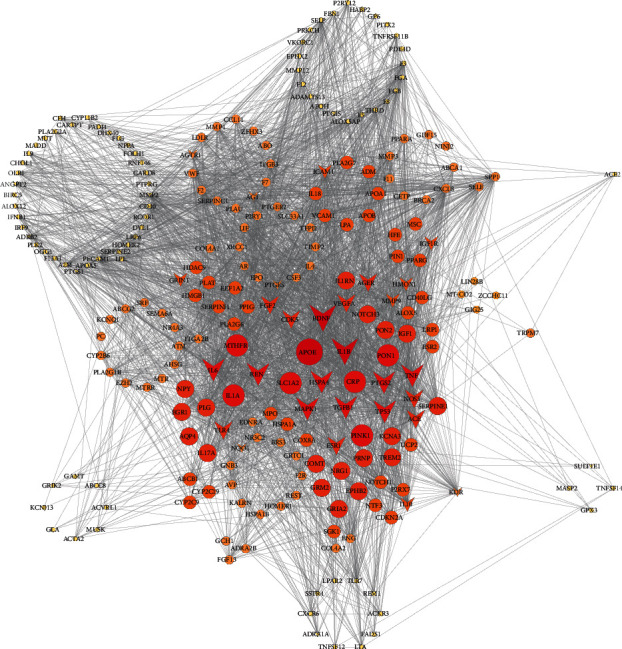
Butylphthalide (NBP) regulation on ischemic stroke- (IS-) related disease-disease association (DDA) network. The 175 potential target genes of NBP were integrated into IS DDA network, and the NBP regulation network was created, in which 36 common genes of NBP and diseases were identified and these 36 nodes were marked as V shape. The node size and color were in proportion to the number of interacted nodes; the more nodes one node linked, the bigger size and deeper color it showed.

The 175 potential target genes of NBP were integrated into IS DDAs network, and the NBP regulation network was created, in which 36 common genes of NBP and diseases were identified and these 36 nodes were marked as V shape (Figure 3).

**Figure 4:**
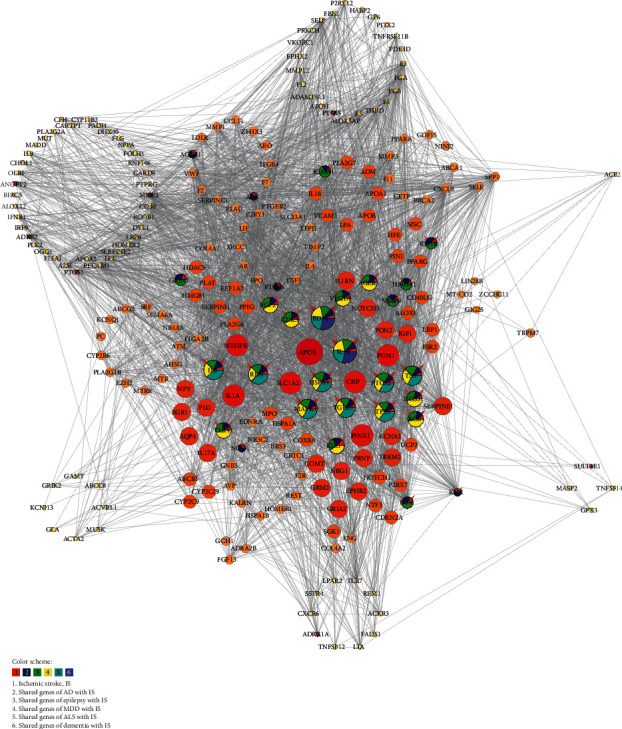
Pie chart of butylphthalide (NBP) with its regulation on ischemic stroke- (IS-) related disease-disease association (DDA) network. Node pie chart color represents different types of disease genes, i.e., genes of IS (in red), shared genes of AD with IS (in blue), shared genes of epilepsy with IS (in green), shared genes of MDD with IS (in yellow), shared genes of ALS with IS (in cyan), and shared genes of dementia with IS (in purple), respectively. The pie chart was generated in Cytoscape 3.8.0.

As each node may be associated with different diseases, the 36 nodes were marked with different colors according to attributions (Figure 4). In node pie chart color scheme, 1, 2, 3, 4, 5, and 6 represent IS, shared genes of AD with IS, shared genes of epilepsy with IS, shared genes of MDD with IS, shared genes of ALS with IS, and shared genes of dementia with IS, respectively.

With network analysis, the associated relationships between the target gene and IS-related disease were predicted; i.e., GRIN1 is associated with AD and epilepsy, PTGES is related to AD and ALS, ADRA1A has a link with depression, CDK5 connects with AD, depression, and ALS, while SULT1E1 may have an association with ALS. These inferred associations could be the underlying basis of repositioning of NBP for IS-associated CNS diseases, and further pieces of evidence were obtained in the discussion section.

**Figure 5:**
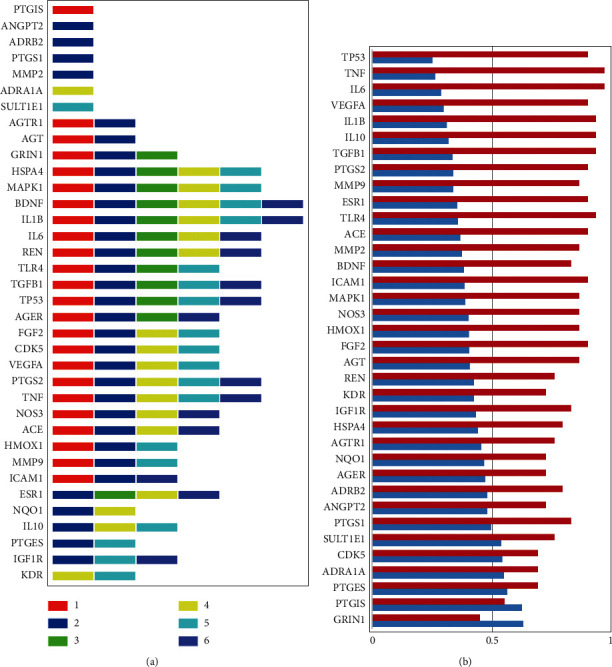
The 36 potential butylphthalide (NBP) target genes in the regulation network of NBP. (a) Gene associations with diseases. 1, 2, 3, 4, 5, and 6 represent IS, shared genes of AD with IS, shared genes of epilepsy with IS, shared genes of MDD with IS, shared genes of ALS with IS, and shared genes of dementia with IS, respectively. The corresponding color was red, blue, green, yellow, cyan, and purple for 1, 2, 3, 4, 5, and 6. (b) DSI and DPI properties of the 36 potential NBP target genes.

The corresponding color was red, blue, green, yellow, cyan, and purple for 1, 2, 3, 4, 5, and 6 (Figure 5(a)). DSI and DPI properties of the 36 node genes were also visualized, in which the 6 NBP potential target genes, i.e., GRIN1, PTGIS, PTGES, ADRA1A, CDK5, and SULT1E1, showed DSI >0.5 (Figure 5(b)). DSI, which is short for disease specificity index in DisGeNET, shows the association degree of a certain gene with several or fewer diseases. And DSI >0.5 meant that genes were more related to IS. All of the 6 genes had DPI values of more than 0.4. NBP, as the approved drug by NMPA for IS treatment may show potential regulation effects on several IS-associated CNS diseases through modulation of certain critical DDAs underlying shared genes.

### 3.5. Validation of Potential NBP Targets with Molecular Docking

Six potential targets of NBP, i.e., GRIN1, PTGIS, PTGES, ADRA1A, CDK5, and SULT1E1, were selected for further validation with *in silico* molecular docking. Protein structures were obtained from the RCSB PDB database: GRIN1 (PDB: 6IRA, Electron microscopy, 4.50 Å resolution), PTGIS (PDB: 3B6H, X-ray diffraction, 1.62 Å resolution), PTGES (PDB: 3DWW, Electron microscopy, 3.50 Å resolution), CDK5 (PDB: 1UNL, X-ray diffraction, 2.20 Å resolution), and SULT1E1 (PDB: 1G3M, X-ray diffraction, 1.70 Å resolution) while the HUMAN-Homology model ADRA1A was achieved from SWISS-MODEL database (P35348, https://swissmodel.expasy.org/repository/uniprot/P35348?template=4ej4.1.A&range=22-336).

A docking score was applied to evaluate the binding degree between drug and target. Three levels of the score were used to evaluate the binding activity. When the docking score was less than “−5”, this meant that the drug had a certain binding affinity with the target protein. When the docking score was less than “−7”, this showed that the drug had good binding activity. When the docking score was less than “−9”, this indicated that the drug had a strong binding ability [28].

**Figure 6:**
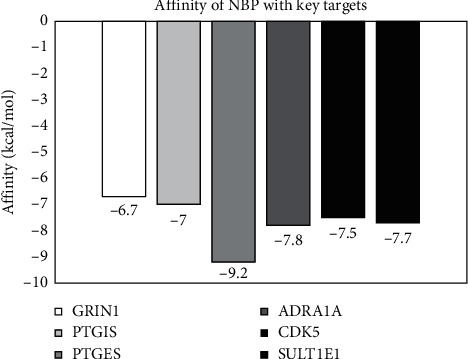
Binding affinity of butylphthalide (NBP) with the six key targets. NBP had a strong binding activity with PTGES (affinity = −9.2 kcal/mol), certain binding ability with GRIN1 (affinity = −6.7 kcal/mol), and good binding ability with PTGIS, ADRA1A, CDK5, and SULT1E1 (affinity = −7.0, −7.8, −7.5, and −7.7 kcal/mol, respectively).

As shown in Figure 6, NBP had a strong binding activity with PTGES (affinity = −9.2 kcal/mol), certain binding ability with GRIN1 (affinity = −6.7 kcal/mol), and good binding ability with PTGIS, ADRA1A, CDK5, and SULT1E1 (affinity = −7.0, −7.8, −7.5 and −7.7 kcal/mol, respectively).

**Figure 7:**
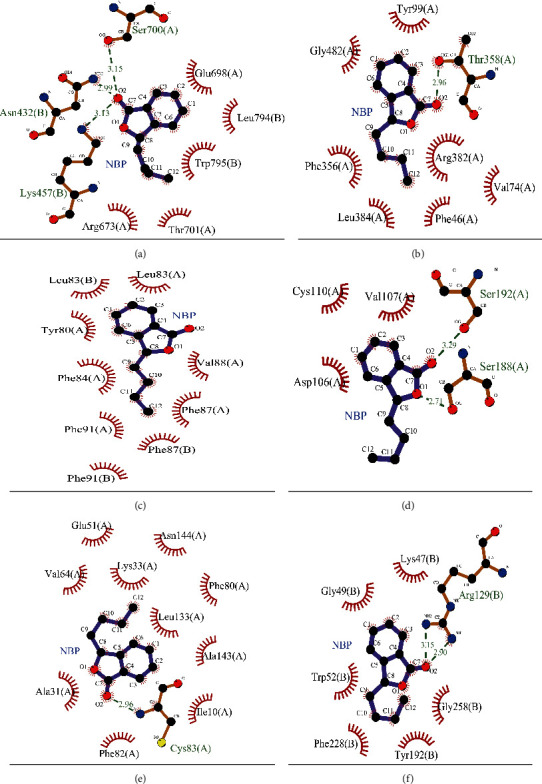
The binding modes of NBP with potential target proteins. Binding modes were analyzed by LigPlot+ (Version 2.2). The green dashed lines stand for hydrogen bonds (H-bonds), while the green numbers on lines stand for distances (Å). The purple bonds stand for ligand bonds, while the brown color stands for nonligand bonds. And the brick red color represents hydrophobic “bonds.” For atoms, the blue colors stand for nitrogen atoms, the red color stands for oxygen atoms, the yellow color was for sulfur atoms, and the black color represents carbon atoms. (a) NBP_GRIN1. (b) NBP_PTGIS. (c) NBP_PTGES. (d) NBP_ADRA1A. (e) NBP_CDK5. (f) NBP_SULT1E1.

The binding modes of NBP with potential target proteins were analyzed by LigPlot+ (Version 2.2). As depicted in Figure 7, GRIN1 had three hydrogen bonds (H-bonds) with NBP (Chain A: Ser 700; Chain B: Asn 432, Lys 457) together with five hydrophobic interactions (Chain A: Arg 673, Glu 698, Thr 701; Chain B: Leu 794, Trp 795). Intriguingly, six conformation structures of NBP possessed a certain binding affinity with GRIN1 or the interface of GRIN1 and GRIN2A in the agonist binding domain (ABD) of a heterotetramer NMDA receptor (details shown in Additional file 6). NBP showed one H-bond with PTGIS (Chain A: Thr 358) and seven hydrophobic interactions (Chain A: Phe 46, Val 74, Tyr 99, Phe 356, Arg 382, Leu 384, and Gly 482). For PTGES, the main interactions between NBP and protein were hydrophobic “bonds” (Chain A: Tyr 80, Leu 83, Phe 84, Phe 87, Val 88 and Phe 91; Chain B: Leu 83, Phe 87 and Phe 91). As for ADRA1A, NBP showed two H-bonds, i.e., Chain A: Ser 188 and Ser 192. Three hydrophobic interactions (Chain A: Asp 106, Val 107, and Cys 110) contributed to the interactions between NBP and ADRA1A. What's more, NBP had one H-bond (Chain A: Cys 83) with CDK5 and 10 hydrophobic interactions (Chain A: Ile 10, Ala 31, Lys 33, Glu 51, Val 64, Phe 80, Phe 82, Leu 133, Ala 143, and Asn 144). And for SULT1E1, NBP possessed two H-bonds and six hydrophobic “bonds” (H-bonds, Chain B: Arg 129; hydrophobic interactions, Chain B: Lys 47, Gly 49, Trp 52, Tyr 192, Phe 228, and Gly 258).

## 4. Discussion

CNS diseases are complex and polygenic diseases lacking clinical effective drugs for treatment. Drug repurposing/repositioning is one of the feasible and attractive strategies to “recycle” the currently approved drugs. Based on the DDAs, diseases sharing common disease modules, i.e., the underlying disease-associated gene clusters, may have similar symptoms and high comorbidity [3]. IS, as a complex multifactorial disorder to cause brain tissue ischemia and injury, may induce or accompany comorbidities. Human symptoms-disease network [29] showed the IS-related CNS diseases including AD, dementia, epilepsy, glioblastoma/glioma, motor neuron disease, meningioma, listeriosis, and MDD, with Symptom Similarity Score ≥0.2. Our study set IS-related CNS diseases (i.e., AD, dementia, epilepsy, ALS, and MDD) as examples to elucidate the potential effects of NMPA-approved anti-IS drug NBP on these 5 diseases. NBP showed pleiotropic neuroprotective effects, which may be the underlying molecular mechanisms for its repurposing for the treatment of those five diseases. DisGeNET, Malacards, and OMIM were open-access and high-quality public online disease databases, which contributed to the credibility of the disease-related gene acquisition. STRING was used to retrieve PPI relationships, while network pharmacology methods were applied to construct the DDA network and drug-disease network. With network analysis, critical information contained in networks was extracted, such as the common genes of DDAs, which could be the underlying molecular basis of NBP repositioning.

Through the strategy proposed in this study, six potential targets of NBP, i.e., GRIN1, PTGIS, PTGES, ADRA1A, CDK5, and SULT1E1, were selected for further validation with *in silico* molecular docking. The pieces of evidence were obtained from references to support these associations among targets and diseases as follows.

### 4.1. GRIN1

GRIN1 is an indispensable component of the heterotetramer of NMDA receptor complexes and plays a critical role in the plasticity of synapses. Our results indicated that it had associations with IS, AD, and epilepsy. The learning and memory-related GRIN1 gene was found downregulated in ovariectomized AD rats [30]. Studies reported that GRIN1 was one of the top 25 core genes of the AD network [31]. Another study showed GRIN1 as one of the target genes of the approved anti-AD drug memantine [32].

GRIN1 was also one of the top candidate genes for epileptogenesis [33]. The whole-exome sequencing analysis of patients found that GRIN1 mutations caused seizures and movement disorders [34]. GRIN1 mutation associated with intellectual disability alters NMDA receptor trafficking and function [35]. 70%–80% of epilepsy is attributed to genetic factors, the GRIN1 gene encodes the NMDA receptor GluN1 subunit, and the NMDA receptor has an important role in epilepsy [36].

### 4.2. PTGES

Molecular docking results indicated that NBP had a strong binding activity with PTGES (affinity = −9.2 kcal/mol). As we know, PTGES encodes prostaglandin E synthase, which catalyzes the glutathione-dependent oxidoreduction of prostaglandin endoperoxide H_2_ (PGH_2_) to prostaglandin E_2_ (PGE_2_) in response to inflammatory stimuli. Studies showed that microsomal prostaglandin E synthase 1 (mPGES-1) expression was significantly elevated in middle frontal gyrus tissues of AD patients compared with controls, and the proinflammatory PGE_2_ [37] was found to be elevated in the cerebrospinal fluid early in AD [38]. However, the effect of PGE_2_ depends on its concentration and to which receptor it binds [39]. Reports indicated that mPGES-1 was induced in A*β*-mediated neuronal cell death, and PGE_2_ was an important factor involved in A*β*-induced neurotoxicity [40]. mPGES-1 may play a role in AD pathology [41].

ALS is a progressive neurodegenerative disorder characterized by the selective death of motor neurons. PGE_2_ was elevated in the serum and CSF of ALS patients, and mPGES-1 inhibition may reduce microglial activation and motor neuron loss [42]. Thus, mPGES-1 in motor neurons may play a role in the pathogenesis of ALS [43]. Another work reported that the concurrent inhibition of mPGES-1 and free radicals would be a promising strategy to combat neurodegeneration in ALS [44].

### 4.3. ADRA1A

As for depression disorder, tricyclic antidepressant (TCA) drugs can also target *α*_1_-adrenergic receptor (AR) as antagonists, which may be the underlying mechanisms for therapy [45]. Further investigations into the subtypes of *α*_1_-AR indicated that *α*_1A_-AR may be a useful therapeutic target for the treatment of depression [46]. What's more, norepinephrine (NE) plays an important role in behavior and cognition and involves depression regulation [47]. While *α*_1A_-AR was associated with antidepressant-like effects, chronic *α*_1B_-AR stimulation showed prodepressant [48]. Deeper investigations deserve to be designed and conducted to clarify the involvement role of *α*_1A_-AR against depression.

### 4.4. CDK5

CKD5 is a versatile kinase showing a pivotal role in modulating the function of postmitotic neurons in the developing CNS; the dysregulation of CDK5 is involved in the pathology of neurodegenerative diseases, such as AD, ALS, and IS [49, 50]. CDK5 deregulation contributes to the pathogenesis of AD through inducing deposition of A*β* in senile plaques and intracellular accumulations of hyperphosphorylated tau. The balance of CDK5/P35 and CDK5/P25 is crucial for neuronal migration and differentiation, neurite outgrowth, synaptic growth, and functions. Elevated and sustained Cdk5/p25 activity consequently leads to DNA damage, cell death, and neurodegeneration, whereas the Cdk5/p35 complex is neuroprotective [51, 52]. Thus, CDK5 could be a potential therapeutic target for AD [53–55].

As suppression of overactivated or deregulated CDK5 offers neuroprotection and prevention of motor neuron loss. ALS, the progressive and fatal neurodegenerative disorder leading to muscle atrophy, loss of movement, and eventually death, could gain benefits through inhibition of CDK5 [51, 56–58].

CDK5 was reported to have an association with depression, but the underlying mechanisms may be complex. CDK5 in the dentate gyrus was found to participate in the process of depressive-like behavior in rats. Inhibition of CDK5 in the dentate gyrus contributed to the antidepressant actions and ameliorated the depressive-like symptoms [59]. CDK5 loss of function in the ventral tegmental area (VTA) induced anxiety- and depressive-like behaviors in mouse models which were associated with inhibited tyrosine hydroxylase phosphorylation at Ser31 and Ser 40 [60]. In an MDD mouse model, studies indicated that CDK5-mediated phosphorylation of Sirt2 at Ser 368 and 372 in amygdala accounting for social defeat stress induced depressive-like behavior. Thus, inhibition of CDK5-dependent Sirt2 phosphorylation at Ser 368 and 372 may be a promising strategy for antidepression therapy development [61]. Another study showed Cdk5 in the nucleus accumbens as a critical contributor to depressive-like behaviors in Huntington's disease mouse models [62].

### 4.5. SULT1E1

Estrogen sulfotransferase (EST, encoded by SULT1E1) catalyzes the sulfoconjugation and inactivation of estrogen. Reports showed that estrogen exerts neuroprotective and anti-inflammatory effects in ALS [63, 64]. As EST mediates the metabolic deactivation of estrogen [65], drugs targeting EST may have potential regulation effects against ALS.

In summary, our study proposed a network pharmacology and molecular docking integrated strategy to predict several critical targets of NBP with drug repurposing potential; *in silico* molecular docking and literature validations were conducted to support these findings. However, more works in deep are warranted to design and investigate the associations between NBP and AD, epilepsy, ALS, and depression.

## 5. Conclusions

NBP, an NMPA-approved drug for IS treatment, shows multitarget and pleiotropic effects. IS has DDAs with several CNS diseases, including AD, ALS, epilepsy, MDD, and dementia. Network pharmacology could facilitate the construction of NBP-regulated disease networks to explore the underlying molecular mechanisms of potential effects. 36 potential target genes of NBP were identified for IS-associated CNS diseases, and 6 of them, i.e., GRIN1, PTGIS, PTGES, ADRA1A, CDK5, and SULT1E1 were validated by the application of *in silico* molecular docking. All of the six targets indicated certain to good binding affinity with NBP. References were retrieved to further validate the association between target genes and IS-related CNS diseases. Based on network analysis and validations, NBP may have the potential to be repositioned for AD, epilepsy, ALS, and depression treatments.

In summary, our study has proposed a drug-target-disease integrating method to predict the drug repurposing potentials to other associated diseases by the application of network pharmacology and molecular docking, which could be an attractive alternative to facilitate the development of CNS disease therapies. As this drug repositioning strategy is based on databases, *in silico* molecular docking, and published references, further in-deep investigations are warranted to be carefully designed and conducted.

## Data Availability

All data are available within the article and are shown in figures and supplemental files.

## Supplementary Materials

Supplementary Materials Additional file 1: the 175 NBP genes. Additional file 2: 312 IS-related genes obtained from databases of Malacards, DisGeNET, and OMIM. Additional file 3: genes of ischemic stroke collected from DisGeNET database (Index_disease_id: C0948008). Additional file 4: final 94 ischemic stroke genes after gene symbols are normalized and duplicated genes are removed. Additional file 5: shared genes of AD, epilepsy, MDD, ALS, and dementia with IS, respectively. Additional file 6: six conformations of GRIN1 in molecular docking with NBP.
