# Supplementary material for: A Study to Decipher the Potential Effects of Butylphthalide against Central Nervous System Diseases Based on Network Pharmacology and Molecular Docking Integration Strategy
Source: Evid Based Complement Alternat Med. 2021 May 4;2021:6694698. doi: 10.1155/2021/6694698 (PMC8116153; doi:10.1155/2021/6694698)
Supplement: Supplementary Materials — Additional file 1: the 175 NBP genes. Additional file 2: 312 IS-related genes obtained from databases of Malacards, DisGeNET, and OMIM. Additional file 3: genes of ischemic stroke collected from DisGeNET database (Index_disease_id: C0948008). Additional file 4: final 94 ischemic stroke genes after gene symbols are normalized and duplicated genes are removed. Additional file 5: shared genes of AD, epilepsy, MDD, ALS, and dementia with IS, respectively. Additional file 6: six conformations of GRIN1 in molecular docking with NBP. [file 6694698.f1.pdf]

Additional file1 The 175 NBP genes

target genes	sources
ALB	PharmMapper_and_DRAR-CPI
ATIC	PharmMapper_and_DRAR-CPI
BCHE	PharmMapper_and_DRAR-CPI
BMP7	PharmMapper_and_DRAR-CPI
CD1A	PharmMapper_and_DRAR-CPI
CSF2RB	PharmMapper_and_DRAR-CPI
ESR1	PharmMapper_and_DRAR-CPI
FABP7	PharmMapper_and_DRAR-CPI
GC	PharmMapper_and_DRAR-CPI
GSTT2B	PharmMapper_and_DRAR-CPI
HDAC7	PharmMapper_and_DRAR-CPI
IGF1R	PharmMapper_and_DRAR-CPI
NR1I3	PharmMapper_and_DRAR-CPI
ODC1	PharmMapper_and_DRAR-CPI
SULT1E1	PharmMapper_and_DRAR-CPI
CASP3	PubMed
VEGFA	PubMed
AKT1	PubMed
SOD1	PubMed
BCL2	PubMed
RELA	PubMed
TNF	PubMed
HMOX1	PubMed
GFAP	PubMed
NFE2L2	PubMed
IL1B	PubMed
MAPK1	PubMed
BAX	PubMed
BDNF	PubMed
DLG4	PubMed
CAT	PubMed
CLDN5	PubMed
GSK3B	PubMed
IL6	PubMed
MAPK14	PubMed
MAPK3	PubMed
MAPK8	PubMed
SYP	PubMed
CTNNB1	PubMed
FGF2	PubMed
HIF1A	PubMed
HSPA5	PubMed
PIK3CA	PubMed
ATF6	PubMed
CASP12	PubMed
CASP9	PubMed
CHAT	PubMed
CREB1	PubMed
DDIT3	PubMed
EDN1	PubMed

remove duplicated genes (175 genes left)
ACE
ACHE
ADRA1A
ADRA2A
ADRB1
ADRB2
AGER
AGT
AGTR1
AKT1
ALB
ANGPT1
ANGPT2
APP
ARG1
ASCL1
ATF4
ATF6
ATIC
BACE1
BAX
BCHE
BCL2
BCL2L2
BDNF
BECN1
BMP7
BNIP3
CAMK2G
CAPN1
CASP12
CASP3
CASP9
CAT
CCL2
CCND1
CD1A
CD34
CDH2
CDK5
CHAT
CHRM1
CLDN5
CREB1
CSF2RB
CTNNB1
CTNND1
CYCS
DDIT3
DLG4

MAP1LC3A	PubMed
MAPT	PubMed
NFKBIA	PubMed
NGF	PubMed
NQO1	PubMed
PRKCA	PubMed
TGFB1	PubMed
TJP1	PubMed
XBP1	PubMed
ANGPT1	PubMed
APP	PubMed
ATF4	PubMed
BECN1	PubMed
CAMK2G	PubMed
CAPN1	PubMed
GAP43	PubMed
GJA1	PubMed
GPX1	PubMed
GRIN2B	PubMed
ICAM1	PubMed
ITGAM	PubMed
MMP9	PubMed
MTOR	PubMed
NOS3	PubMed
PDIA6	PubMed
RHOA	PubMed
RTN4	PubMed
SHH	PubMed
SNCA	PubMed
TLR4	PubMed
TXN	PubMed
ACE	PubMed
ACHE	PubMed
AGER	PubMed
AGT	PubMed
AGTR1	PubMed
ANGPT2	PubMed
ARG1	PubMed
ASCL1	PubMed
BACE1	PubMed
BCL2L2	PubMed
BNIP3	PubMed
CCL2	PubMed
CCND1	PubMed
CD34	PubMed
CDH2	PubMed
CDK5	PubMed
CTNND1	PubMed
CYCS	PubMed
DNM1L	PubMed
DUSP2	PubMed
ECE1	PubMed
EGF	PubMed
EIF2AK3	PubMed

DNM1L
DRD2
DUSP2
ECE1
EDN1
EGF
EIF2AK3
EIF2S1
ELK1
ERN1
ESR1
FABP7
FASLG
FGF2
FGFR2
FIS1
FOS
FOXP3
GAD1
GAD2
GAP43
GAPDH
GC
GFAP
GJA1
GLI1
GPX1
GRIN1
GRIN2B
GSK3B
GSR
GSS
GSTT2B
HDAC7
HGF
HIF1A
HMOX1
HSPA4
HSPA5
ICAM1
IFNG
IGF1R
IL10
IL1B
IL6
ITGAM
JUN
KDR
KEAP1
MAOA
MAOB
MAP1LC3A
MAPK1
MAPK14

EIF2S1	PubMed
ELK1	PubMed
ERN1	PubMed
FASLG	PubMed
FGFR2	PubMed
FIS1	PubMed
FOS	PubMed
FOXP3	PubMed
GAD1	PubMed
GAD2	PubMed
GAPDH	PubMed
GLI1	PubMed
GRIN1	PubMed
GSR	PubMed
GSS	PubMed
HGF	PubMed
HSPA4	PubMed
IFNG	PubMed
IL10	PubMed
JUN	PubMed
KDR	PubMed
KEAP1	PubMed
MFN1	PubMed
MFN2	PubMed
MMP2	PubMed
NCF2	PubMed
NOS2	PubMed
NOX2	PubMed
NTRK2	PubMed
OCLN	PubMed
OPA1	PubMed
PDE2A	PubMed
PGAM5	PubMed
PLA2G4A	PubMed
PPARGC1A	PubMed
PRKAA1	PubMed
PRKCE	PubMed
PRKCG	PubMed
PTCH1	PubMed
PTGES	PubMed
PTGIS	PubMed
PTGS2	PubMed
REN	PubMed
RORA	PubMed
RTN4R	PubMed
S100B	PubMed
SIRT1	PubMed
SLC17A7	PubMed
SLC18A2	PubMed
SMO	PubMed
SOD2	PubMed
TBXAS1	PubMed
TEK	PubMed
TNFSF10	PubMed

MAPK3
MAPK8
MAPT
MFN1
MFN2
MMP2
MMP9
MTOR
NCF2
NFE2L2
NFKBIA
NGF
NOS2
NOS3
NOX2
NQO1
NR1I3
NTRK2
OCLN
ODC1
OPA1
PDE2A
PDIA6
PGAM5
PIK3CA
PLA2G4A
PPARGC1A
PRKAA1
PRKCA
PRKCE
PRKCG
PTCH1
PTGES
PTGIS
PTGS1
PTGS2
RELA
REN
RHOA
RORA
RTN4
RTN4R
RXRA
S100B
SCN5A
SHH
SIRT1
SLC17A7
SLC18A2
SLC6A2
SLC6A3
SLC6A4
SMO
SNCA

TP53	PubMed
TUBB3	PubMed
TXNIP	PubMed
ADRA1A	SymMap
ADRA2A	SymMap
ADRB1	SymMap
ADRB2	SymMap
CHRM1	SymMap
DRD2	SymMap
FGF2	SymMap
MAOA	SymMap
MAOB	SymMap
PTGS1	SymMap
PTGS2	SymMap
RXRA	SymMap
SCN5A	SymMap
SLC6A2	SymMap
SLC6A3	SymMap
SLC6A4	SymMap
VEGFA	SymMap

SOD1
SOD2
SULT1E1
SYP
TBXAS1
TEK
TGFB1
TJP1
TLR4
TNF
TNFSF10
TP53
TUBB3
TXN
TXNIP
VEGFA
XBP1

Additional file2 312 IS-related genes (Malacards_DisGeNET_OMIM)

Symbol	source
F2	Malacards
F5	Malacards
PRKCH	Malacards
ALOX5AP	Malacards
NOS3	Malacards
NOTCH3	Malacards
LDLR	Malacards
ACSL4	Malacards
FBN1	Malacards
MT-TL1	Malacards
PIK3CA	Malacards
CDKN2B-AS1	Malacards
MALAT1	Malacards
MIAT	Malacards
ACE	Malacards
PLAT	Malacards
PDE4D	Malacards
PTGIS	Malacards
APOH	Malacards
MTHFR	Malacards
SERPINC1	Malacards
KCNB1	Malacards
SERPINE1	Malacards
ODC1	Malacards
SELP	Malacards
F3	Malacards
KMO	Malacards
HSPA1A	Malacards
KYNU	Malacards
VWF	Malacards
THBD	Malacards
SULT1A3	Malacards
CRP	Malacards
ADORA1	Malacards
GRIN2A	Malacards
APOE	Malacards
CASP3	Malacards
S100B	Malacards
SLC1A1	Malacards
ADCY10	Malacards
BAD	Malacards
PTK2B	Malacards
MAP2	Malacards
ENO2	Malacards
IL1B	Malacards
NCAM1	Malacards
DRD1	Malacards
GCKR	Malacards
KRT18	Malacards
HSPA8	Malacards

312 Symbols (duplicated genes removal)	count
ALOX5AP	3
F2	3
F5	3
NOS3	3
PDE4D	3
PRKCH	3
ACE	2
ADORA1	2
APOA1	2
APOE	2
BAD	2
BDNF	2
CASP3	2
CCL2	2
CD163	2
CREB1	2
CYCS	2
EDN1	2
FGA	2
FOS	2
HBA1	2
IL1B	2
ITGA2B	2
LPA	2
MMP9	2
MTHFR	2
NOS2	2
NOTCH3	2
PARP1	2
PLA2G7	2
PLAT	2
S100B	2
SELE	2
SERPINC1	2
SERPINE1	2
TNF	2
VWF	2
ABCC11	1
ABO	1
ACRDYS2	1
ACSL4	1
ADAMTS1	1
ADAMTS13	1
ADAMTS18	1
ADCY10	1
ADD3	1
ADH1B	1
ADM	1
AGER	1
AGT	1

BDNF	Malacards
CCL2	Malacards
CD40LG	Malacards
NES	Malacards
BCO1	Malacards
ADAMTS1	Malacards
ADM	Malacards
AQP4	Malacards
CNTF	Malacards
EPHA3	Malacards
GDNF	Malacards
GFAP	Malacards
EDN1	Malacards
NOS2	Malacards
PARP1	Malacards
PLG	Malacards
CTF1	Malacards
PROC	Malacards
CKB	Malacards
HSPA4	Malacards
MBP	Malacards
SRPK2	Malacards
LCN2	Malacards
CA4	Malacards
FGB	Malacards
SMYD3	Malacards
APOBEC3G	Malacards
CA1	Malacards
CNOT3	Malacards
KLKB1	Malacards
FOS	Malacards
GRIN2B	Malacards
GRIN1	Malacards
SLC1A3	Malacards
PF4	Malacards
TH	Malacards
KIAA1147	Malacards
SERPIND1	Malacards
LPA	Malacards
AIF1	Malacards
AGTR1	Malacards
ENTPD1	Malacards
PPBP	Malacards
BMP7	Malacards
COG2	Malacards
PROZ	Malacards
GP6	Malacards
REN	Malacards
EPHX2	Malacards
FGA	Malacards
PLA2G7	Malacards
P2RY12	Malacards
APOB	Malacards
AKT1	Malacards

AGTR1	1
AGTR2	1
AIF1	1
AKT1	1
ALB	1
ALOX5	1
ANK2	1
APOB	1
APOBEC3G	1
APOH	1
AQP4	1
ASAH1	1
ASIC1	1
ATF3	1
BBC3	1
BCL2	1
BCO1	1
BLVRA	1
BMP7	1
C3	1
CA1	1
CA4	1
CADASIL	1
CASIL	1
CASP9	1
CASZ1	1
CAT	1
CCL11	1
CCL3	1
CCL4	1
CCL7	1
CCR4	1
CD40LG	1
CDK5	1
CDK6	1
CDKN2B-AS1	1
CEBPB	1
CHRM1	1
CKB	1
CNOT3	1
CNTF	1
COG2	1
CRP	1
CSF2	1
CST3	1
CTF1	1
CXCL10	1
CXCL2	1
CXCR2	1
CXCR3	1
CYBB	1
DCX	1
DIABLO	1
DPDE3	1

TSPAN33	Malacards	DRD1	1
TLR4	Malacards	DYNLL1	1
MROS	Malacards	EDNRA	1
SLC1A2	Malacards	EGR1	1
NGB	Malacards	ENO2	1
TBXA2R	Malacards	ENTPD1	1
ASAH1	Malacards	EPHA3	1
HMGCR	Malacards	EPHX2	1
PON1	Malacards	F10	1
CREB1	Malacards	F12	1
F7	Malacards	F3	1
CYCS	Malacards	F7	1
PON2	Malacards	F8	1
RTN4	Malacards	F9	1
SERPINF2	Malacards	FAM107B	1
HBA1	Malacards	FASLG	1
NFE2L2	Malacards	FBN1	1
CST3	Malacards	FGB	1
HMGB1	Malacards	FGF2	1
AGTR2	Malacards	FGF21	1
DCX	Malacards	FLAP	1
ADAMTS13	Malacards	FOXF2	1
PVALB	Malacards	GABRA6	1
MMP9	Malacards	GCKR	1
APOA1	Malacards	GDNF	1
SLC39A2	Malacards	GFAP	1
MMP1	Malacards	GIPC1	1
TIMP1	Malacards	GJA1	1
SELE	Malacards	GP6	1
MMP8	Malacards	GPR17	1
CD163	Malacards	GPX1	1
HABP2	Malacards	GRIN1	1
TNFRSF11B	Malacards	GRIN2A	1
SPP1	Malacards	GRIN2B	1
TFPI	Malacards	GSTP1	1
TNF	Malacards	HABP2	1
TREM1	Malacards	HDAC9	1
ADAMTS18	Malacards	HIF1A	1
MYLK	Malacards	HMGB1	1
F8	Malacards	HMGCR	1
RNF213	Malacards	HMOX1	1
TNKS	Malacards	HP	1
HP	Malacards	HSPA1A	1
FAM107B	Malacards	HSPA4	1
F10	Malacards	HSPA8	1
CSF2	Malacards	HSPD1	1
IFNG	Malacards	HTR4	1
ITGA2B	Malacards	HTRA2	1
PGBD1	Malacards	ICAM1	1
RHNO1	Malacards	IFNA2	1
ABCC11	Malacards	IFNG	1
NT5C3A	Malacards	IGF1	1
BLVRA	Malacards	IL10RB	1
TAX1BP3	Malacards	IL11	1

F12	Malacards
GIPC1	Malacards
SLC22A12	Malacards
SLC13A2	Malacards
SLC13A5	Malacards
POMC	Malacards
SDC1	Malacards
LSS	Malacards
F9	Malacards
PDGFB	Malacards
SOD1	DisGeNet
SOD2	DisGeNet
CASP9	DisGeNet
CYCS	DisGeNet
NOS2	DisGeNet
C3	DisGeNet
HIF1A	DisGeNet
PLAT	DisGeNet
ADORA1	DisGeNet
BAD	DisGeNet
HTRA2	DisGeNet
ILK	DisGeNet
NDRG2	DisGeNet
IRAK1	DisGeNet
IRAK4	DisGeNet
VEGFA	DisGeNet
PLAU	DisGeNet
F2	DisGeNet
F5	DisGeNet
ICAM1	DisGeNet
IL1B	DisGeNet
IL6	DisGeNet
MMP9	DisGeNet
MTHFR	DisGeNet
ACE	DisGeNet
ALB	DisGeNet
CCL2	DisGeNet
IL1RN	DisGeNet
PTGS2	DisGeNet
TNF	DisGeNet
UCP2	DisGeNet
STAT3	DisGeNet
TP53	DisGeNet
ADD3	DisGeNet
ASIC1	DisGeNet
BBC3	DisGeNet
BDNF	DisGeNet
CASP3	DisGeNet
CAT	DisGeNet
CCL11	DisGeNet
CCL3	DisGeNet
CCL4	DisGeNet
CCR4	DisGeNet
CEBPB	DisGeNet

IL12RB1	1
IL17A	1
IL17RA	1
IL18	1
IL1A	1
IL1RN	1
IL22	1
IL6	1
IL6ST	1
ILK	1
IMF2	1
IRAK1	1
IRAK4	1
IRF1	1
ITGAV	1
ITGB3	1
ITPR1	1
ITPR2	1
JUN	1
JUNB	1
KCNB1	1
KCNK10	1
KCNK2	1
KCNK3	1
KIAA1147	1
KLKB1	1
KMO	1
KRT18	1
KYNU	1
LCN2	1
LDLR	1
LRCH1	1
LSS	1
MALAT1	1
MAP2	1
MAP2K1	1
MAP3K5	1
MAPK1	1
MAPK3	1
MBP	1
MIAT	1
MMP1	1
MMP12	1
MMP8	1
MPO	1
MRAS	1
MROS	1
MT2A	1
MT-TL1	1
MYLK	1
NCAM1	1
NDRG2	1
NES	1
NFE2L2	1

CHRM1	DisGeNet
CXCL10	DisGeNet
DIABLO	DisGeNet
DYNLL1	DisGeNet
EGR1	DisGeNet
GPX1	DisGeNet
IL10RB	DisGeNet
IL12RB1	DisGeNet
IL17A	DisGeNet
IL17RA	DisGeNet
IL18	DisGeNet
IL1A	DisGeNet
JUN	DisGeNet
MAP2K1	DisGeNet
MAPK1	DisGeNet
MAPK3	DisGeNet
MPO	DisGeNet
NFKB1	DisGeNet
NFKBIA	DisGeNet
PARP1	DisGeNet
RB1	DisGeNet
RELA	DisGeNet
SELE	DisGeNet
TNFRSF1A	DisGeNet
TNFRSF1B	DisGeNet
APOE	DisGeNet
CYBB	DisGeNet
EDN1	DisGeNet
FGF2	DisGeNet
GPR17	DisGeNet
SIRT1	DisGeNet
AGER	DisGeNet
BCL2	DisGeNet
CDK5	DisGeNet
CREB1	DisGeNet
GJA1	DisGeNet
ITPR1	DisGeNet
ITPR2	DisGeNet
KCNK10	DisGeNet
KCNK2	DisGeNet
LPA	DisGeNet
S100B	DisGeNet
SLC11A2	DisGeNet
SLC8A1	DisGeNet
NPY	DisGeNet
ALOX5AP	DisGeNet
NOS3	DisGeNet
PRKCH	DisGeNet
ADH1B	DisGeNet
VDR	DisGeNet
HBA1	DisGeNet
ABO	DisGeNet
ANK2	DisGeNet
APOA1	DisGeNet

NFKB1	1
NFKBIA	1
NGB	1
NGF	1
NKX2-5	1
NPY	1
NT5C3A	1
ODC1	1
P2RY12	1
PAOX	1
PDE3A	1
PDGFB	1
PF4	1
PGBD1	1
PIK3CA	1
PITX2	1
PKCL	1
PLAU	1
PLD1	1
PLG	1
PODXL	1
POMC	1
PON1	1
PON2	1
PPBP	1
PRKAA2	1
PRKCL	1
PROC	1
PROZ	1
PRPF8	1
PTGIS	1
PTGS2	1
PTK2B	1
PVALB	1
RB1	1
RELA	1
REN	1
RGS7	1
RHNO1	1
RNF213	1
RPRGL1	1
RPRGL2	1
RTN4	1
S100A5	1
SDC1	1
SELP	1
SERPIND1	1
SERPINF2	1
SERPINH1	1
SH2B3	1
SH3PXD2A	1
SIRT1	1
SLC11A2	1
SLC13A2	1

CASZ1	DisGeNet
CDK6	DisGeNet
CXCR3	DisGeNet
EDNRA	DisGeNet
FGA	DisGeNet
FOXF2	DisGeNet
HDAC9	DisGeNet
HTR4	DisGeNet
IRF1	DisGeNet
ITGA2B	DisGeNet
ITGAV	DisGeNet
ITGB3	DisGeNet
KCNK3	DisGeNet
LRCH1	DisGeNet
MMP12	DisGeNet
MT2A	DisGeNet
NKX2-5	DisGeNet
PAOX	DisGeNet
PDE3A	DisGeNet
PDE4D	DisGeNet
PITX2	DisGeNet
PLA2G7	DisGeNet
PRKAA2	DisGeNet
PRPF8	DisGeNet
RGS7	DisGeNet
SERPINC1	DisGeNet
SH2B3	DisGeNet
SH3PXD2A	DisGeNet
SMOX	DisGeNet
TBX3	DisGeNet
TGFB1	DisGeNet
TSPAN2	DisGeNet
VKORC1	DisGeNet
VWF	DisGeNet
WNT2B	DisGeNet
ZCCHC14	DisGeNet
ZFHX3	DisGeNet
IFNA2	DisGeNet
SERPINE1	DisGeNet
MAP3K5	DisGeNet
ALOX5	DisGeNet
ATF3	DisGeNet
SERPINH1	DisGeNet
FOS	DisGeNet
GABRA6	DisGeNet
CXCL2	DisGeNet
HSPD1	DisGeNet
IGF1	DisGeNet
IL6ST	DisGeNet
CXCR2	DisGeNet
IL11	DisGeNet
JUNB	DisGeNet
NGF	DisGeNet
PLD1	DisGeNet

SLC13A5	1
SLC1A1	1
SLC1A2	1
SLC1A3	1
SLC22A12	1
SLC39A2	1
SLC8A1	1
SMOX	1
SMYD3	1
SOD1	1
SOD2	1
SPHK2	1
SPP1	1
SRPK2	1
STAT3	1
STRK1	1
SULT1A3	1
TAX1BP3	1
TBX3	1
TBXA2R	1
TFPI	1
TGFB1	1
TH	1
THBD	1
THPH1	1
THPH2	1
TIMP1	1
TLR4	1
TNFAIP6	1
TNFRSF11B	1
TNFRSF1A	1
TNFRSF1B	1
TNKS	1
TP53	1
TREM1	1
TSPAN2	1
TSPAN33	1
UCP2	1
VCAM1	1
VDR	1
VEGFA	1
VKORC1	1
WNT2B	1
XDH	1
ZCCHC14	1
ZFHX3	1

S100A5	DisGeNet
CCL7	DisGeNet
TNFAIP6	DisGeNet
XDH	DisGeNet
FGF21	DisGeNet
IL22	DisGeNet
SPHK2	DisGeNet
GSTP1	DisGeNet
HMOX1	DisGeNet
AGT	DisGeNet
FASLG	DisGeNet
PODXL	DisGeNet
CD163	DisGeNet
MRAS	DisGeNet
VCAM1	DisGeNet
NOTCH3	OMIM
CADASIL	OMIM
CASIL	OMIM
IMF2	OMIM
PRKCH	OMIM
PKCL	OMIM
PRKCL	OMIM
NOS3	OMIM
F2	OMIM
THPH1	OMIM
RPRGL2	OMIM
F5	OMIM
THPH2	OMIM
RPRGL1	OMIM
PDE4D	OMIM
DPDE3	OMIM
STRK1	OMIM
ACRDYS2	OMIM
ALOX5AP	OMIM
FLAP	OMIM

Additional file3 Genes of Ischemic stroke, C0948008										
Index_disease	Index_di	Gene	Gene_id	UniProt	Gene_Ful	Protein_(	pLI	DSI_g	DPI_g	diseaseid1
Ischemic stroke	C0948008	XYLB	9942	O75191	xylulokiı	kinase; transfera		1	0.069	C0948008
Ischemic stroke	C0948008	ROPN1	54763	Q9HAT0	rhophilin associated tail			1	0.069	C0948008
Ischemic stroke	C0948008	METTL18	92342	O95568	methyltransferase like 18			1	0.069	C0948008
Ischemic stroke	C0948008	ACOT4	122970	Q8N9L9	acyl-CoA thioesterase 4			1	0.069	C0948008
Ischemic stroke	C0948008	TTC7B	145567	Q86TV6	tetratricopeptide repeat d			1	0.069	C0948008
Ischemic stroke	C0948008	NDUFC2	4718	O95298	NADH:ubi	oxidoreductase		0.928	0.138	C0948008
Ischemic stroke	C0948008	PDE4DIP	9659	Q5VU43	phosphodiesterase 4D inter			0.928	0.103	C0948008
Ischemic stroke	C0948008	KCNK17	89822	Q96T54	potassium two pore domain			0.886	0.172	C0948008
Ischemic stroke	C0948008	CILP2	148113	Q8IUL8	cartilage intermediate lay			0.886	0.172	C0948008
Ischemic stroke	C0948008	CYP2B7P	1556		cytochrome P450 family 2 s			0.857	0.172	C0948008
Ischemic stroke	C0948008	UBR3	130507	Q6ZT12	ubiquitin protein ligase E			0.857	0.103	C0948008
Ischemic stroke	C0948008	ARL6IP6	151188	Q8N6S5	ADP ribosylation factor li			0.857	0.172	C0948008
Ischemic stroke	C0948008	NINJ2	4815	Q9NZG7	ninjurin	cell adhesion mol		0.834	0.103	C0948008
Ischemic stroke	C0948008	SLC17A3	10786	O00476	solute ca	transporter		0.834	0.241	C0948008
Ischemic stroke	C0948008	RASIP1	54922	Q5U651	Ras interacting protein 1			0.834	0.207	C0948008
Ischemic stroke	C0948008	PTCSC3	1.01E+08		papillary thyroid carcinom			0.834	0.138	C0948008
Ischemic stroke	C0948008	IL1F10	84639	Q8WWZ1	interleukin 1 family membe			0.815	0.276	C0948008
Ischemic stroke	C0948008	NPAS4	266743	Q8IUM7	neuronal	nucleic acid bind		0.815	0.138	C0948008
Ischemic stroke	C0948008	SERPINA9	327657	Q86WD7	serpin fa	enzyme modulator		0.815	0.207	C0948008
Ischemic stroke	C0948008	RUVBL2	10856	Q9Y230	RuvB like AAA ATPase 2			0.799	0.276	C0948008
Ischemic stroke	C0948008	ASIC5	51802	Q9NY37	acid sens	transporter		0.799	0.345	C0948008
Ischemic stroke	C0948008	MIAT	440823		myocardial infarction asso			0.799	0.207	C0948008
Ischemic stroke	C0948008	LLGL2	3993	Q6P1M3	LLGL2, s	membrane traffic		0.785	0.172	C0948008
Ischemic stroke	C0948008	IRX4	50805	P78413	iroquois	nucleic acid bind		0.785	0.172	C0948008
Ischemic stroke	C0948008	AGXT2	64902	Q9BYV1	alanine--glyoxylate aminot			0.785	0.345	C0948008
Ischemic stroke	C0948008	HOMER2	9455	Q9NSB8	homer sca	signaling molecul		0.773	0.276	C0948008
Ischemic stroke	C0948008	HSPA12B	116835	Q96MM6	heat shock protein family			0.773	0.241	C0948008
Ischemic stroke	C0948008	CELSR1	9620	Q9NYQ6	cadherin	cell adhesion mol		0.762	0.345	C0948008
Ischemic stroke	C0948008	RCOR1	23186	Q9UKL0	REST corepressor 1			0.762	0.414	C0948008
Ischemic stroke	C0948008	MMD	23531	Q15546	monocyte	receptor		0.735	0.414	C0948008
Ischemic stroke	C0948008	RNF146	81847	Q9NTX7	ring finger protein 146			0.735	0.276	C0948008
Ischemic stroke	C0948008	F12	2161	P00748	coagulat	hydrolase; protea		0.727	0.379	C0948008
Ischemic stroke	C0948008	LGALS2	3957	P05162	galectin	cell adhesion mol		0.727	0.31	C0948008
Ischemic stroke	C0948008	NPR3	4883	P17342	natriuretic peptide recept			0.727	0.414	C0948008
Ischemic stroke	C0948008	CYP4F2	8529	P78329	cytochro	oxidoreductase		0.727	0.276	C0948008
Ischemic stroke	C0948008	DHX40	79665	Q8IX18	DEAH-box	nucleic acid bind		0.727	0.379	C0948008
Ischemic stroke	C0948008	CYP4A11	1579	Q02928	cytochro	oxidoreductase		0.72	0.31	C0948008
Ischemic stroke	C0948008	IL18RAP	8807	O95256	interleu	receptor		0.72	0.517	C0948008
Ischemic stroke	C0948008	KALRN	8997	O60229	kalirin	signaling molecul		0.72	0.207	C0948008
Ischemic stroke	C0948008	ALDH1L1	10840	O75891	aldehyde	oxidoreductase		0.72	0.414	C0948008
Ischemic stroke	C0948008	GALNT2	2590	Q10471	polypept	transferase		0.713	0.345	C0948008
Ischemic stroke	C0948008	ADAM11	4185	O75078	ADAM met	hydrolase; protea		0.713	0.414	C0948008
Ischemic stroke	C0948008	SPIDR	23514	Q14159	scaffold protein involved			0.713	0.448	C0948008
Ischemic stroke	C0948008	FLAD1	80308	Q8NFF5	flavin adenine dinucleotid			0.713	0.414	C0948008
Ischemic stroke	C0948008	KDM2B	84678	Q8NHM5	lysine demethylase 2B			0.713	0.414	C0948008
Ischemic stroke	C0948008	GJA4	2701	P35212	gap junc	cell junction pro		0.701	0.276	C0948008
Ischemic stroke	C0948008	RNLS	55328	Q5VYX0	renalase, FAD dependent am			0.701	0.379	C0948008
Ischemic stroke	C0948008	IMPA2	3613	O14732	inositol	hydrolase; phosph		0.696	0.276	C0948008
Ischemic stroke	C0948008	IL5RA	3568	Q01344	interleu	defense/immunity		0.69	0.552	C0948008
Ischemic stroke	C0948008	PSMA6	5687	P60900	proteaso	hydrolase; protea		0.69	0.379	C0948008
Ischemic stroke	C0948008	HOMER1	9456	Q86YM7	homer sca	signaling molecul		0.69	0.241	C0948008
Ischemic stroke	C0948008	TRPM4	54795	Q8TD43	transien	receptor; transpo		0.69	0.379	C0948008
Ischemic stroke	C0948008	DEFB104A	140596	Q8WTQ1	defensin beta 104A			0.69	0.483	C0948008
Ischemic stroke	C0948008	DEFB104B	503618	Q8WTQ1	defensin beta 104B			0.69	0.483	C0948008
Ischemic stroke	C0948008	LOC107984148	1.08E+08		beta-defensin 104A			0.69	0.483	C0948008
Ischemic stroke	C0948008	FGG	2266	P02679	fibrinog	signaling molecul		0.681	0.379	C0948008
Ischemic stroke	C0948008	KCNJ13	3769	O60928	potassium voltage-gated ch			0.681	0.172	C0948008
Ischemic stroke	C0948008	PRKCH	5583	P24723	protein	calcium-binding p		0.676	0.483	C0948008

Ischemic stroke	C0948008	SAA@	6287		serum amyloid A1 cluster		0.672	0.552	C0948008
Ischemic stroke	C0948008	GNLY	10578	P22749	granulysin		0.672	0.552	C0948008
Ischemic stroke	C0948008	SELENOS	55829	Q9BQE4	selenoprotein S		0.672	0.448	C0948008
Ischemic stroke	C0948008	MEPE	56955	Q9NQ76	matrix extracellular phosp		0.672	0.483	C0948008
Ischemic stroke	C0948008	SMURF2	64750	Q9HAU4	SMAD spe	ligase	0.672	0.448	C0948008
Ischemic stroke	C0948008	GAMT	2593	Q14353	guanidinoacetate N-methylt		0.667	0.241	C0948008
Ischemic stroke	C0948008	CIRBP	1153	Q14011	cold ind	nucleic acid bind	0.663	0.448	C0948008
Ischemic stroke	C0948008	TBXAS1	6916	P24557	thromboxane A synthase 1		0.663	0.552	C0948008
Ischemic stroke	C0948008	CYP2J2	1573	P51589	cytochrome P450 family 2 s		0.659	0.414	C0948008
Ischemic stroke	C0948008	SHC1	6464	P29353	SHC adap	signaling molecul	0.659	0.414	C0948008
Ischemic stroke	C0948008	MADD	8567	Q8WXG6	MAP kinase activating deat		0.659	0.414	C0948008
Ischemic stroke	C0948008	TRIB1	10221	Q96RU8	tribbles	kinase; transfera	0.659	0.379	C0948008
Ischemic stroke	C0948008	CAMKK2	10645	Q96RR4	calcium/calmodulin depende		0.659	0.517	C0948008
Ischemic stroke	C0948008	ANGPTL3	27329	Q9Y5C1	angiopoi	signaling molecul	0.659	0.276	C0948008
Ischemic stroke	C0948008	ZFHX3	463	Q15911	zinc finger homeobox 3		0.656	0.517	C0948008
Ischemic stroke	C0948008	F11	2160	P03951	coagulat	hydrolase; protea	0.656	0.414	C0948008
Ischemic stroke	C0948008	LTC4S	4056	Q16873	leukotri	transferase	0.656	0.483	C0948008
Ischemic stroke	C0948008	PLIN1	5346	O60240	perilipin 1		0.656	0.379	C0948008
Ischemic stroke	C0948008	IRF9	10379	Q00978	interfer	nucleic acid bind	0.656	0.552	C0948008
Ischemic stroke	C0948008	DUOX1	53905	Q9NRD9	dual oxi	oxidoreductase	0.656	0.517	C0948008
Ischemic stroke	C0948008	APLNR	187	P35414	apelin r	receptor	0.652	0.345	C0948008
Ischemic stroke	C0948008	GRM2	2912	Q14416	glutamat	receptor	0.645	0.414	C0948008
Ischemic stroke	C0948008	CARD8	22900	Q9Y2G2	caspase recruitment domain		0.645	0.552	C0948008
Ischemic stroke	C0948008	COL4A2	1284	P08572	collagen type IV alpha 2 c		0.642	0.448	C0948008
Ischemic stroke	C0948008	P2RY1	5028	P47900	purinergic receptor P2Y1		0.642	0.586	C0948008
Ischemic stroke	C0948008	SERPINI1	5274	Q99574	serpin f	enzyme modulator	0.642	0.31	C0948008
Ischemic stroke	C0948008	MASP2	10747	O00187	mannan b	hydrolase; protea	0.642	0.655	C0948008
Ischemic stroke	C0948008	P2RY12	64805	Q9H244	purinerg	receptor	0.642	0.379	C0948008
Ischemic stroke	C0948008	CYP2C8	1558	P10632	cytochrome P450 family 2 s		0.639	0.517	C0948008
Ischemic stroke	C0948008	GRIA2	2891	P42262	glutamate ionotropic recep		0.639	0.414	C0948008
Ischemic stroke	C0948008	HABP2	3026	Q14520	hyaluron	hydrolase; protea	0.639	0.448	C0948008
Ischemic stroke	C0948008	SERPINE2	5270	P07093	serpin f	enzyme modulator	0.639	0.517	C0948008
Ischemic stroke	C0948008	PTPRG	5793	P23470	protein	hydrolase; phosph	0.639	0.69	C0948008
Ischemic stroke	C0948008	GP6	51206	Q9HCN6	glycopro	defense/immunity	0.639	0.448	C0948008
Ischemic stroke	C0948008	VKORC1	79001	Q9BQB6	vitamin	oxidoreductase	0.639	0.655	C0948008
Ischemic stroke	C0948008	TNFSF12-TNFSF13	407977	O43508	TNFSF12-TNFSF13 readthroug		0.639	0.517	C0948008
Ischemic stroke	C0948008	FADS1	3992	O60427	fatty acid desaturase 1		0.636	0.621	C0948008
Ischemic stroke	C0948008	LTA4H	4048	P09960	leukotriene A4 hydrolase		0.636	0.483	C0948008
Ischemic stroke	C0948008	NEIL1	79661	Q96FI4	nei like DNA glycosylase 1		0.636	0.586	C0948008
Ischemic stroke	C0948008	ZC3H12A	80149	Q5D1E8	zinc fin	nucleic acid bind	0.636	0.552	C0948008
Ischemic stroke	C0948008	ALOX5AP	241	P20292	arachido	transferase	0.633	0.552	C0948008
Ischemic stroke	C0948008	EPHX2	2053	P34913	epoxide	hydrolase; protea	0.633	0.448	C0948008
Ischemic stroke	C0948008	CARTPT	9607	Q16568	CART prepropeptide		0.633	0.448	C0948008
Ischemic stroke	C0948008	GRIN1	2902	Q05586	glutamate ionotropic recep		0.63	0.448	C0948008
Ischemic stroke	C0948008	CAPN10	11132	Q9HC96	calpain	calcium-binding p	0.63	0.483	C0948008
Ischemic stroke	C0948008	PDE11A	50940	Q9HCR9	phosphodiesterase 11A		0.627	0.517	C0948008
Ischemic stroke	C0948008	BARD1	580	Q99728	BRCA1 associated RING doma		0.624	0.379	C0948008
Ischemic stroke	C0948008	PTGIS	5740	Q16647	prostaglandin I2 synthase		0.624	0.552	C0948008
Ischemic stroke	C0948008	ACD	65057	Q96AP0	ACD, shelterin complex sub		0.624	0.69	C0948008
Ischemic stroke	C0948008	GRIK2	2898	Q13002	glutamate ionotropic recep		0.621	0.586	C0948008
Ischemic stroke	C0948008	SELPLG	6404	Q14242	selectin P ligand		0.621	0.517	C0948008
Ischemic stroke	C0948008	PLK2	10769	Q9NYY3	polo like kinase 2		0.621	0.586	C0948008
Ischemic stroke	C0948008	REM1	28954	O75628	RRAD and GEM like GTPase 1		0.621	0.655	C0948008
Ischemic stroke	C0948008	EDIL3	10085	O43854	EGF like	cell adhesion mol	0.619	0.517	C0948008
Ischemic stroke	C0948008	SORBS1	10580	Q9BX66	sorbin and SH3 domain cont		0.619	0.724	C0948008
Ischemic stroke	C0948008	BHMT	635	Q93088	betaine--homocysteine S-me		0.616	0.483	C0948008
Ischemic stroke	C0948008	MUT	4594	P22033	methylma	isomerase	0.616	0.655	C0948008
Ischemic stroke	C0948008	CHDH	55349	Q8NE62	choline	oxidoreductase	0.616	0.517	C0948008
Ischemic stroke	C0948008	RAPSN	5913	Q13702	receptor associated protei		0.614	0.586	C0948008
Ischemic stroke	C0948008	APOH	350	P02749	apolipoprotein H		0.611	0.724	C0948008
Ischemic stroke	C0948008	TLR8	51311	Q9NR97	toll like receptor 8		0.611	0.655	C0948008
Ischemic stroke	C0948008	MUSK	4593	O15146	muscle associated receptor		0.609	0.621	C0948008
Ischemic stroke	C0948008	APOA5	116519	Q6Q788	apolipoprotein A5		0.609	0.483	C0948008

Ischemic stroke	C0948008	ADD1	118	P35611	adducin	cytoskeletal prot	0.607	0.517	C0948008
Ischemic stroke	C0948008	PTGER2	5732	P43116	prostagla	receptor	0.607	0.621	C0948008
Ischemic stroke	C0948008	SHMT1	6470	P34896	serine hy	transferase	0.607	0.517	C0948008
Ischemic stroke	C0948008	IL17B	27190	Q9UHF5	interleukin 17B		0.607	0.655	C0948008
Ischemic stroke	C0948008	FGB	2244	P02675	fibrinoge	signaling molecul	0.604	0.552	C0948008
Ischemic stroke	C0948008	BTF3P11	690		basic transcription factor		0.602	0.552	C0948008
Ischemic stroke	C0948008	F13A1	2162	P00488	coagulati	transferase	0.602	0.586	C0948008
Ischemic stroke	C0948008	SAA1	6288	P0DJI8	serum amy	defense/immunity	0.602	0.655	C0948008
Ischemic stroke	C0948008	KLF2	10365	Q9Y5W3	Kruppel l	nucleic acid bind	0.602	0.586	C0948008
Ischemic stroke	C0948008	CRTC1	23373	Q6UUV9	CREB regu	transcription fac	0.602	0.621	C0948008
Ischemic stroke	C0948008	MIR424	494336		microRNA 424		0.6	0.552	C0948008
Ischemic stroke	C0948008	SRF	6722	P11831	serum res	transcription fac	0.598	0.69	C0948008
Ischemic stroke	C0948008	TNFSF12	8742	O43508	TNF superfamily member 12		0.598	0.655	C0948008
Ischemic stroke	C0948008	PROCR	10544	Q9UNN8	protein C	enzyme modulator;	0.598	0.552	C0948008
Ischemic stroke	C0948008	DUOX2	50506	Q9NRD8	dual oxid	oxidoreductase	0.598	0.69	C0948008
Ischemic stroke	C0948008	ALOX12	239	P18054	arachidon	oxidoreductase	0.596	0.621	C0948008
Ischemic stroke	C0948008	PON2	5445	Q15165	paraoxonase 2		0.596	0.621	C0948008
Ischemic stroke	C0948008	CPB2	1361	Q96IY4	carboxype	hydrolase; protea	0.594	0.586	C0948008
Ischemic stroke	C0948008	FGA	2243	P02671	fibrinogen alpha chain		0.594	0.586	C0948008
Ischemic stroke	C0948008	SLC2A10	81031	O95528	solute ca	transporter	0.594	0.621	C0948008
Ischemic stroke	C0948008	TCN2	6948	P20062	transcobalamin 2		0.592	0.517	C0948008
Ischemic stroke	C0948008	PADI4	23569	Q9UM07	peptidyl arginine deiminas		0.592	0.586	C0948008
Ischemic stroke	C0948008	CEACAM1	634	P13688	carcinoembryonic antigen r		0.59	0.552	C0948008
Ischemic stroke	C0948008	FABP4	2167	P15090	fatty acid binding protein		0.59	0.552	C0948008
Ischemic stroke	C0948008	OLR1	4973	P78380	oxidized low density lipop		0.59	0.552	C0948008
Ischemic stroke	C0948008	APOL1	8542	O14791	apolipopr	transfer/carrier	0.59	0.621	C0948008
Ischemic stroke	C0948008	GLA	2717	P06280	galactosidase alpha		0.588	0.621	C0948008
Ischemic stroke	C0948008	LRP6	4040	O75581	LDL receptor related prote		0.588	0.724	C0948008
Ischemic stroke	C0948008	F8	2157	P00451	coagulati	cell adhesion mol	0.586	0.655	C0948008
Ischemic stroke	C0948008	CCL11	6356	P51671	C-C motif	signaling molecul	0.586	0.655	C0948008
Ischemic stroke	C0948008	MIR107	406901		microRNA 107		0.586	0.621	C0948008
Ischemic stroke	C0948008	FOLH1	2346	Q04609	folate hy	hydrolase; protea	0.584	0.724	C0948008
Ischemic stroke	C0948008	REST	5978	Q13127	RE1 silen	transcription fac	0.584	0.517	C0948008
Ischemic stroke	C0948008	PLA2G7	7941	Q13093	phosphol	hydrolase	0.584	0.552	C0948008
Ischemic stroke	C0948008	TRPM7	54822	Q96QT4	transient	receptor; transpo	0.584	0.552	C0948008
Ischemic stroke	C0948008	BCL2A1	597	Q16548	BCL2 rela	signaling molecul	0.582	0.621	C0948008
Ischemic stroke	C0948008	DVL1	1855	O14640	dishevel	enzyme modulator;	0.582	0.655	C0948008
Ischemic stroke	C0948008	TNFSF4	7292	P23510	TNF super	signaling molecul	0.582	0.69	C0948008
Ischemic stroke	C0948008	CHIT1	1118	Q13231	chitinase 1		0.58	0.621	C0948008
Ischemic stroke	C0948008	MMP10	4319	P09238	matrix me	hydrolase; protea	0.58	0.759	C0948008
Ischemic stroke	C0948008	TNFSF14	8740	O43557	TNF super	signaling molecul	0.58	0.586	C0948008
Ischemic stroke	C0948008	MIR497	574456		microRNA 497		0.58	0.586	C0948008
Ischemic stroke	C0948008	AHSG	197	P02765	alpha 2-H	enzyme modulator	0.577	0.69	C0948008
Ischemic stroke	C0948008	GPR42	2866	O15529	G protein-coupled receptor		0.577	0.69	C0948008
Ischemic stroke	C0948008	MLXIPL	51085	Q9NP71	MLX interacting protein li		0.577	0.655	C0948008
Ischemic stroke	C0948008	PINK1	65018	Q9BXM7	PTEN indu	kinase; receptor;	0.577	0.586	C0948008
Ischemic stroke	C0948008	GPX3	2878	P22352	glutathio	oxidoreductase	0.575	0.586	C0948008
Ischemic stroke	C0948008	IL20	50604	Q9NYY1	interleukin 20		0.575	0.69	C0948008
Ischemic stroke	C0948008	SERPINA3	12	P01011	serpin fa	enzyme modulator	0.572	0.69	C0948008
Ischemic stroke	C0948008	CYP11B2	1585	P19099	cytochrome P450 family 11		0.572	0.483	C0948008
Ischemic stroke	C0948008	PDE4D	5144	Q08499	phosphodiesterase 4D		0.572	0.69	C0948008
Ischemic stroke	C0948008	SLC1A2	6506	P43004	solute ca	transporter	0.572	0.517	C0948008
Ischemic stroke	C0948008	TREM2	54209	Q9NZC2	triggering receptor expres		0.572	0.552	C0948008
Ischemic stroke	C0948008	PCSK9	255738	Q8NBP7	proprote	hydrolase; protea	0.572	0.552	C0948008
Ischemic stroke	C0948008	ACVRL1	94	P37023	activin A	kinase; receptor;	0.57	0.621	C0948008
Ischemic stroke	C0948008	PGR-AS1	1.01E+08		PGR antisense RNA 1		0.57	0.586	C0948008
Ischemic stroke	C0948008	BRS3	680	P32247	bombesin receptor subtype		0.569	0.69	C0948008
Ischemic stroke	C0948008	MIR132	406921		microRNA 132		0.569	0.759	C0948008
Ischemic stroke	C0948008	NPPB	4879	P16860	natriuretic peptide B		0.567	0.552	C0948008
Ischemic stroke	C0948008	ITGA2	3673	P17301	integrin subunit alpha 2		0.565	0.655	C0948008
Ischemic stroke	C0948008	PC	5091	P11498	pyruvate	ligase	0.565	0.724	C0948008
Ischemic stroke	C0948008	ORAI1	84876	Q96D31	ORAI calcium release-activ		0.565	0.655	C0948008
Ischemic stroke	C0948008	A2M	2	P01023	alpha-2-m	defense/immunity	0.564	0.724	C0948008

Ischemic stroke	C0948008	CIITA	4261	P33076	class II major histocompat		0.564	0.724	C0948008
Ischemic stroke	C0948008	CCL22	6367	O00626	C-C moti	signaling molecul	0.564	0.69	C0948008
Ischemic stroke	C0948008	NAMPT	10135	P43490	nicotinar	signaling molecul	0.564	0.69	C0948008
Ischemic stroke	C0948008	PTGES	9536	O14684	prostaglandin E synthase		0.562	0.69	C0948008
Ischemic stroke	C0948008	MIR499A	574501		microRNA 499a		0.562	0.69	C0948008
Ischemic stroke	C0948008	SSTR4	6754	P31391	somatosta	receptor	0.558	0.69	C0948008
Ischemic stroke	C0948008	MIR29B1	407024		microRNA 29b-1		0.558	0.69	C0948008
Ischemic stroke	C0948008	DEFB4B	1E+08	O15263	defensin	defense/immunity	0.558	0.724	C0948008
Ischemic stroke	C0948008	CAD	790	P27708	carbamoy	hydrolase; ligase	0.557	0.655	C0948008
Ischemic stroke	C0948008	C20orf181	1E+08		chromosome 20 open reading		0.557	0.69	C0948008
Ischemic stroke	C0948008	CETP	1071	P11597	cholesteryl ester transfer		0.555	0.655	C0948008
Ischemic stroke	C0948008	DEFB1	1672	P60022	defensin beta 1		0.555	0.655	C0948008
Ischemic stroke	C0948008	SERPINC1	462	P01008	serpin fa	enzyme modulator	0.554	0.621	C0948008
Ischemic stroke	C0948008	CRYZ	1429	Q08257	crystallin zeta		0.554	0.621	C0948008
Ischemic stroke	C0948008	MOK	5891	Q9UQ07	MOK prot	kinase; transfera	0.554	0.655	C0948008
Ischemic stroke	C0948008	STIM1	6786	Q13586	stromal interaction molecu		0.554	0.655	C0948008
Ischemic stroke	C0948008	MIR29B2	407025		microRNA 29b-2		0.554	0.724	C0948008
Ischemic stroke	C0948008	SLC19A1	6573	P41440	solute ca	transporter	0.552	0.621	C0948008
Ischemic stroke	C0948008	DEFB4A	1673	O15263	defensin	defense/immunity	0.551	0.724	C0948008
Ischemic stroke	C0948008	SLC33A1	9197	O00400	solute ca	transporter	0.551	0.655	C0948008
Ischemic stroke	C0948008	CYBA	1535	P13498	cytochrome b-245 alpha cha		0.55	0.655	C0948008
Ischemic stroke	C0948008	FLG	2312	P20930	filaggrin		0.55	0.655	C0948008
Ischemic stroke	C0948008	CDKN2B-AS1	1E+08		CDKN2B antisense RNA 1		0.55	0.586	C0948008
Ischemic stroke	C0948008	ADRA1A	148	P35348	adrenocer	receptor	0.548	0.69	C0948008
Ischemic stroke	C0948008	ITGA2B	3674	P08514	integrin subunit alpha 2b		0.548	0.517	C0948008
Ischemic stroke	C0948008	F7	2155	P08709	coagulat	hydrolase; protea	0.547	0.69	C0948008
Ischemic stroke	C0948008	IRF4	3662	Q15306	interfer	nucleic acid bind	0.547	0.517	C0948008
Ischemic stroke	C0948008	TNFRSF12A	51330	Q9NP84	TNF receptor superfamily m		0.547	0.586	C0948008
Ischemic stroke	C0948008	NPPA	4878	P01160	natriuretic peptide A		0.546	0.69	C0948008
Ischemic stroke	C0948008	ADRA2B	151	P18089	adrenocer	receptor	0.545	0.69	C0948008
Ischemic stroke	C0948008	WRN	7486	Q14191	Werner s	nucleic acid bind	0.545	0.586	C0948008
Ischemic stroke	C0948008	TFPI2	7980	P48307	tissue fa	enzyme modulator	0.545	0.621	C0948008
Ischemic stroke	C0948008	NR4A3	8013	Q92570	nuclear r	receptor; transcr	0.545	0.759	C0948008
Ischemic stroke	C0948008	VPS51	738	Q9UID3	VPS51, GARP complex subuni		0.543	0.655	C0948008
Ischemic stroke	C0948008	IL16	3603	Q14005	interleu	signaling molecul	0.543	0.793	C0948008
Ischemic stroke	C0948008	PRH1	5554	P02810	proline rich protein HaeII		0.543	0.69	C0948008
Ischemic stroke	C0948008	CDK5	1020	Q00535	cyclin de	kinase; transfera	0.542	0.69	C0948008
Ischemic stroke	C0948008	CYP2C9	1559	P11712	cytochrome P450 family 2 s		0.542	0.759	C0948008
Ischemic stroke	C0948008	PDGFA	5154	P04085	platelet	signaling molecul	0.542	0.621	C0948008
Ischemic stroke	C0948008	TLR7	51284	Q9NYK1	toll like receptor 7		0.542	0.759	C0948008
Ischemic stroke	C0948008	ACE2	59272	Q9BYF1	angiotens	hydrolase; protea	0.542	0.655	C0948008
Ischemic stroke	C0948008	ACTA2	59	P62736	actin, a	cytoskeletal prot	0.54	0.69	C0948008
Ischemic stroke	C0948008	MIP	4284	P30301	major in	transporter	0.54	0.759	C0948008
Ischemic stroke	C0948008	SULT1E1	6783	P49888	sulfotransferase family 1E		0.538	0.759	C0948008
Ischemic stroke	C0948008	LPAR2	9170	Q9HBW0	lysophos	receptor	0.537	0.69	C0948008
Ischemic stroke	C0948008	PPIG	9360	Q13427	peptidylprolyl isomerase G		0.537	0.793	C0948008
Ischemic stroke	C0948008	PIN1	5300	Q13526	peptidylprolyl cis/trans i		0.536	0.655	C0948008
Ischemic stroke	C0948008	ADAMTS13	11093	Q76LX8	ADAM meta	enzyme modulator;	0.536	0.69	C0948008
Ischemic stroke	C0948008	MIR143	406935		microRNA 143		0.536	0.621	C0948008
Ischemic stroke	C0948008	GCH1	2643	P30793	GTP cycl	hydrolase	0.534	0.724	C0948008
Ischemic stroke	C0948008	GNB3	2784	P16520	G protei	enzyme modulator;	0.533	0.724	C0948008
Ischemic stroke	C0948008	LRP1	4035	Q07954	LDL receptor related prote		0.533	0.724	C0948008
Ischemic stroke	C0948008	FGF13	2258	Q92913	fibrobla	signaling molecul	0.532	0.621	C0948008
Ischemic stroke	C0948008	IL9	3578	P15248	interleukin 9		0.53	0.69	C0948008
Ischemic stroke	C0948008	SGK1	6446	O00141	serum/gl	calcium-binding p	0.53	0.621	C0948008
Ischemic stroke	C0948008	UCP2	7351	P55851	uncoupling protein 2		0.53	0.69	C0948008
Ischemic stroke	C0948008	LIF	3976	P15018	LIF, int	signaling molecul	0.528	0.655	C0948008
Ischemic stroke	C0948008	TRAF6	7189	Q9Y4K3	TNF rece	signaling molecul	0.528	0.724	C0948008
Ischemic stroke	C0948008	MSC	9242	O60682	musculin		0.526	0.655	C0948008
Ischemic stroke	C0948008	AQP4	361	P55087	aquapori	transporter	0.525	0.793	C0948008
Ischemic stroke	C0948008	KCNQ1	3784	P51787	potassium voltage-gated ch		0.525	0.586	C0948008
Ischemic stroke	C0948008	NTF3	4908	P20783	neurotro	signaling molecul	0.525	0.621	C0948008
Ischemic stroke	C0948008	CXCR6	10663	O00574	C-X-C motif chemokine rece		0.525	0.793	C0948008

Ischemic stroke	C0948008	MMRN1	22915	Q13201	multimer	extracellular mat	0.523	0.655	C0948008
Ischemic stroke	C0948008	NR3C2	4306	P08235	nuclear	nucleic acid bind	0.52	0.69	C0948008
Ischemic stroke	C0948008	SELP	6403	P16109	selectin P		0.519	0.724	C0948008
Ischemic stroke	C0948008	CDKN3	1033	Q16667	cyclin dependent kinase in		0.518	0.724	C0948008
Ischemic stroke	C0948008	HSPA1A	3303	P0DMV8;P(	heat shock protein family		0.516	0.793	C0948008
Ischemic stroke	C0948008	MMP12	4321	P39900	matrix m	hydrolase; protea	0.516	0.862	C0948008
Ischemic stroke	C0948008	HSPA1B	3304	P0DMV8;P(	heat shock protein family		0.514	0.793	C0948008
Ischemic stroke	C0948008	COL4A1	1282	P02462	collagen type IV alpha 1 c		0.512	0.69	C0948008
Ischemic stroke	C0948008	MIR223	407008		microRNA 223		0.512	0.759	C0948008
Ischemic stroke	C0948008	EEF1A2	1917	Q05639	eukaryot	enzyme modulator;	0.51	0.69	C0948008
Ischemic stroke	C0948008	MTRR	4552	Q9UBK8	5-methyl	tetrahydrofolate-h	0.51	0.655	C0948008
Ischemic stroke	C0948008	PITX2	5308	Q99697	paired like homeodomain 2		0.51	0.724	C0948008
Ischemic stroke	C0948008	PLA2G2A	5320	P14555	phosphol	hydrolase	0.509	0.69	C0948008
Ischemic stroke	C0948008	ABCC8	6833	Q09428	ATP bind	transporter	0.508	0.69	C0948008
Ischemic stroke	C0948008	ITGB3	3690	P05106	integrin	cell adhesion mol	0.507	0.69	C0948008
Ischemic stroke	C0948008	NRG1	3084	Q02297	neuregul	signaling molecul	0.506	0.759	C0948008
Ischemic stroke	C0948008	THBD	7056	P07204	thrombomodulin		0.505	0.759	C0948008
Ischemic stroke	C0948008	LPL	4023	P06858	lipoprot	hydrolase; storag	0.504	0.724	C0948008
Ischemic stroke	C0948008	IL21	59067	Q9HBE4	interleukin 21		0.504	0.759	C0948008
Ischemic stroke	C0948008	P2RX7	5027	Q99572	purinerg	receptor; transpo	0.503	0.759	C0948008
Ischemic stroke	C0948008	CHI3L1	1116	P36222	chitinase 3 like 1		0.502	0.793	C0948008
Ischemic stroke	C0948008	PLA2G1B	5319	P04054	phosphol	hydrolase	0.502	0.793	C0948008
Ischemic stroke	C0948008	CYP2C19	1557	P33261	cytochrome P450 family 2 s		0.501	0.793	C0948008
Ischemic stroke	C0948008	HDAC9	9734	Q9UKV0	histone deacetylase 9		0.501	0.759	C0948008
Ischemic stroke	C0948008	GDF15	9518	Q99988	growth d	signaling molecul	0.496	0.621	C0948008
Ischemic stroke	C0948008	ANGPT1	284	Q15389	angiopoi	signaling molecul	0.495	0.69	C0948008
Ischemic stroke	C0948008	PTGS1	5742	P23219	prostagl	oxidoreductase	0.495	0.828	C0948008
Ischemic stroke	C0948008	PLA2G6	8398	O60733	phosphol	enzyme modulator	0.494	0.724	C0948008
Ischemic stroke	C0948008	ACKR3	57007	P25106	atypical chemokine recepto		0.494	0.759	C0948008
Ischemic stroke	C0948008	MTR	4548	Q99707	5-methyl	tetrahydrofolate-h	0.493	0.724	C0948008
Ischemic stroke	C0948008	EDNRB	1910	P24530	endothelin receptor type B		0.492	0.793	C0948008
Ischemic stroke	C0948008	PECAM1	5175	P16284	platelet	cell adhesion mol	0.492	0.69	C0948008
Ischemic stroke	C0948008	ABO	28	P16442	ABO, alp	transferase	0.491	0.828	C0948008
Ischemic stroke	C0948008	UGT1A1	54658	P22309	UDP glucuronosyltransferas		0.491	0.724	C0948008
Ischemic stroke	C0948008	LPA	4018	P08519	lipoprot	hydrolase; protea	0.489	0.655	C0948008
Ischemic stroke	C0948008	AVP	551	P01185	arginine	signaling molecul	0.486	0.759	C0948008
Ischemic stroke	C0948008	ADIPOQ	9370	Q15848	adiponectin, C1Q and colla		0.485	0.724	C0948008
Ischemic stroke	C0948008	ABCA1	19	O95477	ATP bind	transporter	0.484	0.793	C0948008
Ischemic stroke	C0948008	ALOX5	240	P09917	arachido	oxidoreductase	0.482	0.724	C0948008
Ischemic stroke	C0948008	KCNA3	3738	P22001	potassium voltage-gated ch		0.481	0.724	C0948008
Ischemic stroke	C0948008	PRH2	5555	P02810	proline rich protein HaeII		0.48	0.759	C0948008
Ischemic stroke	C0948008	ADRB2	154	P07550	adrenoce	receptor	0.479	0.793	C0948008
Ischemic stroke	C0948008	ANGPT2	285	O15123	angiopoi	signaling molecul	0.479	0.724	C0948008
Ischemic stroke	C0948008	BIRC5	332	O15392	baculovi	enzyme modulator	0.479	0.655	C0948008
Ischemic stroke	C0948008	SELE	6401	P16581	selectin E		0.479	0.793	C0948008
Ischemic stroke	C0948008	F2R	2149	P25116	coagulation factor II thro		0.478	0.759	C0948008
Ischemic stroke	C0948008	CFH	3075	P08603	complement factor H		0.478	0.793	C0948008
Ischemic stroke	C0948008	APOB	338	P04114	apolipoprotein B		0.477	0.724	C0948008
Ischemic stroke	C0948008	ENG	2022	P17813	endoglin		0.477	0.655	C0948008
Ischemic stroke	C0948008	G6PD	2539	P11413	glucose-(	oxidoreductase	0.476	0.724	C0948008
Ischemic stroke	C0948008	OGG1	4968	O15527	8-oxoguanine DNA glycosyla		0.476	0.759	C0948008
Ischemic stroke	C0948008	LDLR	3949	P01130	low density lipoprotein re		0.475	0.828	C0948008
Ischemic stroke	C0948008	TLR3	7098	O15455	toll like	extracellular mat	0.475	0.862	C0948008
Ischemic stroke	C0948008	PRNP	5621	F7VJQ1;P(	prion protein		0.474	0.897	C0948008
Ischemic stroke	C0948008	AGER	177	Q15109	advanced	cell adhesion mol	0.472	0.724	C0948008
Ischemic stroke	C0948008	ADM	133	P35318	adrenome	signaling molecul	0.471	0.724	C0948008
Ischemic stroke	C0948008	MMP7	4316	P09237	matrix m	hydrolase; protea	0.47	0.724	C0948008
Ischemic stroke	C0948008	EDNRA	1909	P25101	endothelin receptor type A		0.469	0.828	C0948008
Ischemic stroke	C0948008	FBN1	2200	P35555	fibrilli	calcium-binding p	0.469	0.69	C0948008
Ischemic stroke	C0948008	NPY	4852	P01303	neuropep	signaling molecul	0.469	0.69	C0948008
Ischemic stroke	C0948008	NOTCH3	4854	Q9UM47	notch 3		0.469	0.69	C0948008
Ischemic stroke	C0948008	F5	2153	P12259	coagulat	cell adhesion mol	0.468	0.69	C0948008
Ischemic stroke	C0948008	SEMA6A	57556	Q9H2E6	semaphor	signaling molecul	0.468	0.793	C0948008

Ischemic stroke	C0948008	NQO1	1728	P15559	NAD(P)H quinone dehydrogen		0.467	0.724	C0948008
Ischemic stroke	C0948008	TNFRSF11B	4982	O00300	TNF receptor superfamily m		0.467	0.69	C0948008
Ischemic stroke	C0948008	TIMP2	7077	P16035	TIMP meta	enzyme modulator	0.467	0.759	C0948008
Ischemic stroke	C0948008	F2	2147	P00734	coagulat	hydrolase; protea	0.466	0.793	C0948008
Ischemic stroke	C0948008	IFNB1	3456	P01574	interferon beta 1		0.466	0.793	C0948008
Ischemic stroke	C0948008	VCAM1	7412	P19320	vascular cell adhesion mol		0.466	0.793	C0948008
Ischemic stroke	C0948008	PPARA	5465	Q07869	peroxiso	nucleic acid bind	0.465	0.793	C0948008
Ischemic stroke	C0948008	APOA1	335	P02647	apolipoprotein A1		0.463	0.759	C0948008
Ischemic stroke	C0948008	IL5	3567	P05113	interleukin 5		0.463	0.828	C0948008
Ischemic stroke	C0948008	LTA	4049	P01374	lymphoto	signaling molecul	0.463	0.862	C0948008
Ischemic stroke	C0948008	VWF	7450	P04275	von Will	enzyme modulator	0.462	0.724	C0948008
Ischemic stroke	C0948008	COX8A	1351	P10176	cytochro	oxidoreductase	0.461	0.793	C0948008
Ischemic stroke	C0948008	EGR1	1958	P18146	early gr	nucleic acid bind	0.459	0.793	C0948008
Ischemic stroke	C0948008	PLAT	5327	P00750	plasmino	hydrolase; protea	0.459	0.828	C0948008
Ischemic stroke	C0948008	F3	2152	P13726	coagulat	defense/immunity	0.458	0.724	C0948008
Ischemic stroke	C0948008	AGTR1	185	P30556	angioten	receptor	0.454	0.759	C0948008
Ischemic stroke	C0948008	MIR146A	406938		microRNA 146a		0.452	0.759	C0948008
Ischemic stroke	C0948008	XRCC1	7515	P18887	X-ray repair cross complem		0.449	0.828	C0948008
Ischemic stroke	C0948008	ABCG2	9429	Q9UNQ0	ATP bind	transporter	0.449	0.793	C0948008
Ischemic stroke	C0948008	CD40LG	959	P29965	CD40 lig	signaling molecul	0.447	0.828	C0948008
Ischemic stroke	C0948008	ESR2	2100	Q92731	estrogen	nucleic acid bind	0.445	0.828	C0948008
Ischemic stroke	C0948008	PON1	5444	P27169	paraoxonase 1		0.443	0.828	C0948008
Ischemic stroke	C0948008	HSPA4	3308	P34932	heat shock protein family		0.441	0.793	C0948008
Ischemic stroke	C0948008	HFE	3077	Q30201	homeostatic iron regulator		0.44	0.69	C0948008
Ischemic stroke	C0948008	MMP3	4314	P08254	matrix m	hydrolase; protea	0.439	0.793	C0948008
Ischemic stroke	C0948008	CDKN2B	1030	P42772	cyclin dependent kinase in		0.438	0.759	C0948008
Ischemic stroke	C0948008	CYP2B6	1555	P20813	cytochrome P450 family 2 s		0.437	0.828	C0948008
Ischemic stroke	C0948008	MPO	4353	P05164	myeloper	oxidoreductase	0.435	0.931	C0948008
Ischemic stroke	C0948008	PLAU	5328	P00749	plasmino	hydrolase; protea	0.434	0.69	C0948008
Ischemic stroke	C0948008	TBC1D9	23158	Q6ZT07	TBC1 dom	enzyme modulator;	0.434	0.828	C0948008
Ischemic stroke	C0948008	CD40	958	P25942	CD40 molecule		0.433	0.862	C0948008
Ischemic stroke	C0948008	HMGB1	3146	P09429	high mob	nucleic acid bind	0.433	0.828	C0948008
Ischemic stroke	C0948008	IGF1R	3480	P08069	insulin like growth factor		0.433	0.828	C0948008
Ischemic stroke	C0948008	BRCA2	675	P51587	BRCA2, D	nucleic acid bind	0.426	0.793	C0948008
Ischemic stroke	C0948008	COMT	1312	P21964	catechol-	transferase	0.426	0.897	C0948008
Ischemic stroke	C0948008	EPHB2	2048	P29323	EPH receptor B2		0.425	0.759	C0948008
Ischemic stroke	C0948008	EPO	2056	P01588	erythropoietin		0.425	0.759	C0948008
Ischemic stroke	C0948008	KDR	3791	P35968	kinase insert domain recep		0.423	0.724	C0948008
Ischemic stroke	C0948008	REN	5972	P00797	renin	hydrolase; protea	0.423	0.759	C0948008
Ischemic stroke	C0948008	CSF3	1440	P09919	colony stimulating factor		0.421	0.828	C0948008
Ischemic stroke	C0948008	EZH2	2146	Q15910	enhancer of zeste 2 polyco		0.421	0.759	C0948008
Ischemic stroke	C0948008	PLG	5340	P00747	plasmino	hydrolase; protea	0.419	0.862	C0948008
Ischemic stroke	C0948008	MMP1	4312	P03956	matrix m	hydrolase; protea	0.415	0.793	C0948008
Ischemic stroke	C0948008	NOTCH1	4851	P46531	notch 1		0.415	0.759	C0948008
Ischemic stroke	C0948008	MIR21	406991		microRNA 21		0.407	0.759	C0948008
Ischemic stroke	C0948008	AGT	183	P01019	angioten	enzyme modulator	0.406	0.862	C0948008
Ischemic stroke	C0948008	FGF2	2247	P09038	fibrobla	signaling molecul	0.404	0.897	C0948008
Ischemic stroke	C0948008	IL18	3606	Q14116	interleukin 18		0.404	0.862	C0948008
Ischemic stroke	C0948008	HMOX1	3162	P09601	heme oxy	oxidoreductase	0.403	0.862	C0948008
Ischemic stroke	C0948008	ATM	472	Q13315	ATM seri	kinase; nucleic a	0.401	0.862	C0948008
Ischemic stroke	C0948008	NOS3	4846	P29474	nitric oxide synthase 3		0.401	0.862	C0948008
Ischemic stroke	C0948008	CRP	1401	P02741	C-reactive protein		0.399	0.862	C0948008
Ischemic stroke	C0948008	IL1RN	3557	P18510	interleukin 1 receptor ant		0.398	0.897	C0948008
Ischemic stroke	C0948008	COX2	4513	P00403	cytochro	oxidoreductase	0.397	0.862	C0948008
Ischemic stroke	C0948008	IL17A	3605	Q16552	interleukin 17A		0.391	0.862	C0948008
Ischemic stroke	C0948008	SPP1	6696	P10451	secreted phosphoprotein 1		0.391	0.862	C0948008
Ischemic stroke	C0948008	PPARG	5468	P37231	peroxiso	nucleic acid bind	0.388	0.828	C0948008
Ischemic stroke	C0948008	MAPK1	5594	P28482	mitogen-	kinase; transfera	0.387	0.862	C0948008
Ischemic stroke	C0948008	ICAM1	3383	P05362	intercellular adhesion mol		0.386	0.897	C0948008
Ischemic stroke	C0948008	SERPINE1	5054	P05121	serpin f	enzyme modulator	0.384	0.793	C0948008
Ischemic stroke	C0948008	BDNF	627	P23560	brain de	signaling molecul	0.382	0.828	C0948008
Ischemic stroke	C0948008	AR	367	P10275	androgen	nucleic acid bind	0.38	0.793	C0948008
Ischemic stroke	C0948008	MMP2	4313	P08253	matrix m	hydrolase; protea	0.373	0.862	C0948008

Ischemic stroke	C0948008	ABCB1	5243	P08183	ATP bind	hydrolase; protea	0.371	0.828	C0948008
Ischemic stroke	C0948008	ACE	1636	P12821	angiotens	hydrolase; protea	0.367	0.897	C0948008
Ischemic stroke	C0948008	IL1A	3552	P01583	interleukin 1 alpha		0.366	0.931	C0948008
Ischemic stroke	C0948008	IL4	3565	P05112	interleukin 4		0.364	0.931	C0948008
Ischemic stroke	C0948008	TLR4	7099	O00206	toll like receptor 4		0.359	0.931	C0948008
Ischemic stroke	C0948008	ESR1	2099	P03372	estrogen	nucleic acid bind	0.355	0.897	C0948008
Ischemic stroke	C0948008	IGF1	3479	P05019	insulin like growth factor		0.355	0.828	C0948008
Ischemic stroke	C0948008	MTHFR	4524	P42898	methylenetetrahydrofolate		0.354	0.828	C0948008
Ischemic stroke	C0948008	APOE	348	P02649	apolipoprotein E		0.352	0.931	C0948008
Ischemic stroke	C0948008	CXCL8	3576	P10145	C-X-C mo	signaling molecul	0.342	0.862	C0948008
Ischemic stroke	C0948008	MMP9	4318	P14780	matrix m	hydrolase; protea	0.339	0.862	C0948008
Ischemic stroke	C0948008	PTGS2	5743	P35354	prostagla	oxidoreductase	0.338	0.897	C0948008
Ischemic stroke	C0948008	TGFB1	7040	P01137	transfor	signaling molecul	0.336	0.931	C0948008
Ischemic stroke	C0948008	CDKN2A	1029	P42771;Q8	cyclin dependent kinase in		0.321	0.828	C0948008
Ischemic stroke	C0948008	IL10	3586	P22301	interleukin 10		0.32	0.931	C0948008
Ischemic stroke	C0948008	IL1B	3553	P01584	interleukin 1 beta		0.312	0.931	C0948008
Ischemic stroke	C0948008	VEGFA	7422	P15692	vascular	signaling molecul	0.298	0.897	C0948008
Ischemic stroke	C0948008	IL6	3569	P05231	interleukin 6		0.287	0.966	C0948008
Ischemic stroke	C0948008	TNF	7124	P01375	tumor ne	signaling molecul	0.263	0.966	C0948008
Ischemic stroke	C0948008	TP53	7157	P04637	tumor pr	transcription fac	0.251	0.897	C0948008

Additional file4 Final 94 Ischemic stroke genes

Index_disease	Index_dis	Gene	Gene_id	UniProt	Gene_Full	Protein_C	pLI	DSI_g	DPI_g	diseaseid	94 IS genes	retrieve genes	312 IS-related genes
Ischemic stroke	C0948008	MIAT	440823		myocardial infarction assoc			0.799	0.207	C0948008	MIAT	MIAT	ALOX5AP
Ischemic stroke	C0948008	F12	2161	P00748	coagulati	hydrolase; proteas		0.727	0.379	C0948008	F12	F12	F2
Ischemic stroke	C0948008	PRKCH	5583	P24723	protein k	calcium-binding pr		0.676	0.483	C0948008	PRKCH	PRKCH	F5
Ischemic stroke	C0948008	ZFHX3	463	Q15911	zinc finger homeobox 3			0.656	0.517	C0948008	ZFHX3	ZFHX3	NOS3
Ischemic stroke	C0948008	P2RY12	64805	Q9H244	purinergi	receptor		0.642	0.379	C0948008	P2RY12	P2RY12	PDE4D
Ischemic stroke	C0948008	GP6	51206	Q9HCN6	glycoprot	defense/immunity p		0.639	0.448	C0948008	GP6	GP6	PRKCH
Ischemic stroke	C0948008	HABP2	3026	Q14520	hyalurona	hydrolase; proteas		0.639	0.448	C0948008	HABP2	HABP2	ACE
Ischemic stroke	C0948008	VKORC1	79001	Q9BQB6	vitamin K	oxidoreductase		0.639	0.655	C0948008	VKORC1	VKORC1	ADORA1
Ischemic stroke	C0948008	ALOX5AP	241	P20292	arachidon	transferase		0.633	0.552	C0948008	ALOX5AP	ALOX5AP	APOA1
Ischemic stroke	C0948008	EPHX2	2053	P34913	epoxide h	hydrolase; proteas		0.633	0.448	C0948008	EPHX2	EPHX2	APOE
Ischemic stroke	C0948008	GRIN1	2902	Q05586	glutamate ionotropic recept			0.63	0.448	C0948008	GRIN1	GRIN1	BAD
Ischemic stroke	C0948008	PTGIS	5740	Q16647	prostaglandin I2 synthase			0.624	0.552	C0948008	PTGIS	PTGIS	BDNF
Ischemic stroke	C0948008	APOH	350	P02749	apolipoprotein H			0.611	0.724	C0948008	APOH	APOH	CASP3
Ischemic stroke	C0948008	FGB	2244	P02675	fibrinoge	signaling molecule		0.604	0.552	C0948008	FGB	FGB	CCL2
Ischemic stroke	C0948008	PON2	5445	Q15165	paraoxonase 2			0.596	0.621	C0948008	PON2	PON2	CD163
Ischemic stroke	C0948008	FGA	2243	P02671	fibrinogen alpha chain			0.594	0.586	C0948008	FGA	FGA	CREB1
Ischemic stroke	C0948008	CCL11	6356	P51671	C-C motif	signaling molecule		0.586	0.655	C0948008	CCL11	CCL11	CYCS
Ischemic stroke	C0948008	F8	2157	P00451	coagulati	cell adhesion mole		0.586	0.655	C0948008	F8	F8	EDN1
Ischemic stroke	C0948008	PLA2G7	7941	Q13093	phospholi	hydrolase		0.584	0.552	C0948008	PLA2G7	PLA2G7	FGA
Ischemic stroke	C0948008	PDE4D	5144	Q08499	phosphodiesterase 4D			0.572	0.69	C0948008	PDE4D	PDE4D	FOS
Ischemic stroke	C0948008	SLC1A2	6506	P43004	solute ca	transporter		0.572	0.517	C0948008	SLC1A2	SLC1A2	HBA1
Ischemic stroke	C0948008	SERPINC1	462	P01008	serpin fa	enzyme modulator		0.554	0.621	C0948008	SERPINC1	SERPINC1	IL1B
Ischemic stroke	C0948008	CDKN2B-AS1	1E+08		CDKN2B antisense RNA 1			0.55	0.586	C0948008	CDKN2B-AS1	CDKN2B-AS1	ITGA2B
Ischemic stroke	C0948008	ITGA2B	3674	P08514	integrin subunit alpha 2b			0.548	0.517	C0948008	ITGA2B	ITGA2B	LPA
Ischemic stroke	C0948008	F7	2155	P08709	coagulati	hydrolase; proteas		0.547	0.69	C0948008	F7	F7	MMP9
Ischemic stroke	C0948008	CDK5	1020	Q00535	cyclin de	kinase; transferas		0.542	0.69	C0948008	CDK5	CDK5	MTHFR
Ischemic stroke	C0948008	ADAMTS13	11093	Q76LX8	ADAM meta	enzyme modulator;		0.536	0.69	C0948008	ADAMTS13	ADAMTS13	NOS2
Ischemic stroke	C0948008	UCP2	7351	P55851	uncoupling protein 2			0.53	0.69	C0948008	UCP2	UCP2	NOTCH3
Ischemic stroke	C0948008	AQP4	361	P55087	aquaporin	transporter		0.525	0.793	C0948008	AQP4	AQP4	PARP1
Ischemic stroke	C0948008	SELP	6403	P16109	selectin P			0.519	0.724	C0948008	SELP	SELP	PLA2G7
Ischemic stroke	C0948008	HSPA1A	3303	P0DMV8;P(	heat shock protein family A			0.516	0.793	C0948008	HSPA1A	HSPA1A	PLAT
Ischemic stroke	C0948008	MMP12	4321	P39900	matrix me	hydrolase; proteas		0.516	0.862	C0948008	MMP12	MMP12	S100B
Ischemic stroke	C0948008	PITX2	5308	Q99697	paired like homeodomain 2			0.51	0.724	C0948008	PITX2	PITX2	SELE
Ischemic stroke	C0948008	ITGB3	3690	P05106	integrin	cell adhesion mole		0.507	0.69	C0948008	ITGB3	ITGB3	SERPINC1
Ischemic stroke	C0948008	THBD	7056	P07204	thrombomodulin			0.505	0.759	C0948008	THBD	THBD	SERPINE1
Ischemic stroke	C0948008	HDAC9	9734	Q9UKV0	histone deacetylase 9			0.501	0.759	C0948008	HDAC9	HDAC9	TNF
Ischemic stroke	C0948008	ABO	28	P16442	ABO, alph	transferase		0.491	0.828	C0948008	ABO	ABO	VWF
Ischemic stroke	C0948008	LPA	4018	P08519	lipoprote	hydrolase; proteas		0.489	0.655	C0948008	LPA	LPA	ABCC11
Ischemic stroke	C0948008	ALOX5	240	P09917	arachidon	oxidoreductase		0.482	0.724	C0948008	ALOX5	ALOX5	ABO

Ischemic stroke	C0948008	SELE	6401	P16581	selectin E		0.479	0.793	C0948008	SELE	SELE	ACRDYS2
Ischemic stroke	C0948008	APOB	338	P04114	apolipoprotein B		0.477	0.724	C0948008	APOB	APOB	ACSL4
Ischemic stroke	C0948008	LDLR	3949	P01130	low density lipoprotein rec		0.475	0.828	C0948008	LDLR	LDLR	ADAMTS1
Ischemic stroke	C0948008	AGER	177	Q15109	advanced	cell adhesion mole	0.472	0.724	C0948008	AGER	AGER	ADAMTS13
Ischemic stroke	C0948008	ADM	133	P35318	adrenomec	signaling molecule	0.471	0.724	C0948008	ADM	ADM	ADAMTS18
Ischemic stroke	C0948008	EDNRA	1909	P25101	endothelin receptor type A		0.469	0.828	C0948008	EDNRA	EDNRA	ADCY10
Ischemic stroke	C0948008	FBN1	2200	P35555	fibrillir	calcium-binding pr	0.469	0.69	C0948008	FBN1	FBN1	ADD3
Ischemic stroke	C0948008	NOTCH3	4854	Q9UM47	notch 3		0.469	0.69	C0948008	NOTCH3	NOTCH3	ADH1B
Ischemic stroke	C0948008	NPY	4852	P01303	neuropept	signaling molecule	0.469	0.69	C0948008	NPY	NPY	ADM
Ischemic stroke	C0948008	F5	2153	P12259	coagulati	cell adhesion mole	0.468	0.69	C0948008	F5	F5	AGER
Ischemic stroke	C0948008	TNFRSF11B	4982	000300	TNF receptor superfamily me		0.467	0.69	C0948008	TNFRSF11B	TNFRSF11B	AGT
Ischemic stroke	C0948008	F2	2147	P00734	coagulati	hydrolase; proteas	0.466	0.793	C0948008	F2	F2	AGTR1
Ischemic stroke	C0948008	VCAM1	7412	P19320	vascular cell adhesion mole		0.466	0.793	C0948008	VCAM1	VCAM1	AGTR2
Ischemic stroke	C0948008	APOA1	335	P02647	apolipoprotein A1		0.463	0.759	C0948008	APOA1	APOA1	AIF1
Ischemic stroke	C0948008	VWF	7450	P04275	von Wille	enzyme modulator	0.462	0.724	C0948008	VWF	VWF	AKT1
Ischemic stroke	C0948008	EGR1	1958	P18146	early grc	nucleic acid bindi	0.459	0.793	C0948008	EGR1	EGR1	ALB
Ischemic stroke	C0948008	PLAT	5327	P00750	plasminog	hydrolase; proteas	0.459	0.828	C0948008	PLAT	PLAT	ALOX5
Ischemic stroke	C0948008	F3	2152	P13726	coagulati	defense/immunity p	0.458	0.724	C0948008	F3	F3	ANK2
Ischemic stroke	C0948008	AGTR1	185	P30556	angiotens	receptor	0.454	0.759	C0948008	AGTR1	AGTR1	APOB
Ischemic stroke	C0948008	CD40LG	959	P29965	CD40 liga	signaling molecule	0.447	0.828	C0948008	CD40LG	CD40LG	APOBEC3G
Ischemic stroke	C0948008	PON1	5444	P27169	paraoxonase 1		0.443	0.828	C0948008	PON1	PON1	APOH
Ischemic stroke	C0948008	HSPA4	3308	P34932	heat shock protein family A		0.441	0.793	C0948008	HSPA4	HSPA4	AQP4
Ischemic stroke	C0948008	MPO	4353	P05164	myeloperc	oxidoreductase	0.435	0.931	C0948008	MPO	MPO	ASAH1
Ischemic stroke	C0948008	PLAU	5328	P00749	plasminog	hydrolase; proteas	0.434	0.69	C0948008	PLAU	PLAU	ASIC1
Ischemic stroke	C0948008	HMGB1	3146	P09429	high mobi	nucleic acid bindi	0.433	0.828	C0948008	HMGB1	HMGB1	ATF3
Ischemic stroke	C0948008	REN	5972	P00797	renin	hydrolase; proteas	0.423	0.759	C0948008	REN	REN	BBC3
Ischemic stroke	C0948008	PLG	5340	P00747	plasminog	hydrolase; proteas	0.419	0.862	C0948008	PLG	PLG	BCL2
Ischemic stroke	C0948008	MMP1	4312	P03956	matrix me	hydrolase; proteas	0.415	0.793	C0948008	MMP1	MMP1	BCO1
Ischemic stroke	C0948008	AGT	183	P01019	angiotens	enzyme modulator	0.406	0.862	C0948008	AGT	AGT	BLVRA
Ischemic stroke	C0948008	FGF2	2247	P09038	fibroblas	signaling molecule	0.404	0.897	C0948008	FGF2	FGF2	BMP7
Ischemic stroke	C0948008	IL18	3606	Q14116	interleukin 18		0.404	0.862	C0948008	IL18	IL18	C3
Ischemic stroke	C0948008	HMOX1	3162	P09601	heme oxyg	oxidoreductase	0.403	0.862	C0948008	HMOX1	HMOX1	CA1
Ischemic stroke	C0948008	NOS3	4846	P29474	nitric oxide synthase 3		0.401	0.862	C0948008	NOS3	NOS3	CA4
Ischemic stroke	C0948008	CRP	1401	P02741	C-reactive protein		0.399	0.862	C0948008	CRP	CRP	CADASIL
Ischemic stroke	C0948008	IL1RN	3557	P18510	interleukin 1 receptor anta		0.398	0.897	C0948008	IL1RN	IL1RN	CASIL
Ischemic stroke	C0948008	IL17A	3605	Q16552	interleukin 17A		0.391	0.862	C0948008	IL17A	IL17A	CASP9
Ischemic stroke	C0948008	SPP1	6696	P10451	secreted phosphoprotein 1		0.391	0.862	C0948008	SPP1	SPP1	CASZ1
Ischemic stroke	C0948008	MAPK1	5594	P28482	mitogen-a	kinase; transferas	0.387	0.862	C0948008	MAPK1	MAPK1	CAT
Ischemic stroke	C0948008	ICAM1	3383	P05362	intercellular adhesion mole		0.386	0.897	C0948008	ICAM1	ICAM1	CCL11
Ischemic stroke	C0948008	SERPINE1	5054	P05121	serpin fa	enzyme modulator	0.384	0.793	C0948008	SERPINE1	SERPINE1	CCL3
Ischemic stroke	C0948008	BDNF	627	P23560	brain der	signaling molecule	0.382	0.828	C0948008	BDNF	BDNF	CCL4
Ischemic stroke	C0948008	ACE	1636	P12821	angiotens	hydrolase; proteas	0.367	0.897	C0948008	ACE	ACE	CCL7
Ischemic stroke	C0948008	IL1A	3552	P01583	interleukin 1 alpha		0.366	0.931	C0948008	IL1A	IL1A	CCR4
Ischemic stroke	C0948008	TLR4	7099	000206	toll like receptor 4		0.359	0.931	C0948008	TLR4	TLR4	CD40LG

Ischemic stroke	C0948008	IGF1	3479	P05019	insulin like growth factor		0.355	0.828	C0948008	IGF1
Ischemic stroke	C0948008	MTHFR	4524	P42898	methylenetetrahydrofolate r		0.354	0.828	C0948008	MTHFR
Ischemic stroke	C0948008	APOE	348	P02649	apolipoprotein E		0.352	0.931	C0948008	APOE
Ischemic stroke	C0948008	MMP9	4318	P14780	matrix me	hydrolase; proteas	0.339	0.862	C0948008	MMP9
Ischemic stroke	C0948008	PTGS2	5743	P35354	prostagla	oxidoreductase	0.338	0.897	C0948008	PTGS2
Ischemic stroke	C0948008	TGFB1	7040	P01137	transform	signaling molecule	0.336	0.931	C0948008	TGFB1
Ischemic stroke	C0948008	IL1B	3553	P01584	interleukin 1 beta		0.312	0.931	C0948008	IL1B
Ischemic stroke	C0948008	VEGFA	7422	P15692	vascular	signaling molecule	0.298	0.897	C0948008	VEGFA
Ischemic stroke	C0948008	IL6	3569	P05231	interleukin 6		0.287	0.966	C0948008	IL6
Ischemic stroke	C0948008	TNF	7124	P01375	tumor nec	signaling molecule	0.263	0.966	C0948008	TNF
Ischemic stroke	C0948008	TP53	7157	P04637	tumor prc	transcription fact	0.251	0.897	C0948008	TP53

IGF1	CDK5
MTHFR	CDK6
APOE	CDKN2B-AS1
MMP9	CEBPB
PTGS2	CHRM1
TGFB1	CKB
IL1B	CNOT3
VEGFA	CNTF
IL6	COG2
TNF	CRP
TP53	CSF2
	CST3
	CTF1
	CXCL10
	CXCL2
	CXCR2
	CXCR3
	CYBB
	DCX
	DIABLO
	DPDE3
	DRD1
	DYNLL1
	EDNRA
	EGR1
	ENO2
	ENTPD1
	EPHA3
	EPHX2
	F10
	F12
	F3
	F7
	F8
	F9
	FAM107B
	FASLG
	FBN1
	FGB
	FGF2
	FGF21
	FLAP
	FOXF2
	GABRA6

GCKR
GDNF
GFAP
GIPC1
GJA1
GP6
GPR17
GPX1
GRIN1
GRIN2A
GRIN2B
GSTP1
HABP2
HDAC9
HIF1A
HMGB1
HMGCR
HMOX1
HP
HSPA1A
HSPA4
HSPA8
HSPD1
HTR4
HTRA2
ICAM1
IFNA2
IFNG
IGF1
IL10RB
IL11
IL12RB1
IL17A
IL17RA
IL18
IL1A
IL1RN
IL22
IL6
IL6ST
ILK
IMF2
IRAK1
IRAK4

IRF1
ITGAV
ITGB3
ITPR1
ITPR2
JUN
JUNB
KCNB1
KCNK10
KCNK2
KCNK3
KIAA1147
KLKB1
KMO
KRT18
KYNU
LCN2
LDLR
LRCH1
LSS
MALAT1
MAP2
MAP2K1
MAP3K5
MAPK1
MAPK3
MBP
MIAT
MMP1
MMP12
MMP8
MPO
MRAS
MROS
MT2A
MT-TL1
MYLK
NCAM1
NDRG2
NES
NFE2L2
NFKB1
NFKBIA
NGB

NGF
NKX2-5
NPY
NT5C3A
ODC1
P2RY12
PAOX
PDE3A
PDGFB
PF4
PGBD1
PIK3CA
PITX2
PKCL
PLAU
PLD1
PLG
PODXL
POMC
PON1
PON2
PPBP
PRKAA2
PRKCL
PROC
PROZ
PRPF8
PTGIS
PTGS2
PTK2B
PVALB
RB1
RELA
REN
RGS7
RHNO1
RNF213
RPRGL1
RPRGL2
RTN4
S100A5
SDC1
SELP
SERPIND1

SERPINF2
SERPINH1
SH2B3
SH3PXD2A
SIRT1
SLC11A2
SLC13A2
SLC13A5
SLC1A1
SLC1A2
SLC1A3
SLC22A12
SLC39A2
SLC8A1
SMOX
SMYD3
SOD1
SOD2
SPHK2
SPP1
SRPK2
STAT3
STRK1
SULT1A3
TAX1BP3
TBX3
TBXA2R
TFPI
TGFB1
TH
THBD
THPH1
THPH2
TIMP1
TLR4
TNFAIP6
TNFRSF11B
TNFRSF1A
TNFRSF1B
TNKS
TP53
TREM1
TSPAN2
TSPAN33

UCP2
VCAM1
VDR
VEGFA
VKORC1
WNT2B
XDH
ZCCHC14
ZFHX3

Additional file5 Shared genes of AD with IS												
Index_disease	Index_dise	Associated_disease	Associat	Gene	Gene_id	UniProt	Gene_Ful	Protein_(	pLI	DSI_g	DPI_g	diseaseic
Ischemic stroke	C0948008	Alzheimer's Disease	C0002395	NINJ2	4815	Q9NZG7	ninjurin	cell adhe	0.001235	0.834	0.103	C0948008
Ischemic stroke	C0948008	Alzheimer's Disease	C0002395	HOMER2	9455	Q9NSB8	homer sca	signaling	0.009315	0.773	0.276	C0948008
Ischemic stroke	C0948008	Alzheimer's Disease	C0002395	RCOR1	23186	Q9UKL0	REST corepressor		0.99993	0.762	0.414	C0948008
Ischemic stroke	C0948008	Alzheimer's Disease	C0002395	RNF146	81847	Q9NTX7	ring finger prote		0.84761	0.735	0.276	C0948008
Ischemic stroke	C0948008	Alzheimer's Disease	C0002395	DHX40	79665	Q8IX18	DEAH-box	nucleic a	0.74591	0.727	0.379	C0948008
Ischemic stroke	C0948008	Alzheimer's Disease	C0002395	KALRN	8997	O60229	kalirin I	signaling	1	0.72	0.207	C0948008
Ischemic stroke	C0948008	Alzheimer's Disease	C0002395	HOMER1	9456	Q86YM7	homer sca	signaling	0.99937	0.69	0.241	C0948008
Ischemic stroke	C0948008	Alzheimer's Disease	C0002395	MADD	8567	Q8WXG6	MAP kinase activa		1.20E-14	0.659	0.414	C0948008
Ischemic stroke	C0948008	Alzheimer's Disease	C0002395	ZFHX3	463	Q15911	zinc finger homeo		1	0.656	0.517	C0948008
Ischemic stroke	C0948008	Alzheimer's Disease	C0002395	F11	2160	P03951	coagulati	hydrolase	5.09E-26	0.656	0.414	C0948008
Ischemic stroke	C0948008	Alzheimer's Disease	C0002395	IRF9	10379	Q00978	interfero	nucleic a	0.5924	0.656	0.552	C0948008
Ischemic stroke	C0948008	Alzheimer's Disease	C0002395	GRM2	2912	Q14416	glutamate	receptor	0.1063	0.645	0.414	C0948008
Ischemic stroke	C0948008	Alzheimer's Disease	C0002395	CARD8	22900	Q9Y2G2	caspase recruitme		1.73E-06	0.645	0.552	C0948008
Ischemic stroke	C0948008	Alzheimer's Disease	C0002395	P2RY1	5028	P47900	purinergic recept		0.54087	0.642	0.586	C0948008
Ischemic stroke	C0948008	Alzheimer's Disease	C0002395	SERPINI1	5274	Q99574	serpin fa	enzyme mo	0.029902	0.642	0.31	C0948008
Ischemic stroke	C0948008	Alzheimer's Disease	C0002395	GRIA2	2891	P42262	glutamate ionotro		0.99906	0.639	0.414	C0948008
Ischemic stroke	C0948008	Alzheimer's Disease	C0002395	SERPINE2	5270	P07093	serpin fa	enzyme mo	0.76421	0.639	0.517	C0948008
Ischemic stroke	C0948008	Alzheimer's Disease	C0002395	PTPRG	5793	P23470	protein t	hydrolase	2.20E-05	0.639	0.69	C0948008
Ischemic stroke	C0948008	Alzheimer's Disease	C0002395	CARTPT	9607	Q16568	CART prepropeptid		0.10586	0.633	0.448	C0948008
Ischemic stroke	C0948008	Alzheimer's Disease	C0002395	GRIN1	2902	Q05586	glutamate ionotro		0.98871	0.63	0.448	C0948008
Ischemic stroke	C0948008	Alzheimer's Disease	C0002395	PLK2	10769	Q9NYY3	polo like kinase		0.9999	0.621	0.586	C0948008
Ischemic stroke	C0948008	Alzheimer's Disease	C0002395	MUT	4594	P22033	methylmal	isomerase	3.25E-21	0.616	0.655	C0948008
Ischemic stroke	C0948008	Alzheimer's Disease	C0002395	APOA5	116519	Q6Q788	apolipoprotein A5		9.05E-10	0.609	0.483	C0948008
Ischemic stroke	C0948008	Alzheimer's Disease	C0002395	PTGER2	5732	P43116	prostagla	receptor	0.041771	0.607	0.621	C0948008
Ischemic stroke	C0948008	Alzheimer's Disease	C0002395	F13A1	2162	P00488	coagulati	transfera	2.50E-07	0.602	0.586	C0948008
Ischemic stroke	C0948008	Alzheimer's Disease	C0002395	CRTC1	23373	Q6UUV9	CREB regu	transcrip	0.42733	0.602	0.621	C0948008
Ischemic stroke	C0948008	Alzheimer's Disease	C0002395	MIR424	494336		microRNA 424			0.6	0.552	C0948008
Ischemic stroke	C0948008	Alzheimer's Disease	C0002395	SRF	6722	P11831	serum res	transcrip	0.97512	0.598	0.69	C0948008
Ischemic stroke	C0948008	Alzheimer's Disease	C0002395	ALOX12	239	P18054	arachido	oxidoredu	6.61E-16	0.596	0.621	C0948008
Ischemic stroke	C0948008	Alzheimer's Disease	C0002395	PON2	5445	Q15165	paraoxonase 2		6.06E-05	0.596	0.621	C0948008
Ischemic stroke	C0948008	Alzheimer's Disease	C0002395	PADI4	23569	Q9UM07	peptidyl arginine		2.59E-16	0.592	0.586	C0948008
Ischemic stroke	C0948008	Alzheimer's Disease	C0002395	OLR1	4973	P78380	oxidized low dens		0.008896	0.59	0.552	C0948008

Ischemic stroke	C0948008	Alzheimer's Disease	C0002395	LRP6	4040	O75581	LDL receptor rela		0.98369	0.588	0.724	C0948008
Ischemic stroke	C0948008	Alzheimer's Disease	C0002395	CCL11	6356	P51671	C-C moti	signaling	0.009816	0.586	0.655	C0948008
Ischemic stroke	C0948008	Alzheimer's Disease	C0002395	MIR107	406901		microRNA 107			0.586	0.621	C0948008
Ischemic stroke	C0948008	Alzheimer's Disease	C0002395	FOLH1	2346	Q04609	folate hy	hydrolase	2.27E-08	0.584	0.724	C0948008
Ischemic stroke	C0948008	Alzheimer's Disease	C0002395	REST	5978	Q13127	RE1 silen	transcrip	0.99393	0.584	0.517	C0948008
Ischemic stroke	C0948008	Alzheimer's Disease	C0002395	PLA2G7	7941	Q13093	phosphol	hydrolase	8.88E-22	0.584	0.552	C0948008
Ischemic stroke	C0948008	Alzheimer's Disease	C0002395	DVL1	1855	O14640	dishevel	enzyme mo	1.82E-05	0.582	0.655	C0948008
Ischemic stroke	C0948008	Alzheimer's Disease	C0002395	AHSG	197	P02765	alpha 2-H	enzyme mo	2.72E-10	0.577	0.69	C0948008
Ischemic stroke	C0948008	Alzheimer's Disease	C0002395	PINK1	65018	Q9BXM7	PTEN indu	kinase;	1.03E-10	0.577	0.586	C0948008
Ischemic stroke	C0948008	Alzheimer's Disease	C0002395	SERPINA3	12	P01011	serpin fa	enzyme mo	8.07E-14	0.572	0.69	C0948008
Ischemic stroke	C0948008	Alzheimer's Disease	C0002395	CYP11B2	1585	P19099	cytochrome P450 f		9.54E-19	0.572	0.483	C0948008
Ischemic stroke	C0948008	Alzheimer's Disease	C0002395	SLC1A2	6506	P43004	solute ca	transport	0.61687	0.572	0.517	C0948008
Ischemic stroke	C0948008	Alzheimer's Disease	C0002395	TREM2	54209	Q9NZC2	triggering recept		4.00E-09	0.572	0.552	C0948008
Ischemic stroke	C0948008	Alzheimer's Disease	C0002395	PGR-AS1	1.01E+08		PGR antisense RNA 1			0.57	0.586	C0948008
Ischemic stroke	C0948008	Alzheimer's Disease	C0002395	BRS3	680	P32247	bombesin receptor		0.90963	0.569	0.69	C0948008
Ischemic stroke	C0948008	Alzheimer's Disease	C0002395	MIR132	406921		microRNA 132			0.569	0.759	C0948008
Ischemic stroke	C0948008	Alzheimer's Disease	C0002395	PC	5091	P11498	pyruvate	ligase	0.007175	0.565	0.724	C0948008
Ischemic stroke	C0948008	Alzheimer's Disease	C0002395	A2M	2	P01023	alpha-2-m	defense/i	5.61E-11	0.564	0.724	C0948008
Ischemic stroke	C0948008	Alzheimer's Disease	C0002395	PTGES	9536	O14684	prostaglandin E s		0.65991	0.562	0.69	C0948008
Ischemic stroke	C0948008	Alzheimer's Disease	C0002395	MIR29B1	407024		microRNA 29b-1			0.558	0.69	C0948008
Ischemic stroke	C0948008	Alzheimer's Disease	C0002395	C20orf181	1E+08		chromosome 20 open reading			0.557	0.69	C0948008
Ischemic stroke	C0948008	Alzheimer's Disease	C0002395	CETP	1071	P11597	cholesteryl ester		1.03E-23	0.555	0.655	C0948008
Ischemic stroke	C0948008	Alzheimer's Disease	C0002395	SERPINC1	462	P01008	serpin fa	enzyme mo	0.99863	0.554	0.621	C0948008
Ischemic stroke	C0948008	Alzheimer's Disease	C0002395	MOK	5891	Q9UQ07	MOK prote	kinase;	9.92E-22	0.554	0.655	C0948008
Ischemic stroke	C0948008	Alzheimer's Disease	C0002395	MIR29B2	407025		microRNA 29b-2			0.554	0.724	C0948008
Ischemic stroke	C0948008	Alzheimer's Disease	C0002395	SLC33A1	9197	O00400	solute ca	transport	9.14E-05	0.551	0.655	C0948008
Ischemic stroke	C0948008	Alzheimer's Disease	C0002395	FLG	2312	P20930	filaggrin		0.000312	0.55	0.655	C0948008
Ischemic stroke	C0948008	Alzheimer's Disease	C0002395	CDKN2B-AS1	1E+08		CDKN2B antisense RNA 1			0.55	0.586	C0948008
Ischemic stroke	C0948008	Alzheimer's Disease	C0002395	F7	2155	P08709	coagulati	hydrolase	1.53E-05	0.547	0.69	C0948008
Ischemic stroke	C0948008	Alzheimer's Disease	C0002395	NPPA	4878	P01160	natriuretic pepti		0.000639	0.546	0.69	C0948008
Ischemic stroke	C0948008	Alzheimer's Disease	C0002395	TFPI2	7980	P48307	tissue fa	enzyme mo	0.00078	0.545	0.621	C0948008
Ischemic stroke	C0948008	Alzheimer's Disease	C0002395	NR4A3	8013	Q92570	nuclear r	receptor	0.89536	0.545	0.759	C0948008
Ischemic stroke	C0948008	Alzheimer's Disease	C0002395	CDK5	1020	Q00535	cyclin de	kinase;	0.095196	0.542	0.69	C0948008
Ischemic stroke	C0948008	Alzheimer's Disease	C0002395	CYP2C9	1559	P11712	cytochrome P450 f		1.80E-10	0.542	0.759	C0948008
Ischemic stroke	C0948008	Alzheimer's Disease	C0002395	PPIG	9360	Q13427	peptidylprolyl is		0.9649	0.537	0.793	C0948008
Ischemic stroke	C0948008	Alzheimer's Disease	C0002395	PIN1	5300	Q13526	peptidylprolyl ci		0.76129	0.536	0.655	C0948008

Ischemic stroke	C0948008	Alzheimer's Disease	C0002395	GNB3	2784	P16520	G protei	enzyme mo	2.92E-11	0.533	0.724	C0948008
Ischemic stroke	C0948008	Alzheimer's Disease	C0002395	LRP1	4035	Q07954	LDL receptor rela		1	0.533	0.724	C0948008
Ischemic stroke	C0948008	Alzheimer's Disease	C0002395	IL9	3578	P15248	interleukin 9		9.62E-07	0.53	0.69	C0948008
Ischemic stroke	C0948008	Alzheimer's Disease	C0002395	LIF	3976	P15018	LIF, inte	signaling	0.50007	0.528	0.655	C0948008
Ischemic stroke	C0948008	Alzheimer's Disease	C0002395	MSC	9242	O60682	musculin		0.022585	0.526	0.655	C0948008
Ischemic stroke	C0948008	Alzheimer's Disease	C0002395	AQP4	361	P55087	aquapori	transpor	0.002775	0.525	0.793	C0948008
Ischemic stroke	C0948008	Alzheimer's Disease	C0002395	KCNQ1	3784	P51787	potassium voltage		2.07E-07	0.525	0.586	C0948008
Ischemic stroke	C0948008	Alzheimer's Disease	C0002395	NTF3	4908	P20783	neurotro	signaling	0.88635	0.525	0.621	C0948008
Ischemic stroke	C0948008	Alzheimer's Disease	C0002395	NR3C2	4306	P08235	nuclear	nucleic a	0.80843	0.52	0.69	C0948008
Ischemic stroke	C0948008	Alzheimer's Disease	C0002395	HSPA1A	3303	P0DMV8;P(	heat shock protei		0.029335	0.516	0.793	C0948008
Ischemic stroke	C0948008	Alzheimer's Disease	C0002395	HSPA1B	3304	P0DMV8;P(	heat shock protei		0.005091	0.514	0.793	C0948008
Ischemic stroke	C0948008	Alzheimer's Disease	C0002395	EEF1A2	1917	Q05639	eukaryoti	enzyme mo	0.99606	0.51	0.69	C0948008
Ischemic stroke	C0948008	Alzheimer's Disease	C0002395	MTRR	4552	Q9UBK8	5-methyltetrahydr		1.77E-14	0.51	0.655	C0948008
Ischemic stroke	C0948008	Alzheimer's Disease	C0002395	PLA2G2A	5320	P14555	phosphol	hydrolase	0.10723	0.509	0.69	C0948008
Ischemic stroke	C0948008	Alzheimer's Disease	C0002395	ITGB3	3690	P05106	integrin	cell adhe	0.002193	0.507	0.69	C0948008
Ischemic stroke	C0948008	Alzheimer's Disease	C0002395	NRG1	3084	Q02297	neuregul	signaling	0.99553	0.506	0.759	C0948008
Ischemic stroke	C0948008	Alzheimer's Disease	C0002395	LPL	4023	P06858	lipoprot	hydrolase	7.23E-10	0.504	0.724	C0948008
Ischemic stroke	C0948008	Alzheimer's Disease	C0002395	P2RX7	5027	Q99572	purinerg	receptor	3.05E-11	0.503	0.759	C0948008
Ischemic stroke	C0948008	Alzheimer's Disease	C0002395	CHI3L1	1116	P36222	chitinase 3 like		8.51E-06	0.502	0.793	C0948008
Ischemic stroke	C0948008	Alzheimer's Disease	C0002395	PLA2G1B	5319	P04054	phosphol	hydrolase	1.09E-10	0.502	0.793	C0948008
Ischemic stroke	C0948008	Alzheimer's Disease	C0002395	CYP2C19	1557	P33261	cytochrome P450 f		4.11E-20	0.501	0.793	C0948008
Ischemic stroke	C0948008	Alzheimer's Disease	C0002395	HDAC9	9734	Q9UKV0	histone deacetyla		0.99996	0.501	0.759	C0948008
Ischemic stroke	C0948008	Alzheimer's Disease	C0002395	GDF15	9518	Q99988	growth d	signaling	2.85E-07	0.496	0.621	C0948008
Ischemic stroke	C0948008	Alzheimer's Disease	C0002395	PTGS1	5742	P23219	prostagl	oxidoredu	2.65E-06	0.495	0.828	C0948008
Ischemic stroke	C0948008	Alzheimer's Disease	C0002395	PLA2G6	8398	O60733	phosphol	enzyme mo	2.79E-10	0.494	0.724	C0948008
Ischemic stroke	C0948008	Alzheimer's Disease	C0002395	MTR	4548	Q99707	5-methyltetrahydr		2.65E-10	0.493	0.724	C0948008
Ischemic stroke	C0948008	Alzheimer's Disease	C0002395	PECAM1	5175	P16284	platelet	cell adhesion mol		0.492	0.69	C0948008
Ischemic stroke	C0948008	Alzheimer's Disease	C0002395	ABO	28	P16442	ABO, alp	transferase		0.491	0.828	C0948008
Ischemic stroke	C0948008	Alzheimer's Disease	C0002395	LPA	4018	P08519	lipoprot	hydrolase	5.81E-88	0.489	0.655	C0948008
Ischemic stroke	C0948008	Alzheimer's Disease	C0002395	AVP	551	P01185	arginine	signaling	0.075195	0.486	0.759	C0948008
Ischemic stroke	C0948008	Alzheimer's Disease	C0002395	ABCA1	19	O95477	ATP bind	transpor	3.53E-12	0.484	0.793	C0948008
Ischemic stroke	C0948008	Alzheimer's Disease	C0002395	ALOX5	240	P09917	arachido	oxidoredu	1.50E-06	0.482	0.724	C0948008
Ischemic stroke	C0948008	Alzheimer's Disease	C0002395	KCNA3	3738	P22001	potassium voltage		0.86034	0.481	0.724	C0948008
Ischemic stroke	C0948008	Alzheimer's Disease	C0002395	ADRB2	154	P07550	adrenoce	receptor	0.58042	0.479	0.793	C0948008
Ischemic stroke	C0948008	Alzheimer's Disease	C0002395	ANGPT2	285	O15123	angiopoi	signaling	0.85572	0.479	0.724	C0948008
Ischemic stroke	C0948008	Alzheimer's Disease	C0002395	BIRC5	332	O15392	baculovi	enzyme mo	0.056738	0.479	0.655	C0948008

Ischemic stroke	C0948008	Alzheimer's Disease	C0002395	F2R	2149	P25116	coagulation facto		0.00577	0.478	0.759	C0948008
Ischemic stroke	C0948008	Alzheimer's Disease	C0002395	CFH	3075	P08603	complement factor		0.96239	0.478	0.793	C0948008
Ischemic stroke	C0948008	Alzheimer's Disease	C0002395	APOB	338	P04114	apolipoprotein B		1.64E-16	0.477	0.724	C0948008
Ischemic stroke	C0948008	Alzheimer's Disease	C0002395	OGG1	4968	O15527	8-oxoguanine DNA		2.14E-12	0.476	0.759	C0948008
Ischemic stroke	C0948008	Alzheimer's Disease	C0002395	LDLR	3949	P01130	low density lipop		9.44E-24	0.475	0.828	C0948008
Ischemic stroke	C0948008	Alzheimer's Disease	C0002395	PRNP	5621	F7VJQ1;P(	prion protein		0.000715	0.474	0.897	C0948008
Ischemic stroke	C0948008	Alzheimer's Disease	C0002395	AGER	177	Q15109	advanced	cell adh	3.44E-16	0.472	0.724	C0948008
Ischemic stroke	C0948008	Alzheimer's Disease	C0002395	ADM	133	P35318	adrenome	signalin	0.034615	0.471	0.724	C0948008
Ischemic stroke	C0948008	Alzheimer's Disease	C0002395	NPY	4852	P01303	neuropep	signalin	0.15508	0.469	0.69	C0948008
Ischemic stroke	C0948008	Alzheimer's Disease	C0002395	NOTCH3	4854	Q9UM47	notch 3		0.86965	0.469	0.69	C0948008
Ischemic stroke	C0948008	Alzheimer's Disease	C0002395	SEMA6A	57556	Q9H2E6	semaphor	signalin	0.99993	0.468	0.793	C0948008
Ischemic stroke	C0948008	Alzheimer's Disease	C0002395	NQO1	1728	P15559	NAD(P)H quinone d		1.02E-09	0.467	0.724	C0948008
Ischemic stroke	C0948008	Alzheimer's Disease	C0002395	TIMP2	7077	P16035	TIMP met	enzyme m	0.80818	0.467	0.759	C0948008
Ischemic stroke	C0948008	Alzheimer's Disease	C0002395	F2	2147	P00734	coagulat	hydrolas	0.001125	0.466	0.793	C0948008
Ischemic stroke	C0948008	Alzheimer's Disease	C0002395	IFNB1	3456	P01574	interferon beta 1			0.466	0.793	C0948008
Ischemic stroke	C0948008	Alzheimer's Disease	C0002395	VCAM1	7412	P19320	vascular cell adh		0.74466	0.466	0.793	C0948008
Ischemic stroke	C0948008	Alzheimer's Disease	C0002395	PPARA	5465	Q07869	peroxiso	nucleic	0.037307	0.465	0.793	C0948008
Ischemic stroke	C0948008	Alzheimer's Disease	C0002395	APOA1	335	P02647	apolipoprotein A1		0.000549	0.463	0.759	C0948008
Ischemic stroke	C0948008	Alzheimer's Disease	C0002395	VWF	7450	P04275	von Wille	enzyme m	7.85E-24	0.462	0.724	C0948008
Ischemic stroke	C0948008	Alzheimer's Disease	C0002395	COX8A	1351	P10176	cytochro	oxidored	0.10544	0.461	0.793	C0948008
Ischemic stroke	C0948008	Alzheimer's Disease	C0002395	EGR1	1958	P18146	early gr	nucleic	0.3269	0.459	0.793	C0948008
Ischemic stroke	C0948008	Alzheimer's Disease	C0002395	PLAT	5327	P00750	plasmino	hydrolas	3.46E-05	0.459	0.828	C0948008
Ischemic stroke	C0948008	Alzheimer's Disease	C0002395	AGTR1	185	P30556	angioten	receptor	0.000572	0.454	0.759	C0948008
Ischemic stroke	C0948008	Alzheimer's Disease	C0002395	MIR146A	406938		microRNA 146a			0.452	0.759	C0948008
Ischemic stroke	C0948008	Alzheimer's Disease	C0002395	XRCC1	7515	P18887	X-ray repair cros		4.76E-09	0.449	0.828	C0948008
Ischemic stroke	C0948008	Alzheimer's Disease	C0002395	ABCG2	9429	Q9UNQ0	ATP bind	transpor	1.26E-32	0.449	0.793	C0948008
Ischemic stroke	C0948008	Alzheimer's Disease	C0002395	CD40LG	959	P29965	CD40 lig	signalin	0.72669	0.447	0.828	C0948008
Ischemic stroke	C0948008	Alzheimer's Disease	C0002395	ESR2	2100	Q92731	estrogen	nucleic	5.39E-08	0.445	0.828	C0948008
Ischemic stroke	C0948008	Alzheimer's Disease	C0002395	PON1	5444	P27169	paraoxonase 1		8.98E-11	0.443	0.828	C0948008
Ischemic stroke	C0948008	Alzheimer's Disease	C0002395	HSPA4	3308	P34932	heat shock protei		0.9999	0.441	0.793	C0948008
Ischemic stroke	C0948008	Alzheimer's Disease	C0002395	HFE	3077	Q30201	homeostatic iron		3.01E-08	0.44	0.69	C0948008
Ischemic stroke	C0948008	Alzheimer's Disease	C0002395	MMP3	4314	P08254	matrix m	hydrolas	4.93E-15	0.439	0.793	C0948008
Ischemic stroke	C0948008	Alzheimer's Disease	C0002395	CYP2B6	1555	P20813	cytochrome P450 f		3.50E-10	0.437	0.828	C0948008
Ischemic stroke	C0948008	Alzheimer's Disease	C0002395	MPO	4353	P05164	myeloper	oxidored	6.82E-15	0.435	0.931	C0948008
Ischemic stroke	C0948008	Alzheimer's Disease	C0002395	PLAU	5328	P00749	plasmino	hydrolas	2.14E-06	0.434	0.69	C0948008
Ischemic stroke	C0948008	Alzheimer's Disease	C0002395	CD40	958	P25942	CD40 molecule		0.85939	0.433	0.862	C0948008

Ischemic stroke	C0948008	Alzheimer's Disease	C0002395	HMGB1	3146	P09429	high mob	nucleic a	0.83018	0.433	0.828	C0948008
Ischemic stroke	C0948008	Alzheimer's Disease	C0002395	IGF1R	3480	P08069	insulin like grow		0.98751	0.433	0.828	C0948008
Ischemic stroke	C0948008	Alzheimer's Disease	C0002395	BRCA2	675	P51587	BRCA2, D	nucleic a	5.46E-24	0.426	0.793	C0948008
Ischemic stroke	C0948008	Alzheimer's Disease	C0002395	COMT	1312	P21964	catechol-	transfera	1.27E-05	0.426	0.897	C0948008
Ischemic stroke	C0948008	Alzheimer's Disease	C0002395	EPHB2	2048	P29323	EPH receptor B2		0.99999	0.425	0.759	C0948008
Ischemic stroke	C0948008	Alzheimer's Disease	C0002395	EPO	2056	P01588	erythropoietin		0.012147	0.425	0.759	C0948008
Ischemic stroke	C0948008	Alzheimer's Disease	C0002395	REN	5972	P00797	renin	hydrolase	2.12E-07	0.423	0.759	C0948008
Ischemic stroke	C0948008	Alzheimer's Disease	C0002395	CSF3	1440	P09919	colony stimulatin		0.32663	0.421	0.828	C0948008
Ischemic stroke	C0948008	Alzheimer's Disease	C0002395	EZH2	2146	Q15910	enhancer of zeste		1	0.421	0.759	C0948008
Ischemic stroke	C0948008	Alzheimer's Disease	C0002395	PLG	5340	P00747	plasmino	hydrolase	0.009642	0.419	0.862	C0948008
Ischemic stroke	C0948008	Alzheimer's Disease	C0002395	MMP1	4312	P03956	matrix me	hydrolase	6.24E-18	0.415	0.793	C0948008
Ischemic stroke	C0948008	Alzheimer's Disease	C0002395	NOTCH1	4851	P46531	notch 1		1	0.415	0.759	C0948008
Ischemic stroke	C0948008	Alzheimer's Disease	C0002395	AGT	183	P01019	angiotens	enzyme mo	3.87E-08	0.406	0.862	C0948008
Ischemic stroke	C0948008	Alzheimer's Disease	C0002395	FGF2	2247	P09038	fibroblas	signaling	0.019149	0.404	0.897	C0948008
Ischemic stroke	C0948008	Alzheimer's Disease	C0002395	IL18	3606	Q14116	interleukin 18		0.029184	0.404	0.862	C0948008
Ischemic stroke	C0948008	Alzheimer's Disease	C0002395	HMOX1	3162	P09601	heme oxyg	oxidoredu	0.007886	0.403	0.862	C0948008
Ischemic stroke	C0948008	Alzheimer's Disease	C0002395	ATM	472	Q13315	ATM seri	kinase; r	5.98E-46	0.401	0.862	C0948008
Ischemic stroke	C0948008	Alzheimer's Disease	C0002395	NOS3	4846	P29474	nitric oxide synt		8.89E-07	0.401	0.862	C0948008
Ischemic stroke	C0948008	Alzheimer's Disease	C0002395	CRP	1401	P02741	C-reactive protei		0.003736	0.399	0.862	C0948008
Ischemic stroke	C0948008	Alzheimer's Disease	C0002395	IL1RN	3557	P18510	interleukin 1 rec		0.033509	0.398	0.897	C0948008
Ischemic stroke	C0948008	Alzheimer's Disease	C0002395	COX2	4513	P00403	cytochrom	oxidoreductase		0.397	0.862	C0948008
Ischemic stroke	C0948008	Alzheimer's Disease	C0002395	IL17A	3605	Q16552	interleukin 17A		0.044037	0.391	0.862	C0948008
Ischemic stroke	C0948008	Alzheimer's Disease	C0002395	PPARG	5468	P37231	peroxison	nucleic a	0.1447	0.388	0.828	C0948008
Ischemic stroke	C0948008	Alzheimer's Disease	C0002395	MAPK1	5594	P28482	mitogen-a	kinase; t	0.9973	0.387	0.862	C0948008
Ischemic stroke	C0948008	Alzheimer's Disease	C0002395	ICAM1	3383	P05362	intercellular adh		0.038102	0.386	0.897	C0948008
Ischemic stroke	C0948008	Alzheimer's Disease	C0002395	SERPINE1	5054	P05121	serpin fa	enzyme mo	0.047491	0.384	0.793	C0948008
Ischemic stroke	C0948008	Alzheimer's Disease	C0002395	BDNF	627	P23560	brain der	signaling	0.69969	0.382	0.828	C0948008
Ischemic stroke	C0948008	Alzheimer's Disease	C0002395	AR	367	P10275	androgen	nucleic a	0.98299	0.38	0.793	C0948008
Ischemic stroke	C0948008	Alzheimer's Disease	C0002395	MMP2	4313	P08253	matrix me	hydrolase	0.83952	0.373	0.862	C0948008
Ischemic stroke	C0948008	Alzheimer's Disease	C0002395	ABCB1	5243	P08183	ATP bind	hydrolase	1.31E-05	0.371	0.828	C0948008
Ischemic stroke	C0948008	Alzheimer's Disease	C0002395	ACE	1636	P12821	angiotens	hydrolase	1.55E-37	0.367	0.897	C0948008
Ischemic stroke	C0948008	Alzheimer's Disease	C0002395	IL1A	3552	P01583	interleukin 1 alp		0.000149	0.366	0.931	C0948008
Ischemic stroke	C0948008	Alzheimer's Disease	C0002395	IL4	3565	P05112	interleukin 4		0.005296	0.364	0.931	C0948008
Ischemic stroke	C0948008	Alzheimer's Disease	C0002395	TLR4	7099	O00206	toll like recepto		5.58E-09	0.359	0.931	C0948008
Ischemic stroke	C0948008	Alzheimer's Disease	C0002395	ESR1	2099	P03372	estrogen	nucleic a	0.9994	0.355	0.897	C0948008
Ischemic stroke	C0948008	Alzheimer's Disease	C0002395	IGF1	3479	P05019	insulin like grow		0.28843	0.355	0.828	C0948008

Ischemic stroke	C0948008	Alzheimer's Disease	C0002395	MTHFR	4524	P42898	methylenetetrahyd		1.22E-09	0.354	0.828	C0948008
Ischemic stroke	C0948008	Alzheimer's Disease	C0002395	APOE	348	P02649	apolipoprotein E		0.001737	0.352	0.931	C0948008
Ischemic stroke	C0948008	Alzheimer's Disease	C0002395	CXCL8	3576	P10145	C-X-C mo	signaling molecul		0.342	0.862	C0948008
Ischemic stroke	C0948008	Alzheimer's Disease	C0002395	MMP9	4318	P14780	matrix me	hydrolase	7.03E-18	0.339	0.862	C0948008
Ischemic stroke	C0948008	Alzheimer's Disease	C0002395	PTGS2	5743	P35354	prostagla	oxidoredu	0.99639	0.338	0.897	C0948008
Ischemic stroke	C0948008	Alzheimer's Disease	C0002395	TGFB1	7040	P01137	transform	signaling	0.17182	0.336	0.931	C0948008
Ischemic stroke	C0948008	Alzheimer's Disease	C0002395	CDKN2A	1029	P42771;Q8	cyclin dependent		0.38138	0.321	0.828	C0948008
Ischemic stroke	C0948008	Alzheimer's Disease	C0002395	IL10	3586	P22301	interleukin 10		0.006506	0.32	0.931	C0948008
Ischemic stroke	C0948008	Alzheimer's Disease	C0002395	IL1B	3553	P01584	interleukin 1 bet		0.12568	0.312	0.931	C0948008
Ischemic stroke	C0948008	Alzheimer's Disease	C0002395	VEGFA	7422	P15692	vascular	signaling	2.65E-05	0.298	0.897	C0948008
Ischemic stroke	C0948008	Alzheimer's Disease	C0002395	IL6	3569	P05231	interleukin 6		0.33873	0.287	0.966	C0948008
Ischemic stroke	C0948008	Alzheimer's Disease	C0002395	TNF	7124	P01375	tumor nec	signaling	0.8046	0.263	0.966	C0948008
Ischemic stroke	C0948008	Alzheimer's Disease	C0002395	TP53	7157	P04637	tumor pro	transcrip	0.16862	0.251	0.897	C0948008

Additional file5 Shared genes of ALS with IS												
Index_disease	Index_di	Associated_disease	Associat	Gene	Gene_id	UniProt	Gene_Ful	Protein_(	pLI	DSI_g	DPI_g	diseaseic
Ischemic stroke	C0948008	Amyotrophic Lateral Sclerosis	C0002736	GRM2	2912	Q14416	glutamat	receptor	0.1063	0.645	0.414	C0948008
Ischemic stroke	C0948008	Amyotrophic Lateral Sclerosis	C0002736	COL4A2	1284	P08572	collagen type IV		1.04E-13	0.642	0.448	C0948008
Ischemic stroke	C0948008	Amyotrophic Lateral Sclerosis	C0002736	P2RY1	5028	P47900	purinergic recept		0.54087	0.642	0.586	C0948008
Ischemic stroke	C0948008	Amyotrophic Lateral Sclerosis	C0002736	MASP2	10747	O00187	mannan b	hydrolase	3.59E-14	0.642	0.655	C0948008
Ischemic stroke	C0948008	Amyotrophic Lateral Sclerosis	C0002736	GRIA2	2891	P42262	glutamate ionotro		0.99906	0.639	0.414	C0948008
Ischemic stroke	C0948008	Amyotrophic Lateral Sclerosis	C0002736	PTGER2	5732	P43116	prostagl	receptor	0.041771	0.607	0.621	C0948008
Ischemic stroke	C0948008	Amyotrophic Lateral Sclerosis	C0002736	PON2	5445	Q15165	paraoxonase 2		6.06E-05	0.596	0.621	C0948008
Ischemic stroke	C0948008	Amyotrophic Lateral Sclerosis	C0002736	TRPM7	54822	Q96QT4	transien	receptor	1.72E-15	0.584	0.552	C0948008
Ischemic stroke	C0948008	Amyotrophic Lateral Sclerosis	C0002736	TNFSF14	8740	O43557	TNF supe	signaling	0.046389	0.58	0.586	C0948008
Ischemic stroke	C0948008	Amyotrophic Lateral Sclerosis	C0002736	PINK1	65018	Q9BXM7	PTEN ind	kinase;	1.03E-10	0.577	0.586	C0948008
Ischemic stroke	C0948008	Amyotrophic Lateral Sclerosis	C0002736	GPX3	2878	P22352	glutathi	oxidoredu	0.042528	0.575	0.586	C0948008
Ischemic stroke	C0948008	Amyotrophic Lateral Sclerosis	C0002736	SLC1A2	6506	P43004	solute ca	transpor	0.61687	0.572	0.517	C0948008
Ischemic stroke	C0948008	Amyotrophic Lateral Sclerosis	C0002736	TREM2	54209	Q9NZC2	triggering recept		4.00E-09	0.572	0.552	C0948008
Ischemic stroke	C0948008	Amyotrophic Lateral Sclerosis	C0002736	PTGES	9536	O14684	prostaglandin E s		0.65991	0.562	0.69	C0948008
Ischemic stroke	C0948008	Amyotrophic Lateral Sclerosis	C0002736	SLC33A1	9197	O00400	solute ca	transpor	9.14E-05	0.551	0.655	C0948008
Ischemic stroke	C0948008	Amyotrophic Lateral Sclerosis	C0002736	TFPI2	7980	P48307	tissue fa	enzyme mo	0.00078	0.545	0.621	C0948008
Ischemic stroke	C0948008	Amyotrophic Lateral Sclerosis	C0002736	CDK5	1020	Q00535	cyclin de	kinase;	0.095196	0.542	0.69	C0948008
Ischemic stroke	C0948008	Amyotrophic Lateral Sclerosis	C0002736	SULT1E1	6783	P49888	sulfotransferase		2.17E-07	0.538	0.759	C0948008
Ischemic stroke	C0948008	Amyotrophic Lateral Sclerosis	C0002736	PIN1	5300	Q13526	peptidylprolyl ci		0.76129	0.536	0.655	C0948008
Ischemic stroke	C0948008	Amyotrophic Lateral Sclerosis	C0002736	SGK1	6446	O00141	serum/glu	calcium-b	0.040926	0.53	0.621	C0948008
Ischemic stroke	C0948008	Amyotrophic Lateral Sclerosis	C0002736	UCP2	7351	P55851	uncoupling protei		1.35E-10	0.53	0.69	C0948008
Ischemic stroke	C0948008	Amyotrophic Lateral Sclerosis	C0002736	LIF	3976	P15018	LIF, int	signaling	0.50007	0.528	0.655	C0948008
Ischemic stroke	C0948008	Amyotrophic Lateral Sclerosis	C0002736	MSC	9242	O60682	musculin		0.022585	0.526	0.655	C0948008
Ischemic stroke	C0948008	Amyotrophic Lateral Sclerosis	C0002736	AQP4	361	P55087	aquaporin	transpor	0.002775	0.525	0.793	C0948008
Ischemic stroke	C0948008	Amyotrophic Lateral Sclerosis	C0002736	NTF3	4908	P20783	neurotrop	signaling	0.88635	0.525	0.621	C0948008
Ischemic stroke	C0948008	Amyotrophic Lateral Sclerosis	C0002736	NRG1	3084	Q02297	neuregul	signaling	0.99553	0.506	0.759	C0948008
Ischemic stroke	C0948008	Amyotrophic Lateral Sclerosis	C0002736	P2RX7	5027	Q99572	purinergi	receptor	3.05E-11	0.503	0.759	C0948008
Ischemic stroke	C0948008	Amyotrophic Lateral Sclerosis	C0002736	ALOX5	240	P09917	arachidon	oxidoredu	1.50E-06	0.482	0.724	C0948008
Ischemic stroke	C0948008	Amyotrophic Lateral Sclerosis	C0002736	KCNA3	3738	P22001	potassium voltage		0.86034	0.481	0.724	C0948008
Ischemic stroke	C0948008	Amyotrophic Lateral Sclerosis	C0002736	ENG	2022	P17813	endoglin		0.99877	0.477	0.655	C0948008
Ischemic stroke	C0948008	Amyotrophic Lateral Sclerosis	C0002736	PRNP	5621	F7VJQ1;P	prion protein		0.000715	0.474	0.897	C0948008
Ischemic stroke	C0948008	Amyotrophic Lateral Sclerosis	C0002736	TIMP2	7077	P16035	TIMP meta	enzyme mo	0.80818	0.467	0.759	C0948008

Ischemic stroke	C0948008	Amyotrophic Lateral Sclerosis	C0002736	COX8A	1351	P10176	cytochrom	oxidoredu	0.10544	0.461	0.793	C0948008
Ischemic stroke	C0948008	Amyotrophic Lateral Sclerosis	C0002736	MIR146A	406938		microRNA 146a			0.452	0.759	C0948008
Ischemic stroke	C0948008	Amyotrophic Lateral Sclerosis	C0002736	XRCC1	7515	P18887	X-ray repair cros		4.76E-09	0.449	0.828	C0948008
Ischemic stroke	C0948008	Amyotrophic Lateral Sclerosis	C0002736	CD40LG	959	P29965	CD40 liga	signaling	0.72669	0.447	0.828	C0948008
Ischemic stroke	C0948008	Amyotrophic Lateral Sclerosis	C0002736	PON1	5444	P27169	paraoxonase 1		8.98E-11	0.443	0.828	C0948008
Ischemic stroke	C0948008	Amyotrophic Lateral Sclerosis	C0002736	HSPA4	3308	P34932	heat shock protei		0.9999	0.441	0.793	C0948008
Ischemic stroke	C0948008	Amyotrophic Lateral Sclerosis	C0002736	HFE	3077	Q30201	homeostatic iron		3.01E-08	0.44	0.69	C0948008
Ischemic stroke	C0948008	Amyotrophic Lateral Sclerosis	C0002736	IGF1R	3480	P08069	insulin like grow		0.98751	0.433	0.828	C0948008
Ischemic stroke	C0948008	Amyotrophic Lateral Sclerosis	C0002736	EPHB2	2048	P29323	EPH receptor B2		0.99999	0.425	0.759	C0948008
Ischemic stroke	C0948008	Amyotrophic Lateral Sclerosis	C0002736	EPO	2056	P01588	erythropoietin		0.012147	0.425	0.759	C0948008
Ischemic stroke	C0948008	Amyotrophic Lateral Sclerosis	C0002736	KDR	3791	P35968	kinase insert dom		0.99979	0.423	0.724	C0948008
Ischemic stroke	C0948008	Amyotrophic Lateral Sclerosis	C0002736	CSF3	1440	P09919	colony stimulatin		0.32663	0.421	0.828	C0948008
Ischemic stroke	C0948008	Amyotrophic Lateral Sclerosis	C0002736	NOTCH1	4851	P46531	notch 1		1	0.415	0.759	C0948008
Ischemic stroke	C0948008	Amyotrophic Lateral Sclerosis	C0002736	FGF2	2247	P09038	fibrobla	signaling	0.019149	0.404	0.897	C0948008
Ischemic stroke	C0948008	Amyotrophic Lateral Sclerosis	C0002736	HMOX1	3162	P09601	heme oxy	oxidoredu	0.007886	0.403	0.862	C0948008
Ischemic stroke	C0948008	Amyotrophic Lateral Sclerosis	C0002736	CRP	1401	P02741	C-reactive protei		0.003736	0.399	0.862	C0948008
Ischemic stroke	C0948008	Amyotrophic Lateral Sclerosis	C0002736	COX2	4513	P00403	cytochrom	oxidoreductase		0.397	0.862	C0948008
Ischemic stroke	C0948008	Amyotrophic Lateral Sclerosis	C0002736	IL17A	3605	Q16552	interleukin 17A		0.044037	0.391	0.862	C0948008
Ischemic stroke	C0948008	Amyotrophic Lateral Sclerosis	C0002736	PPARG	5468	P37231	peroxisor	nucleic a	0.1447	0.388	0.828	C0948008
Ischemic stroke	C0948008	Amyotrophic Lateral Sclerosis	C0002736	MAPK1	5594	P28482	mitogen-a	kinase;	0.9973	0.387	0.862	C0948008
Ischemic stroke	C0948008	Amyotrophic Lateral Sclerosis	C0002736	BDNF	627	P23560	brain de	signaling	0.69969	0.382	0.828	C0948008
Ischemic stroke	C0948008	Amyotrophic Lateral Sclerosis	C0002736	AR	367	P10275	androgen	nucleic a	0.98299	0.38	0.793	C0948008
Ischemic stroke	C0948008	Amyotrophic Lateral Sclerosis	C0002736	IL4	3565	P05112	interleukin 4		0.005296	0.364	0.931	C0948008
Ischemic stroke	C0948008	Amyotrophic Lateral Sclerosis	C0002736	TLR4	7099	O00206	toll like recepto		5.58E-09	0.359	0.931	C0948008
Ischemic stroke	C0948008	Amyotrophic Lateral Sclerosis	C0002736	IGF1	3479	P05019	insulin like grow		0.28843	0.355	0.828	C0948008
Ischemic stroke	C0948008	Amyotrophic Lateral Sclerosis	C0002736	APOE	348	P02649	apolipoprotein E		0.001737	0.352	0.931	C0948008
Ischemic stroke	C0948008	Amyotrophic Lateral Sclerosis	C0002736	MMP9	4318	P14780	matrix me	hydrolase	7.03E-18	0.339	0.862	C0948008
Ischemic stroke	C0948008	Amyotrophic Lateral Sclerosis	C0002736	PTGS2	5743	P35354	prostagla	oxidoredu	0.99639	0.338	0.897	C0948008
Ischemic stroke	C0948008	Amyotrophic Lateral Sclerosis	C0002736	TGFB1	7040	P01137	transforr	signaling	0.17182	0.336	0.931	C0948008
Ischemic stroke	C0948008	Amyotrophic Lateral Sclerosis	C0002736	CDKN2A	1029	P42771;Q8	cyclin dependent		0.38138	0.321	0.828	C0948008
Ischemic stroke	C0948008	Amyotrophic Lateral Sclerosis	C0002736	IL10	3586	P22301	interleukin 10		0.006506	0.32	0.931	C0948008
Ischemic stroke	C0948008	Amyotrophic Lateral Sclerosis	C0002736	IL1B	3553	P01584	interleukin 1 bet		0.12568	0.312	0.931	C0948008
Ischemic stroke	C0948008	Amyotrophic Lateral Sclerosis	C0002736	VEGFA	7422	P15692	vascular	signaling	2.65E-05	0.298	0.897	C0948008
Ischemic stroke	C0948008	Amyotrophic Lateral Sclerosis	C0002736	TNF	7124	P01375	tumor ne	signaling	0.8046	0.263	0.966	C0948008
Ischemic stroke	C0948008	Amyotrophic Lateral Sclerosis	C0002736	TP53	7157	P04637	tumor pr	transcrip	0.16862	0.251	0.897	C0948008

Additional file5 Shared genes of dementia with IS												
Index_di	Index_di	Associated_disease	Associat	Gene	Gene_id	UniProt	Gene_Ful	Protein_(	pLI	DSI_g	DPI_g	diseaseid1
Ischemic	C0948008	Dementia	C0497327	NINJ2	4815	Q9NZG7	ninjurin	cell adhe	0.001235	0.834	0.103	C0948008
Ischemic	C0948008	Dementia	C0497327	F11	2160	P03951	coagulat	hydrolase	5.09E-26	0.656	0.414	C0948008
Ischemic	C0948008	Dementia	C0497327	SERPINI1	5274	Q99574	serpin fa	enzyme mo	0.029902	0.642	0.31	C0948008
Ischemic	C0948008	Dementia	C0497327	PON2	5445	Q15165	paraoxonase 2		6.06E-05	0.596	0.621	C0948008
Ischemic	C0948008	Dementia	C0497327	PLA2G7	7941	Q13093	phosphol	hydrolase	8.88E-22	0.584	0.552	C0948008
Ischemic	C0948008	Dementia	C0497327	TRPM7	54822	Q96QT4	transien	receptor	1.72E-15	0.584	0.552	C0948008
Ischemic	C0948008	Dementia	C0497327	PINK1	65018	Q9BXM7	PTEN ind	kinase; i	1.03E-10	0.577	0.586	C0948008
Ischemic	C0948008	Dementia	C0497327	SERPINA3	12	P01011	serpin fa	enzyme mo	8.07E-14	0.572	0.69	C0948008
Ischemic	C0948008	Dementia	C0497327	TREM2	54209	Q9NZC2	triggering recept		4.00E-09	0.572	0.552	C0948008
Ischemic	C0948008	Dementia	C0497327	CETP	1071	P11597	cholesteryl ester		1.03E-23	0.555	0.655	C0948008
Ischemic	C0948008	Dementia	C0497327	MOK	5891	Q9UQ07	MOK prot	kinase; t	9.92E-22	0.554	0.655	C0948008
Ischemic	C0948008	Dementia	C0497327	ACE2	59272	Q9BYF1	angioten	hydrolase	0.99813	0.542	0.655	C0948008
Ischemic	C0948008	Dementia	C0497327	PPIG	9360	Q13427	peptidylprolyl is		0.9649	0.537	0.793	C0948008
Ischemic	C0948008	Dementia	C0497327	PIN1	5300	Q13526	peptidylprolyl ci		0.76129	0.536	0.655	C0948008
Ischemic	C0948008	Dementia	C0497327	LRP1	4035	Q07954	LDL receptor rela		1	0.533	0.724	C0948008
Ischemic	C0948008	Dementia	C0497327	MSC	9242	O60682	musculin		0.022585	0.526	0.655	C0948008
Ischemic	C0948008	Dementia	C0497327	COL4A1	1282	P02462	collagen type IV		1	0.512	0.69	C0948008
Ischemic	C0948008	Dementia	C0497327	EEF1A2	1917	Q05639	eukaryot	enzyme mo	0.99606	0.51	0.69	C0948008
Ischemic	C0948008	Dementia	C0497327	GDF15	9518	Q99988	growth d	signaling	2.85E-07	0.496	0.621	C0948008
Ischemic	C0948008	Dementia	C0497327	PLA2G6	8398	O60733	phosphol	enzyme mo	2.79E-10	0.494	0.724	C0948008
Ischemic	C0948008	Dementia	C0497327	LPA	4018	P08519	lipoprot	hydrolase	5.81E-88	0.489	0.655	C0948008
Ischemic	C0948008	Dementia	C0497327	ABCA1	19	O95477	ATP bind	transport	3.53E-12	0.484	0.793	C0948008
Ischemic	C0948008	Dementia	C0497327	KCNA3	3738	P22001	potassium voltage		0.86034	0.481	0.724	C0948008
Ischemic	C0948008	Dementia	C0497327	SELE	6401	P16581	selectin E		7.02E-10	0.479	0.793	C0948008
Ischemic	C0948008	Dementia	C0497327	APOB	338	P04114	apolipoprotein B		1.64E-16	0.477	0.724	C0948008
Ischemic	C0948008	Dementia	C0497327	PRNP	5621	F7VJQ1;P(	prion protein		0.000715	0.474	0.897	C0948008
Ischemic	C0948008	Dementia	C0497327	AGER	177	Q15109	advanced	cell adhe	3.44E-16	0.472	0.724	C0948008
Ischemic	C0948008	Dementia	C0497327	ADM	133	P35318	adrenome	signaling	0.034615	0.471	0.724	C0948008
Ischemic	C0948008	Dementia	C0497327	NOTCH3	4854	Q9UM47	notch 3		0.86965	0.469	0.69	C0948008
Ischemic	C0948008	Dementia	C0497327	VCAM1	7412	P19320	vascular cell adh		0.74466	0.466	0.793	C0948008
Ischemic	C0948008	Dementia	C0497327	PPARA	5465	Q07869	peroxiso	nucleic a	0.037307	0.465	0.793	C0948008

Ischemic	C0948008	Dementia	C0497327	APOA1	335	P02647	apolipoprotein A1		0.000549	0.463	0.759	C0948008
Ischemic	C0948008	Dementia	C0497327	ESR2	2100	Q92731	estrogen	nucleic a	5.39E-08	0.445	0.828	C0948008
Ischemic	C0948008	Dementia	C0497327	PON1	5444	P27169	paraoxonase 1		8.98E-11	0.443	0.828	C0948008
Ischemic	C0948008	Dementia	C0497327	HFE	3077	Q30201	homeostatic iron		3.01E-08	0.44	0.69	C0948008
Ischemic	C0948008	Dementia	C0497327	MMP3	4314	P08254	matrix me	hydrolase	4.93E-15	0.439	0.793	C0948008
Ischemic	C0948008	Dementia	C0497327	IGF1R	3480	P08069	insulin like grow		0.98751	0.433	0.828	C0948008
Ischemic	C0948008	Dementia	C0497327	BRCA2	675	P51587	BRCA2, DN	nucleic a	5.46E-24	0.426	0.793	C0948008
Ischemic	C0948008	Dementia	C0497327	COMT	1312	P21964	catechol-	transfera	1.27E-05	0.426	0.897	C0948008
Ischemic	C0948008	Dementia	C0497327	REN	5972	P00797	renin	hydrolase	2.12E-07	0.423	0.759	C0948008
Ischemic	C0948008	Dementia	C0497327	MIR21	406991		microRNA 21			0.407	0.759	C0948008
Ischemic	C0948008	Dementia	C0497327	IL18	3606	Q14116	interleukin 18		0.029184	0.404	0.862	C0948008
Ischemic	C0948008	Dementia	C0497327	NOS3	4846	P29474	nitric oxide synt		8.89E-07	0.401	0.862	C0948008
Ischemic	C0948008	Dementia	C0497327	CRP	1401	P02741	C-reactive protei		0.003736	0.399	0.862	C0948008
Ischemic	C0948008	Dementia	C0497327	IL1RN	3557	P18510	interleukin 1 rec		0.033509	0.398	0.897	C0948008
Ischemic	C0948008	Dementia	C0497327	COX2	4513	P00403	cytochrom	oxidoreductase		0.397	0.862	C0948008
Ischemic	C0948008	Dementia	C0497327	SPP1	6696	P10451	secreted phosphop		1.05E-06	0.391	0.862	C0948008
Ischemic	C0948008	Dementia	C0497327	PPARG	5468	P37231	peroxison	nucleic a	0.1447	0.388	0.828	C0948008
Ischemic	C0948008	Dementia	C0497327	ICAM1	3383	P05362	intercellular adh		0.038102	0.386	0.897	C0948008
Ischemic	C0948008	Dementia	C0497327	SERPINE1	5054	P05121	serpin fa	enzyme mo	0.047491	0.384	0.793	C0948008
Ischemic	C0948008	Dementia	C0497327	BDNF	627	P23560	brain der	signaling	0.69969	0.382	0.828	C0948008
Ischemic	C0948008	Dementia	C0497327	ACE	1636	P12821	angiotens	hydrolase	1.55E-37	0.367	0.897	C0948008
Ischemic	C0948008	Dementia	C0497327	IL1A	3552	P01583	interleukin 1 alp		0.000149	0.366	0.931	C0948008
Ischemic	C0948008	Dementia	C0497327	ESR1	2099	P03372	estrogen	nucleic a	0.9994	0.355	0.897	C0948008
Ischemic	C0948008	Dementia	C0497327	IGF1	3479	P05019	insulin like grow		0.28843	0.355	0.828	C0948008
Ischemic	C0948008	Dementia	C0497327	MTHFR	4524	P42898	methylenetetrahyd		1.22E-09	0.354	0.828	C0948008
Ischemic	C0948008	Dementia	C0497327	APOE	348	P02649	apolipoprotein E		0.001737	0.352	0.931	C0948008
Ischemic	C0948008	Dementia	C0497327	CXCL8	3576	P10145	C-X-C mo	signaling molecul		0.342	0.862	C0948008
Ischemic	C0948008	Dementia	C0497327	PTGS2	5743	P35354	prostagla	oxidoredu	0.99639	0.338	0.897	C0948008
Ischemic	C0948008	Dementia	C0497327	TGFB1	7040	P01137	transforn	signaling	0.17182	0.336	0.931	C0948008
Ischemic	C0948008	Dementia	C0497327	IL1B	3553	P01584	interleukin 1 bet		0.12568	0.312	0.931	C0948008
Ischemic	C0948008	Dementia	C0497327	IL6	3569	P05231	interleukin 6		0.33873	0.287	0.966	C0948008
Ischemic	C0948008	Dementia	C0497327	TNF	7124	P01375	tumor nec	signaling	0.8046	0.263	0.966	C0948008
Ischemic	C0948008	Dementia	C0497327	TP53	7157	P04637	tumor pro	transcrip	0.16862	0.251	0.897	C0948008

Additional file5 Shared genes of epilepsy with IS

Index_disease	Index_di	Associated_disease	Associat	Gene	Gene_id	UniProt	Gene_Ful	Protein_(	pLI	DSI_g	DPI_g	diseaseic
Ischemic stroke	C0948008	Epilepsy	C0014544	KCNJ13	3769	O60928	potassium voltage		0.011166	0.681	0.172	C0948008
Ischemic stroke	C0948008	Epilepsy	C0014544	GAMT	2593	Q14353	guanidinoacetate		0.010013	0.667	0.241	C0948008
Ischemic stroke	C0948008	Epilepsy	C0014544	GRM2	2912	Q14416	glutamat	receptor	0.1063	0.645	0.414	C0948008
Ischemic stroke	C0948008	Epilepsy	C0014544	COL4A2	1284	P08572	collagen type IV		1.04E-13	0.642	0.448	C0948008
Ischemic stroke	C0948008	Epilepsy	C0014544	SERPINI1	5274	Q99574	serpin fa	enzyme mo	0.029902	0.642	0.31	C0948008
Ischemic stroke	C0948008	Epilepsy	C0014544	GRIA2	2891	P42262	glutamate ionotro		0.99906	0.639	0.414	C0948008
Ischemic stroke	C0948008	Epilepsy	C0014544	GRIN1	2902	Q05586	glutamate ionotro		0.98871	0.63	0.448	C0948008
Ischemic stroke	C0948008	Epilepsy	C0014544	GRIK2	2898	Q13002	glutamate ionotro		0.99979	0.621	0.586	C0948008
Ischemic stroke	C0948008	Epilepsy	C0014544	MUSK	4593	O15146	muscle associated		1.17E-07	0.609	0.621	C0948008
Ischemic stroke	C0948008	Epilepsy	C0014544	SRF	6722	P11831	serum res	transcrip	0.97512	0.598	0.69	C0948008
Ischemic stroke	C0948008	Epilepsy	C0014544	GLA	2717	P06280	galactosidase alp		0.99705	0.588	0.621	C0948008
Ischemic stroke	C0948008	Epilepsy	C0014544	AHSG	197	P02765	alpha 2-H	enzyme mo	2.72E-10	0.577	0.69	C0948008
Ischemic stroke	C0948008	Epilepsy	C0014544	PINK1	65018	Q9BXM7	PTEN indu	kinase; t	1.03E-10	0.577	0.586	C0948008
Ischemic stroke	C0948008	Epilepsy	C0014544	SLC1A2	6506	P43004	solute ca	transport	0.61687	0.572	0.517	C0948008
Ischemic stroke	C0948008	Epilepsy	C0014544	TREM2	54209	Q9NZC2	triggering recept		4.00E-09	0.572	0.552	C0948008
Ischemic stroke	C0948008	Epilepsy	C0014544	ACVRL1	94	P37023	activin A	kinase; t	0.000823	0.57	0.621	C0948008
Ischemic stroke	C0948008	Epilepsy	C0014544	MIR132	406921		microRNA 132			0.569	0.759	C0948008
Ischemic stroke	C0948008	Epilepsy	C0014544	PC	5091	P11498	pyruvate	ligase	0.007175	0.565	0.724	C0948008
Ischemic stroke	C0948008	Epilepsy	C0014544	C20orf181	1E+08		chromosome 20 open reading			0.557	0.69	C0948008
Ischemic stroke	C0948008	Epilepsy	C0014544	ADRA2B	151	P18089	adrenocep	receptor	2.20E-06	0.545	0.69	C0948008
Ischemic stroke	C0948008	Epilepsy	C0014544	NR4A3	8013	Q92570	nuclear r	receptor	0.89536	0.545	0.759	C0948008
Ischemic stroke	C0948008	Epilepsy	C0014544	CYP2C9	1559	P11712	cytochrome P450 f		1.80E-10	0.542	0.759	C0948008
Ischemic stroke	C0948008	Epilepsy	C0014544	ACTA2	59	P62736	actin, a	cytoskele	0.9299	0.54	0.69	C0948008
Ischemic stroke	C0948008	Epilepsy	C0014544	PPIG	9360	Q13427	peptidylprolyl is		0.9649	0.537	0.793	C0948008
Ischemic stroke	C0948008	Epilepsy	C0014544	GCH1	2643	P30793	GTP cycl	hydrolase	0.91089	0.534	0.724	C0948008
Ischemic stroke	C0948008	Epilepsy	C0014544	FGF13	2258	Q92913	fibroblas	signaling	0.90603	0.532	0.621	C0948008
Ischemic stroke	C0948008	Epilepsy	C0014544	SGK1	6446	O00141	serum/glu	calcium-b	0.040926	0.53	0.621	C0948008
Ischemic stroke	C0948008	Epilepsy	C0014544	UCP2	7351	P55851	uncoupling protei		1.35E-10	0.53	0.69	C0948008
Ischemic stroke	C0948008	Epilepsy	C0014544	AQP4	361	P55087	aquaporin	transport	0.002775	0.525	0.793	C0948008
Ischemic stroke	C0948008	Epilepsy	C0014544	KCNQ1	3784	P51787	potassium voltage		2.07E-07	0.525	0.586	C0948008
Ischemic stroke	C0948008	Epilepsy	C0014544	COL4A1	1282	P02462	collagen type IV		1	0.512	0.69	C0948008

Ischemic stroke	C0948008	Epilepsy	C0014544	EEF1A2	1917	Q05639	eukaryoti	enzyme mo	0.99606	0.51	0.69	C0948008
Ischemic stroke	C0948008	Epilepsy	C0014544	MTRR	4552	Q9UBK8	5-methyltetrahydr		1.77E-14	0.51	0.655	C0948008
Ischemic stroke	C0948008	Epilepsy	C0014544	ABCC8	6833	Q09428	ATP bindi	transport	2.72E-23	0.508	0.69	C0948008
Ischemic stroke	C0948008	Epilepsy	C0014544	NRG1	3084	Q02297	neureguli	signaling	0.99553	0.506	0.759	C0948008
Ischemic stroke	C0948008	Epilepsy	C0014544	PLA2G1B	5319	P04054	phospholi	hydrolase	1.09E-10	0.502	0.793	C0948008
Ischemic stroke	C0948008	Epilepsy	C0014544	CYP2C19	1557	P33261	cytochrome P450 f		4.11E-20	0.501	0.793	C0948008
Ischemic stroke	C0948008	Epilepsy	C0014544	HDAC9	9734	Q9UKV0	histone deacetyla		0.99996	0.501	0.759	C0948008
Ischemic stroke	C0948008	Epilepsy	C0014544	PLA2G6	8398	O60733	phospholi	enzyme mo	2.79E-10	0.494	0.724	C0948008
Ischemic stroke	C0948008	Epilepsy	C0014544	MTR	4548	Q99707	5-methyltetrahydr		2.65E-10	0.493	0.724	C0948008
Ischemic stroke	C0948008	Epilepsy	C0014544	KCNA3	3738	P22001	potassium voltage		0.86034	0.481	0.724	C0948008
Ischemic stroke	C0948008	Epilepsy	C0014544	ENG	2022	P17813	endoglin		0.99877	0.477	0.655	C0948008
Ischemic stroke	C0948008	Epilepsy	C0014544	PRNP	5621	F7VJQ1;P(	prion protein		0.000715	0.474	0.897	C0948008
Ischemic stroke	C0948008	Epilepsy	C0014544	AGER	177	Q15109	advanced	cell adhe	3.44E-16	0.472	0.724	C0948008
Ischemic stroke	C0948008	Epilepsy	C0014544	NPY	4852	P01303	neuropept	signaling	0.15508	0.469	0.69	C0948008
Ischemic stroke	C0948008	Epilepsy	C0014544	NOTCH3	4854	Q9UM47	notch 3		0.86965	0.469	0.69	C0948008
Ischemic stroke	C0948008	Epilepsy	C0014544	SEMA6A	57556	Q9H2E6	semaphori	signaling	0.99993	0.468	0.793	C0948008
Ischemic stroke	C0948008	Epilepsy	C0014544	COX8A	1351	P10176	cytochrom	oxidoredu	0.10544	0.461	0.793	C0948008
Ischemic stroke	C0948008	Epilepsy	C0014544	EGR1	1958	P18146	early gro	nucleic a	0.3269	0.459	0.793	C0948008
Ischemic stroke	C0948008	Epilepsy	C0014544	PLAT	5327	P00750	plasminog	hydrolase	3.46E-05	0.459	0.828	C0948008
Ischemic stroke	C0948008	Epilepsy	C0014544	MIR146A	406938		microRNA 146a			0.452	0.759	C0948008
Ischemic stroke	C0948008	Epilepsy	C0014544	ABCG2	9429	Q9UNQ0	ATP bindi	transport	1.26E-32	0.449	0.793	C0948008
Ischemic stroke	C0948008	Epilepsy	C0014544	HSPA4	3308	P34932	heat shock protei		0.9999	0.441	0.793	C0948008
Ischemic stroke	C0948008	Epilepsy	C0014544	CYP2B6	1555	P20813	cytochrome P450 f		3.50E-10	0.437	0.828	C0948008
Ischemic stroke	C0948008	Epilepsy	C0014544	TBC1D9	23158	Q6ZT07	TBC1 doma	enzyme mo	2.83E-05	0.434	0.828	C0948008
Ischemic stroke	C0948008	Epilepsy	C0014544	HMGB1	3146	P09429	high mobi	nucleic a	0.83018	0.433	0.828	C0948008
Ischemic stroke	C0948008	Epilepsy	C0014544	COMT	1312	P21964	catechol-	transfera	1.27E-05	0.426	0.897	C0948008
Ischemic stroke	C0948008	Epilepsy	C0014544	EPHB2	2048	P29323	EPH receptor B2		0.99999	0.425	0.759	C0948008
Ischemic stroke	C0948008	Epilepsy	C0014544	REN	5972	P00797	renin	hydrolase	2.12E-07	0.423	0.759	C0948008
Ischemic stroke	C0948008	Epilepsy	C0014544	EZH2	2146	Q15910	enhancer of zeste		1	0.421	0.759	C0948008
Ischemic stroke	C0948008	Epilepsy	C0014544	PLG	5340	P00747	plasminog	hydrolase	0.009642	0.419	0.862	C0948008
Ischemic stroke	C0948008	Epilepsy	C0014544	MIR21	406991		microRNA 21			0.407	0.759	C0948008
Ischemic stroke	C0948008	Epilepsy	C0014544	ATM	472	Q13315	ATM seri	kinase; r	5.98E-46	0.401	0.862	C0948008
Ischemic stroke	C0948008	Epilepsy	C0014544	IL1RN	3557	P18510	interleukin 1 rec		0.033509	0.398	0.897	C0948008
Ischemic stroke	C0948008	Epilepsy	C0014544	IL17A	3605	Q16552	interleukin 17A		0.044037	0.391	0.862	C0948008
Ischemic stroke	C0948008	Epilepsy	C0014544	MAPK1	5594	P28482	mitogen-a	kinase; t	0.9973	0.387	0.862	C0948008
Ischemic stroke	C0948008	Epilepsy	C0014544	BDNF	627	P23560	brain der	signaling	0.69969	0.382	0.828	C0948008

Ischemic stroke	C0948008	Epilepsy	C0014544	ABCB1	5243	P08183	ATP bindi	hydrolase	1.31E-05	0.371	0.828	C0948008
Ischemic stroke	C0948008	Epilepsy	C0014544	IL1A	3552	P01583	interleukin 1 alp		0.000149	0.366	0.931	C0948008
Ischemic stroke	C0948008	Epilepsy	C0014544	TLR4	7099	O00206	toll like recepto		5.58E-09	0.359	0.931	C0948008
Ischemic stroke	C0948008	Epilepsy	C0014544	ESR1	2099	P03372	estrogen	nucleic a	0.9994	0.355	0.897	C0948008
Ischemic stroke	C0948008	Epilepsy	C0014544	MTHFR	4524	P42898	methylenetetrahyd		1.22E-09	0.354	0.828	C0948008
Ischemic stroke	C0948008	Epilepsy	C0014544	APOE	348	P02649	apolipoprotein E		0.001737	0.352	0.931	C0948008
Ischemic stroke	C0948008	Epilepsy	C0014544	TGFB1	7040	P01137	transform	signaling	0.17182	0.336	0.931	C0948008
Ischemic stroke	C0948008	Epilepsy	C0014544	IL1B	3553	P01584	interleukin 1 bet		0.12568	0.312	0.931	C0948008
Ischemic stroke	C0948008	Epilepsy	C0014544	IL6	3569	P05231	interleukin 6		0.33873	0.287	0.966	C0948008
Ischemic stroke	C0948008	Epilepsy	C0014544	TP53	7157	P04637	tumor pro	transcrip	0.16862	0.251	0.897	C0948008

Additional file5 Shared genes of MDD with IS												
Index_disease	Index_di	Associated_disease	Associat	Gene	Gene_id	UniProt	Gene_Ful	Protein_(	pLI	DSI_g	DPI_g	diseasei
Ischemic stroke	C0948008	Major Depressive Disorder	C1269683	KALRN	8997	O60229	kalirin I	signaling	1	0.72	0.207	C0948008
Ischemic stroke	C0948008	Major Depressive Disorder	C1269683	HOMER1	9456	Q86YM7	homer sca	signaling	0.99937	0.69	0.241	C0948008
Ischemic stroke	C0948008	Major Depressive Disorder	C1269683	GRM2	2912	Q14416	glutamat	receptor	0.1063	0.645	0.414	C0948008
Ischemic stroke	C0948008	Major Depressive Disorder	C1269683	GRIA2	2891	P42262	glutamate ionotro		0.99906	0.639	0.414	C0948008
Ischemic stroke	C0948008	Major Depressive Disorder	C1269683	TNFSF12-TNFSF13	407977	O43508	TNFSF12-TNFSF13 r		0.75747	0.639	0.517	C0948008
Ischemic stroke	C0948008	Major Depressive Disorder	C1269683	FADS1	3992	O60427	fatty acid desatu		0.41321	0.636	0.621	C0948008
Ischemic stroke	C0948008	Major Depressive Disorder	C1269683	PDE11A	50940	Q9HCR9	phosphodiesterase		1.57E-34	0.627	0.517	C0948008
Ischemic stroke	C0948008	Major Depressive Disorder	C1269683	REM1	28954	O75628	RRAD and GEM like		6.77E-08	0.621	0.655	C0948008
Ischemic stroke	C0948008	Major Depressive Disorder	C1269683	CRTC1	23373	Q6UUV9	CREB reg	transcri	0.42733	0.602	0.621	C0948008
Ischemic stroke	C0948008	Major Depressive Disorder	C1269683	TNFSF12	8742	O43508	TNF superfamily m		0.75747	0.598	0.655	C0948008
Ischemic stroke	C0948008	Major Depressive Disorder	C1269683	REST	5978	Q13127	RE1 siler	transcri	0.99393	0.584	0.517	C0948008
Ischemic stroke	C0948008	Major Depressive Disorder	C1269683	GPR42	2866	O15529	G protein-coupled		0.047652	0.577	0.69	C0948008
Ischemic stroke	C0948008	Major Depressive Disorder	C1269683	PINK1	65018	Q9BXM7	PTEN ind	kinase;	1.03E-10	0.577	0.586	C0948008
Ischemic stroke	C0948008	Major Depressive Disorder	C1269683	IL20	50604	Q9NYY1	interleukin 20		1.72E-06	0.575	0.69	C0948008
Ischemic stroke	C0948008	Major Depressive Disorder	C1269683	SLC1A2	6506	P43004	solute ca	transpor	0.61687	0.572	0.517	C0948008
Ischemic stroke	C0948008	Major Depressive Disorder	C1269683	BRS3	680	P32247	bombesin receptor		0.90963	0.569	0.69	C0948008
Ischemic stroke	C0948008	Major Depressive Disorder	C1269683	SSTR4	6754	P31391	somatosta	receptor	2.77E-13	0.558	0.69	C0948008
Ischemic stroke	C0948008	Major Depressive Disorder	C1269683	ADRA1A	148	P35348	adrenocep	receptor	2.60E-07	0.548	0.69	C0948008
Ischemic stroke	C0948008	Major Depressive Disorder	C1269683	ITGA2B	3674	P08514	integrin subunit		1.35E-15	0.548	0.517	C0948008
Ischemic stroke	C0948008	Major Depressive Disorder	C1269683	ADRA2B	151	P18089	adrenocep	receptor	2.20E-06	0.545	0.69	C0948008
Ischemic stroke	C0948008	Major Depressive Disorder	C1269683	CDK5	1020	Q00535	cyclin d	kinase;	0.095196	0.542	0.69	C0948008
Ischemic stroke	C0948008	Major Depressive Disorder	C1269683	CYP2C9	1559	P11712	cytochrome P450 f		1.80E-10	0.542	0.759	C0948008
Ischemic stroke	C0948008	Major Depressive Disorder	C1269683	TLR7	51284	Q9NYK1	toll like recepto		0.97165	0.542	0.759	C0948008
Ischemic stroke	C0948008	Major Depressive Disorder	C1269683	LPAR2	9170	Q9HBW0	lysophos	receptor	0.04354	0.537	0.69	C0948008
Ischemic stroke	C0948008	Major Depressive Disorder	C1269683	GCH1	2643	P30793	GTP cycl	hydrolas	0.91089	0.534	0.724	C0948008
Ischemic stroke	C0948008	Major Depressive Disorder	C1269683	GNB3	2784	P16520	G protei	enzyme m	2.92E-11	0.533	0.724	C0948008
Ischemic stroke	C0948008	Major Depressive Disorder	C1269683	LRP1	4035	Q07954	LDL receptor rela		1	0.533	0.724	C0948008
Ischemic stroke	C0948008	Major Depressive Disorder	C1269683	FGF13	2258	Q92913	fibrobla	signaling	0.90603	0.532	0.621	C0948008
Ischemic stroke	C0948008	Major Depressive Disorder	C1269683	SGK1	6446	O00141	serum/gl	calcium-	0.040926	0.53	0.621	C0948008
Ischemic stroke	C0948008	Major Depressive Disorder	C1269683	NTF3	4908	P20783	neurotro	signaling	0.88635	0.525	0.621	C0948008
Ischemic stroke	C0948008	Major Depressive Disorder	C1269683	CXCR6	10663	O00574	C-X-C motif chemo		5.33E-05	0.525	0.793	C0948008
Ischemic stroke	C0948008	Major Depressive Disorder	C1269683	NR3C2	4306	P08235	nuclear	nucleic a	0.80843	0.52	0.69	C0948008
Ischemic stroke	C0948008	Major Depressive Disorder	C1269683	HSPA1A	3303	P0DMV8;P	heat shock protei		0.029335	0.516	0.793	C0948008
Ischemic stroke	C0948008	Major Depressive Disorder	C1269683	HSPA1B	3304	P0DMV8;P	heat shock protei		0.005091	0.514	0.793	C0948008

Ischemic stroke	C0948008	Major Depressive Disorder	C1269683	NRG1	3084	Q02297	neuregul	signaling	0.99553	0.506	0.759	C0948008
Ischemic stroke	C0948008	Major Depressive Disorder	C1269683	P2RX7	5027	Q99572	purinerg	receptor	3.05E-11	0.503	0.759	C0948008
Ischemic stroke	C0948008	Major Depressive Disorder	C1269683	CYP2C19	1557	P33261	cytochrome P450 f		4.11E-20	0.501	0.793	C0948008
Ischemic stroke	C0948008	Major Depressive Disorder	C1269683	ACKR3	57007	P25106	atypical chemokin		0.37575	0.494	0.759	C0948008
Ischemic stroke	C0948008	Major Depressive Disorder	C1269683	AVP	551	P01185	arginine	signaling	0.075195	0.486	0.759	C0948008
Ischemic stroke	C0948008	Major Depressive Disorder	C1269683	F2R	2149	P25116	coagulation facto		0.00577	0.478	0.759	C0948008
Ischemic stroke	C0948008	Major Depressive Disorder	C1269683	EDNRA	1909	P25101	endothelin recept		0.99154	0.469	0.828	C0948008
Ischemic stroke	C0948008	Major Depressive Disorder	C1269683	NPY	4852	P01303	neuropep	signaling	0.15508	0.469	0.69	C0948008
Ischemic stroke	C0948008	Major Depressive Disorder	C1269683	NQO1	1728	P15559	NAD(P)H quinone d		1.02E-09	0.467	0.724	C0948008
Ischemic stroke	C0948008	Major Depressive Disorder	C1269683	LTA	4049	P01374	lymphoto	signaling	0.55529	0.463	0.862	C0948008
Ischemic stroke	C0948008	Major Depressive Disorder	C1269683	EGR1	1958	P18146	early gr	nucleic a	0.3269	0.459	0.793	C0948008
Ischemic stroke	C0948008	Major Depressive Disorder	C1269683	ESR2	2100	Q92731	estrogen	nucleic a	5.39E-08	0.445	0.828	C0948008
Ischemic stroke	C0948008	Major Depressive Disorder	C1269683	PON1	5444	P27169	paraoxonase 1		8.98E-11	0.443	0.828	C0948008
Ischemic stroke	C0948008	Major Depressive Disorder	C1269683	HSPA4	3308	P34932	heat shock protei		0.9999	0.441	0.793	C0948008
Ischemic stroke	C0948008	Major Depressive Disorder	C1269683	MPO	4353	P05164	myeloper	oxidoredu	6.82E-15	0.435	0.931	C0948008
Ischemic stroke	C0948008	Major Depressive Disorder	C1269683	TBC1D9	23158	Q6ZT07	TBC1 doma	enzyme mo	2.83E-05	0.434	0.828	C0948008
Ischemic stroke	C0948008	Major Depressive Disorder	C1269683	COMT	1312	P21964	catechol-	transfera	1.27E-05	0.426	0.897	C0948008
Ischemic stroke	C0948008	Major Depressive Disorder	C1269683	EPHB2	2048	P29323	EPH receptor B2		0.99999	0.425	0.759	C0948008
Ischemic stroke	C0948008	Major Depressive Disorder	C1269683	KDR	3791	P35968	kinase insert dom		0.99979	0.423	0.724	C0948008
Ischemic stroke	C0948008	Major Depressive Disorder	C1269683	REN	5972	P00797	renin	hydrolase	2.12E-07	0.423	0.759	C0948008
Ischemic stroke	C0948008	Major Depressive Disorder	C1269683	PLG	5340	P00747	plasmino	hydrolase	0.009642	0.419	0.862	C0948008
Ischemic stroke	C0948008	Major Depressive Disorder	C1269683	NOTCH1	4851	P46531	notch 1		1	0.415	0.759	C0948008
Ischemic stroke	C0948008	Major Depressive Disorder	C1269683	FGF2	2247	P09038	fibrobla	signaling	0.019149	0.404	0.897	C0948008
Ischemic stroke	C0948008	Major Depressive Disorder	C1269683	NOS3	4846	P29474	nitric oxide synt		8.89E-07	0.401	0.862	C0948008
Ischemic stroke	C0948008	Major Depressive Disorder	C1269683	CRP	1401	P02741	C-reactive protei		0.003736	0.399	0.862	C0948008
Ischemic stroke	C0948008	Major Depressive Disorder	C1269683	MAPK1	5594	P28482	mitogen-a	kinase;	0.9973	0.387	0.862	C0948008
Ischemic stroke	C0948008	Major Depressive Disorder	C1269683	SERPINE1	5054	P05121	serpin fa	enzyme mo	0.047491	0.384	0.793	C0948008
Ischemic stroke	C0948008	Major Depressive Disorder	C1269683	BDNF	627	P23560	brain de	signaling	0.69969	0.382	0.828	C0948008
Ischemic stroke	C0948008	Major Depressive Disorder	C1269683	ABCB1	5243	P08183	ATP bind	hydrolase	1.31E-05	0.371	0.828	C0948008
Ischemic stroke	C0948008	Major Depressive Disorder	C1269683	ACE	1636	P12821	angioten	hydrolase	1.55E-37	0.367	0.897	C0948008
Ischemic stroke	C0948008	Major Depressive Disorder	C1269683	IL1A	3552	P01583	interleukin 1 alp		0.000149	0.366	0.931	C0948008
Ischemic stroke	C0948008	Major Depressive Disorder	C1269683	ESR1	2099	P03372	estrogen	nucleic a	0.9994	0.355	0.897	C0948008
Ischemic stroke	C0948008	Major Depressive Disorder	C1269683	MTHFR	4524	P42898	methylenetetrahyd		1.22E-09	0.354	0.828	C0948008
Ischemic stroke	C0948008	Major Depressive Disorder	C1269683	APOE	348	P02649	apolipoprotein E		0.001737	0.352	0.931	C0948008
Ischemic stroke	C0948008	Major Depressive Disorder	C1269683	PTGS2	5743	P35354	prostagl	oxidoredu	0.99639	0.338	0.897	C0948008
Ischemic stroke	C0948008	Major Depressive Disorder	C1269683	CDKN2A	1029	P42771;Q8	cyclin dependent		0.38138	0.321	0.828	C0948008
Ischemic stroke	C0948008	Major Depressive Disorder	C1269683	IL10	3586	P22301	interleukin 10		0.006506	0.32	0.931	C0948008
Ischemic stroke	C0948008	Major Depressive Disorder	C1269683	IL1B	3553	P01584	interleukin 1 bet		0.12568	0.312	0.931	C0948008

Ischemic stroke	C0948008	Major Depressive Disorder	C1269683	VEGFA	7422	P15692	vascular	signaling	2.65E-05	0.298	0.897	C0948008
Ischemic stroke	C0948008	Major Depressive Disorder	C1269683	IL6	3569	P05231	interleukin 6		0.33873	0.287	0.966	C0948008
Ischemic stroke	C0948008	Major Depressive Disorder	C1269683	TNF	7124	P01375	tumor ne	signaling	0.8046	0.263	0.966	C0948008

Additional file6 Six conformations of GRIN1 in molecular docking with NBP

receptor	conformation	Chain	binding area	affinity(kcal/mol)
GRIN1heterotetramer	11	C	ABD	-6.7
GRIN1heterotetramer	12	AB	interface of chain A and B in ABD	-6.7
GRIN1heterotetramer	14	A	ABD	-6.6
GRIN1heterotetramer	15	AD	interface of chain A and D in ABD	-6.6
GRIN1heterotetramer	17	AB	interface of chain A and B in ABD	-6.5
GRIN1heterotetramer	18	BC	interface of chain B and C in ABD	-6.4

GRIN1:chain A and C;GRIN2A:chain B and D; ABD:agonist binding domain
